# The class VIII myosin ATM2 stabilizes actin–plasma membrane contacts in tobacco pollen tubes by locally promoting PtdIns(4,5)P_2_ formation

**DOI:** 10.1038/s41477-026-02427-0

**Published:** 2026-10-16

**Authors:** Vera C. Wagner, Marta Fratini, Johanna Uhlenberg, Alexandra Schutkowski, Gottfried Hamm, Christoph Kastner, Kirsten Bacia, Kateřina Schwarzerová, Ingo Heilmann

**Affiliations:** 1 Department of Plant Biochemistry, Institute of Biochemistry and Biotechnology, Martin Luther University Halle-Wittenberg, Halle (Saale), Germany; 2 Department of Biophysical Chemistry, Institute of Chemistry, Martin Luther University Halle-Wittenberg, Halle (Saale), Germany; 3 Department of Experimental Plant Biology, Faculty of Science, Charles University, Prague, Czech Republic

**Keywords:** Lipid signalling, Phosphoinositol signalling

## Abstract

The intersection of actin filaments with the plasma membrane (PM) in plant cells involves lipid–protein interactions by the *Arabidopsis* class VIII myosin ATM2 that are currently not understood. Using pollen tube cells as a model, we describe how ATM2 enables actin–PM contacts by modulating membrane lipid nano-organization. ATM2 bound anionic PM lipids, including phosphatidylinositol 4,5-bisphosphate (PtdIns(4,5)P_2_), with no apparent preference for any particular lipid species. Overexpression of ATM2 unexpectedly stabilized actin dynamics and altered pollen tube morphology. Actin dynamics remained unaltered upon overexpression of a lipid-binding-defective ATM2 variant. ATM2 effects on actin dynamics required PtdIns(4,5)P_2_, as artificial PtdIns(4,5)P_2_ depletion in pollen tubes abolished the effects of full-length ATM2 on actin dynamics despite its retained PM association. ATM2 colocalized and interacted with the PI4P 5-kinase PIP5K2, which resides at actin–PM contacts and forms PtdIns(4,5)P_2_ nanodomains facilitating rho-of-plants-dependent actin stabilization. Upon ATM2 overexpression, a fluorescent PtdIns(4,5)P_2_ biosensor decorated an extended PM region in vivo, indicating a promoting effect of ATM2 on PtdIns(4,5)P_2_ abundance. The catalytic activity of purified recombinant PIP5K2 protein was enhanced upon coincubation with a purified carboxy-terminal ATM2 fragment in vitro, suggesting that ATM2 contributes to the intrinsic regulation of PtdIns(4,5)P_2_ formation. Actin dynamics are thus stabilized at the actin–PM interface by ATM2 locally promoting the formation of PtdIns(4,5)P_2_ nanodomains, thereby self-reinforcing ATM2 recruitment while also facilitating rho of plants activation to stabilize membrane-proximal actin filaments.

## Abstract (web summary)

The *Arabidopsis* class VIII myosin ATM2 enables actin–plasma membrane contacts by locally promoting PtdIns(4,5)P2 formation. The modulation of plasma membrane lipids self-reinforces ATM2 recruitment and stabilizes membrane-proximal actin dynamics.

## Main

The extremely polarized morphology of pollen tubes and root hairs is a consequence of polar tip growth involving the focused transport of secretory vesicles towards a narrow plasma membrane (PM) area of the growing cell apex^1–5^. The vectorial transport of vesicles, their fusion with the target membrane area and the endocytic retrieval of vesicles upon cargo release require the coordination of membrane trafficking and cytoskeletal dynamics in the cell apex^1,2,6–9^. Apart from their respective biological roles in plant sexual reproduction^6,10,11^ and in root–rhizosphere interactions^4,12^, pollen tubes and root hairs are ideal models to study the underlying molecular mechanisms, as cell morphology is critically altered by perturbed coordination of membrane trafficking and cytoskeletal dynamics^13,14^.

In pollen tubes, the tip-directed trafficking machinery is focused towards a well-defined subapical PM domain enriched in the regulatory membrane lipid phosphatidylinositol 4,5-bisphosphate (PtdIns(4,5)P_2_)^6,15,16^. PtdIns(4,5)P_2_ serves as a positional signal for the recruitment of proteins to the cytosolic face of the PM and as such can exert different regulatory roles^17^, including the regulation of secretion^18–20^, endocytosis^21,22^ and the control of actin dynamics^23,24^. Pollen tube cells have been used extensively to study PtdIns(4,5)P_2_-mediated coordination of membrane trafficking and cytoskeletal dynamics, as perturbations in the balance of these processes give rise to characteristic changes in cell morphology, including tip swelling associated with perturbed cytoskeletal stability^23–26^. Reduced actin dynamics upon PtdIns(4,5)P_2_ overproduction has been shown in various biological systems, including mammalian cells^27,28^, yeast^29^ and plants^23,30^, and while the effects of PtdIns(4,5)P_2_ on actin dynamics are not limited to pollen tubes, the pollen tube model uniquely allows researchers to investigate the molecular mechanisms of PtdIns(4,5)P_2_-mediated regulation of actin dynamics that are currently not understood in plants.

Several regulatory elements are known to contribute to PtdIns(4,5)P_2_-dependent control of actin dynamics in plants, including monomeric rho of plants (ROP) GTPases^31,32^ and their effectors, such as guanine nucleotide dissociation inhibitors (GDIs)^31,33,34^, which control the pool of ROPs that can be activated by PM-associated guanine nucleotide exchange factors^31,35^, or ROP-interacting CRIB-domain-containing proteins^36,37^, representing key ROP targets. However, the effects of PtdIns(4,5)P_2_ on pollen tube growth are not limited to the control of ROPs and actin dynamics, and PtdIns(4,5)P_2_ has additional, alternative roles in the control of secretion and endocytosis^1,7,19,21,38^. It is currently not well understood how different cellular processes are specifically controlled by the little-abundant PM lipid PtdIns(4,5)P_2_.

One aspect guiding the coordination of alternative effects of PtdIns(4,5)P_2_ on apical secretion or on cytoskeletal dynamics is a subcompartmentalized PM nanostructure, represented by clustered and non-clustered distributions of proteins and lipids, including PtdIns(4,5)P_2_ (refs. ^23,39^). In the subapical PM zone of pollen tube cells, PtdIns(4,5)P_2_ occurs enriched in PM nanodomains that exert a focused stabilizing effect on actin dynamics^23^. PtdIns(4,5)P_2_ with a role in actin control is formed by the nanodomain-associated PI4P 5-kinase PIP5K2 (refs. ^23,26^). It is currently unclear how PIP5K2 and PtdIns(4,5)P_2_ interact with the actin cytoskeleton at the molecular level^23^ and by what intrinsic molecular mechanisms PtdIns(4,5)P_2_ formation by PIP5K2 is controlled.

Despite the importance of actin–membrane attachment for plant physiology, there is currently only limited information on how actin filaments intersect with the PM and what proteins or lipids mediate actin–PM contacts in plant cells. Plant genomes do not hold genes with similarity to those encoding proteins mediating actin–PM attachment in mammals or yeast^40–42^, such as the ERM protein family^43,44^ or Nck proteins^45^.

Candidate proteins for mediators of actin–PM-attachment include myosins of the plant-specific myosin class VIII^46^, which in *Arabidopsis* is represented by four isoforms, ATM1, VIII-A and the pollen-specific ATM2 and VIII-B^46^. Class VIII myosins display only minor motor activity compared with the better-characterized class XI myosins, also found in plants^46^. Myosins of classes VIII and XI have an overall similar domain architecture, with an amino-terminal region containing an SRC-homology 3 domain, an actin-binding motor domain, a lever domain containing calmodulin-binding IQ motifs, a coiled-coil region probably mediating interactions between myosin monomers and a carboxy-terminal region mediating target–cargo interactions^46^. Motor activity of myosins is thought to require the formation of myosin dimers^46,47^. However, not all myosins have been studied in this regard, and while ATM1 binds with high affinity to actin^47^, it has been proposed that class VIII myosins might not require motor activity for their physiological function at all^46,47^. Phenotypic defects of *Arabidopsis* mutants with genetic lesions in genes for class VIII myosins suggest their involvement in a number of physiological processes^48^. An *Arabidopsis* *atm1* mutant displays a smaller root apical meristem and reduced cell proliferation in a sugar-dependent manner^49^, and auxin signalling and transcriptional responses are dampened in roots of *atm1* mutants^49^. *Arabidopsis* ATM2 has been implicated in endocytosis^50^. While a role of class VIII myosins in cytoskeletal control and/or endocytosis has been proposed, in *Arabidopsis* the exact molecular function of class VIII myosins remains obscure. In tip-growing filament cells of the moss *Physcomitrium patens*, class VIII myosins mediate the interplay of actin and microtubules, thereby ensuring that actin polymerization and cell expansion are localized properly in the expanding apex of the moss filaments^51,52^. These important studies establish a role of class VIII myosins in the spatio-temporal coordination of plant cytoskeletal structures. There is currently little information on how class VIII myosins might interact with membranes.

According to the biochemical analysis of enriched recombinant myosin VIII fragments from *Arabidopsis*, the pollen-expressed class VIII myosin ATM2 binds anionic phospholipids^53^, and a C-terminal polybasic region of ATM2 is necessary and sufficient for lipid binding and PM association of ATM2 in pollen tubes^53^. Importantly, ATM2 and other class VIII myosins display features consistent with a role in defining the actin–PM interface by binding both actin and anionic lipids through separate N-terminal and C-terminal domains, respectively^46,47,53^. So far, it has not been demonstrated whether and how ATM2 mediates the attachment of actin to the PM and whether the action of ATM2 influences pollen tube expansion.

Here we show that the association of ATM2 with the PM exerts a stabilizing effect on actin dynamics in pollen tubes in vivo. ATM2 colocalizes and interacts with the PI4P 5-kinase PIP5K2 at the PM and promotes PIP5K2-mediated PtdIns(4,5)P_2_ formation in vivo and in vitro. On the basis of these observations, ATM2 may exert a dual effect by anchoring actin to PtdIns(4,5)P_2_ nanodomains while at the same time stimulating PtdIns(4,5)P_2_ formation, resulting in the self-reinforcing promotion of PtdIns(4,5)P_2_-dependent ATM2 recruitment and ROP activation to mediate actin stabilization at the actin–PM interface.

## Results

The control of actin–PM contacts by regulatory lipids involves the class VIII myosin ATM2 (ref. ^53^). To our knowledge, the physiological effects of ATM2 on either actin or the PM have not been explored. To characterize the function of ATM2 at the PM, we focused on its relations to PIP5K2 and PtdIns(4,5)P_2_ nanodomains controlling actin dynamics^23^.

### ATM2 and PIP5K2 may define actin–PM contacts

**Fig. 1:**
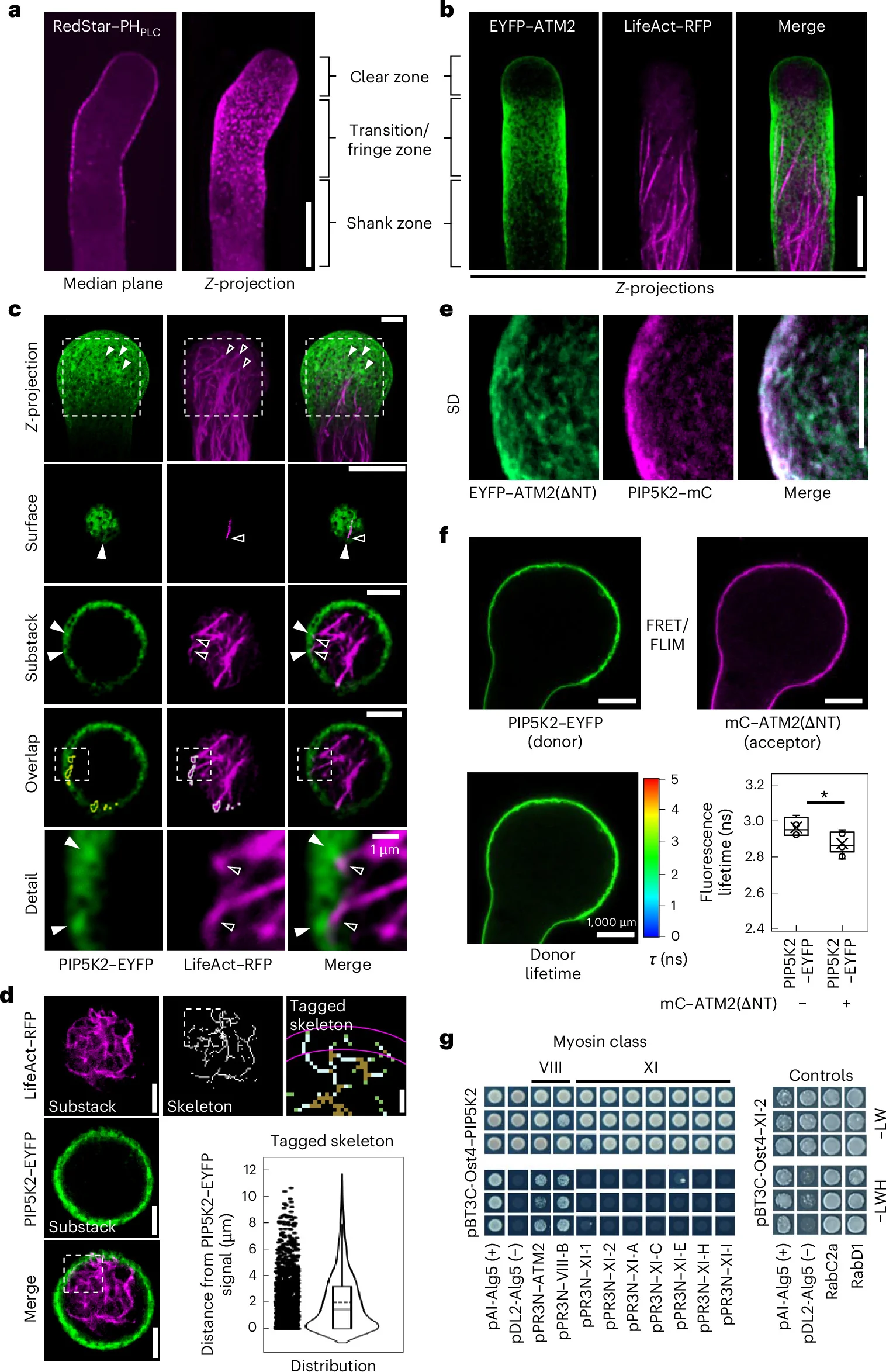
The PtdIns(4,5)P_2_ biosensor RedStar–PH_PLC_ and EYFP–ATM2 occupy overlapping subapical PM zones of tobacco pollen tubes, coinciding with actin–PM contacts. The subcellular distribution patterns of the PtdIns(4,5)P_2_ biosensor, RedStar–PH_PLC_; EYFP–ATM2; or the PtdIns(4,5)P_2_-forming enzyme, PIP5K2–EYFP, were analysed by SD or LSM upon coexpression in tobacco (*Nicotiana tabacum*) pollen tubes, relative to fluorescent markers for actin (LifeAct–RFP or mTalin–YFP). Expression was driven by the *pLat52* promoter. **a**,**b**, Fluorescence distribution of RedStar–PH_PLC_, EYFP–ATM2 and LifeAct–RFP, recorded by SD. The clear zone, the shank region and the transition zone are indicated. Panel **a** shows RedStar–PH_PLC_ (maximum-intensity *Z*-projection or median plane). Scale bar, 5 µm. Images are representative of at least three independent experiments. Panel **b** shows EYFP–ATM2 coexpressed with LifeAct–RFP. Scale bar, 5 µm. Images are representative of at least three independent experiments. **c**, Coexpression of *Arabidopsis* PIP5K2–EYFP and LifeAct–RFP. From top to bottom, the images show the maximum-intensity *Z*-projection from the pollen tube tip, substacks of the surface and tip periphery, the overlap regions (yellow/white) between LifeAct–RFP-marked actin filaments and PIP5K2–EYFP nanodomains as detected by Fiji, and a detail view (white box) highlighting actin attachment to PM nanodomains occupied by PIP5K2–EYFP (filled arrowheads indicate PIP5K2–EYFP; open arrowheads indicate LifeAct–RFP). Scale bars, 5 µm. Images are representative of at least three independent experiments. **d**, The intersection of LifeAct–RFP signals with PIP5K2–EYFP signals was analysed in more detail. LifeAct–RFP (magenta), PIP5K2–EYFP (green) and merged images are shown. Scale bars, 5 µm. The black-and-white image shows the skeleton of the LifeAct signal and the zoomed-in region of interest (dashed square). The LifeAct–RFP signal was skeletonized and tagged as follows: green for the end-points of actin filaments, light blue for slab pixels (pixels with exactly two neighbours) and brown for junction pixels (pixels with more than two neighbours). Magenta lines indicate the region occupied by PIP5K2–YFP signal. The dashed square in the ‘Skeleton’ panel is detailed as ‘Tagged skeleton’ (right). Several LifeAct–RFP-filament end-points overlap with the PIP5K2–EYFP signal or localize close to it. Scale bar (tagged skeleton), 1 µm. Bottom right: distribution of distances of LifeAct–RFP end-points from PIP5K2–EYFP signal at the PM. The data represent 1,202 data points collected from eight pollen tubes. Median distance, 1.75 μm. **e**, The PM patterns occupied by EYFP–ATM2(ΔNT) or PIP5K2–mC were analysed by SD in a coexpression experiment. Both fusions were expressed using the *pLat52* promoter. White indicates colocalization. The Pearson’s colocalization coefficient was determined as 0.43 ± 0.29 (mean ± standard deviation, *n* = 11). Scale bar, 5 µm. **f**, FRET/FLIM analysis of coexpressed PIP5K2–EYFP and mC–ATM2(ΔNT). The images show the fluorescence distributions of the PIP5K2–EYFP donor and the mC–ATM2(ΔNT) acceptor, and the donor lifetime (*τ*). Bottom right: fluorescence lifetime (*τ*) of the PIP5K2–EYFP FRET donor alone (left) and in the presence of the mC–ATM2(ΔNT) acceptor (right). Data are from four cells (donor alone) and nine cells (with acceptor). The centre lines show the medians, the Xs indicate the means, the box limits indicate the 25th and 75th percentiles and the whiskers extend to 1.5 times the interquartile range from the 25th and 75th percentiles. The asterisk indicates a significant change from the control according to a two-tailed Student’s *t*-test (*P = 0.023407895). **g**, Yeast-two-hybrid analysis using PIP5K2 as bait against fragments of selected myosin isoforms, including the pollen-specific class VIII myosins ATM2 and VIII-B and selected class XI myosins, as indicated. Left: interaction tests with PIP5K2 as a bait. Right: controls for class XI myosin interactions. Yeast growth on non-selective –LW medium confirmed correct transformation, while growth on selective –LWH medium indicated positive interactions. The results are representative of three independent biological replicates, each performed in triplicate.

In pollen tubes of various plant species, the extreme apical cytosol (the ‘clear zone’) contains no larger organelles and is populated by secretory vesicles driving polarized cell expansion. The extent of the clear zone is defined by a fine network of dynamic peripheral actin filaments thought to guide vesicles to their respective sites of membrane insertion, or to remove endocytic vesicles^1^. The PM of the endocytic zone of tobacco pollen tubes^1,54^ is occupied by membrane nanodomains decorated by a RedStar–PH_PLC_ marker^38^ indicating the presence of PtdIns(4,5)P_2_ (Fig. 1a), as imaged by spinning disc microscopy (SD). A fluorescent marker for the class VIII myosin ATM2, EYFP–ATM2, imaged by SD displayed a structurally inhomogeneous pattern of PM association (Fig. 1b) and decorated a PM zone at the transition between the apical clear zone and the shank region of tobacco pollen tubes, where dynamic actin filaments are labelled by a coexpressed LifeAct–RFP marker^23,55^ (Fig. 1b). The PtdIns(4,5)P_2_ biosensor and the EYFP–ATM2 marker thus occupy largely overlapping subapical PM regions in growing tobacco pollen tubes.

**Extended Data Fig. 1:**
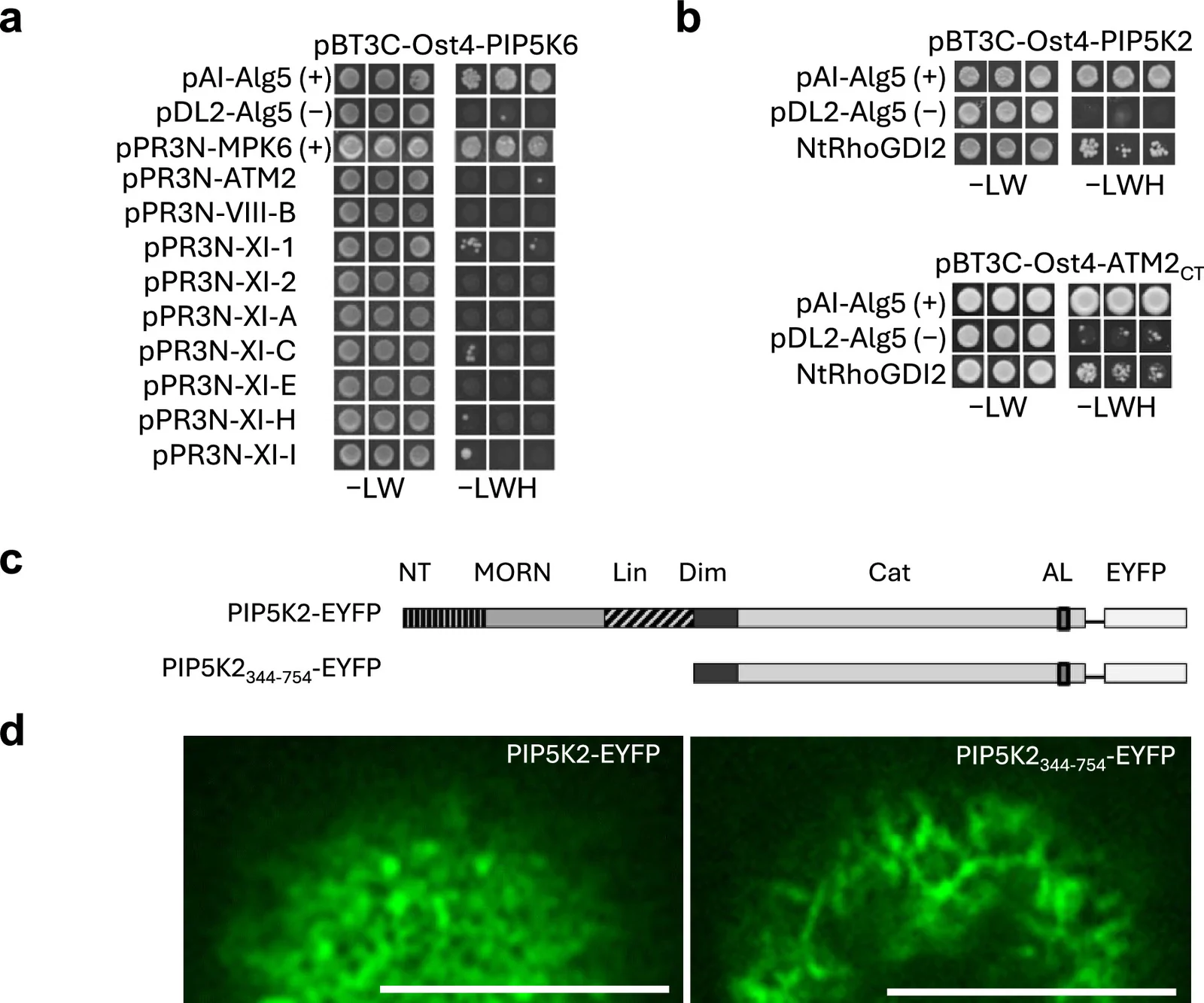
Association of PIP5K2 with factors modulating the actin cytoskeleton. (**a**) Yeast-two-hybrid analysis using PIP5K6 as a bait against selected myosin isoforms, including the pollen-specific class VIII myosins ATM2 or VIIIB, and various class XI myosins, as indicated. (Please see positive interaction of PIP5K2 with class VIII myosins in Fig. 1g.) Interaction with Arabidopsis mitogen-activated protein kinase 6 (MPK6) serves as a positive control. Yeast growth on non-selective –LW medium confirmed correct transformation, while growth on selective –LWH medium indicated positive interactions. Results are representative for three independent biological replicates, each performed in triplicates. (**b**) Yeast-two-hybrid analysis using PIP5K2 or a C-terminal ATM2-fragment, respectively, as bait proteins against NtRhoGDI2. Top, interaction tests with PIP5K2 as a bait; Bottom, Interactions tests with ATM2 as a bait. Yeast growth on non-selective –LW medium confirmed correct transformation, while growth on selective –LWH medium indicated positive interactions. Results are representative for three independent biological replicates, each performed in triplicates. (**c**, **d**) Relaxed localization of PIP5K2_344-754_-EYFP in a PM-peripheral network-like pattern in tobacco pollen tubes. Expression was driven by a *Lat52* promoter. The fluorescence distribution of AtPIP5K2-EYFP or the N-terminally truncated AtPIP5K2_344-754_-EYFP was analyzed by spinning disc microscopy upon transient expression in tobacco pollen tubes. (**c**) Representation of the expressed EYFP fusion proteins. NT, N-terminal domain; MORN, membrane occupation and recognition nexus repeat domain; Lin, hypervariable linker region; Dim, dimerization domain; Cat, catalytic domain; AL, activation loop. (**d**) PIP5K2-EYFP displays dot-like PM association resembling nanodomains in the apex of a tobacco pollen tube (left). AtPIP5K2_344-754_-EYFP decorates a PM-proximal filamentous network-pattern, possibly representing the cortical actin network. Images are representative for three independent experiments. Scale, 5 µm.

PtdIns(4,5)P_2_ with a role in the control of actin dynamics is formed by the PI4P 5-kinase PIP5K2 which also resides in membrane nanodomains and when overexpressed causes pollen tube tip swelling due to a stabilizing effect on actin^23^. PM nanodomains decorated by PIP5K2–EYFP intersected in pollen tubes with PM-oriented actin filaments labelled by the actin marker LifeAct–RFP (Fig. 1c). PM-proximal actin filaments, as they appear in the surface scan (second row), end at nanodomains labelled by PIP5K2–EYFP. Computer-aided identification of regions of overlap of the fluorescent markers in subsets of confocal imaging stacks also indicated PM nanodomains with a diameter <1 µm as possible contact sites of actin filaments decorated by LifeAct–RFP with PIP5K2–EYFP at the PM (Fig. 1c, overlap and detail). The images shown in Fig. 1c represent substacks and not individual confocal sections to capture the three-dimensional network of actin filaments. The incidence of LifeAct–RFP signals increased near PIP5K2–EYFP signals (Fig. 1d), suggesting the association of PM-proximal actin with PIP5K2–EYFP nanodomains. To assess whether ATM2 might mediate actin–PM contacts at PIP5K2–EYFP nanodomains, we tested for colocalization of PIP5K2–EYFP and mCherry (mC)–ATM2 (Fig. 1e). No pollen tubes coexpressing PIP5K2–EYFP with full-length ATM2 were recovered, but coexpression was achieved using an ATM2 variant lacking part of its N terminus (mC–ATM2(ΔNT)). mC–ATM2(ΔNT) colocalized with PIP5K2–EYFP at the PM in an inhomogeneous distribution pattern, imaged by SD (Fig. 1e). Upon coexpression of mC–ATM2(ΔNT) and PIP5K2–EYFP, fluorescence resonance electron transfer/fluorescence lifetime imaging (FRET/FLIM) analysis indicated a slight but significant reduction in PIP5K2–EYFP donor fluorescence lifetime in the presence of mC–ATM2(ΔNT), compared with that of PIP5K2–EYFP alone (Fig. 1f), consistent with an in vivo interaction of mC–ATM2(ΔNT) and PIP5K2–EYFP in planta. The raw data of the FRET/FLIM analysis can be found in Supplementary Table 1. The interaction of a C-terminal ATM2 fragment with PIP5K2 was confirmed by split-ubiquitin-based yeast-two-hybrid analysis (Fig. 1g). The interaction seems specific to class VIII myosins, as both pollen-expressed class VIII myosins, ATM2 and VIII-B, tested positive, in contrast to seven class XI myosins tested (Fig. 1g). Known interactions of the class XI myosin XI-2 (ref. ^56^) served as positive controls. Moreover, the non-nanodomain-associating PI4P 5-kinase PIP5K6 displayed no interaction with any of the myosins tested in the yeast-two-hybrid analysis (Extended Data Fig. 1a). Both PIP5K2 and a C-terminal ATM2 fragment interacted in the yeast-two-hybrid analysis with another regulator of PM-proximal actin, the GDI *Nt*RhoGDI2 (Extended Data Fig. 1b), suggesting their action in a common pathway to control actin. An EYFP-tagged N-terminal truncation of PIP5K2 located in a PM-proximal network-like pattern (Extended Data Fig. 1c,d) differing from the distribution of EYFP-tagged full-length PIP5K2 (Fig. 1c) and indicating that PIP5K2 can bind filamentous structures possibly representing membrane-peripheral actin.

### ATM2 influences actin dynamics at the PM

**Fig. 2:**
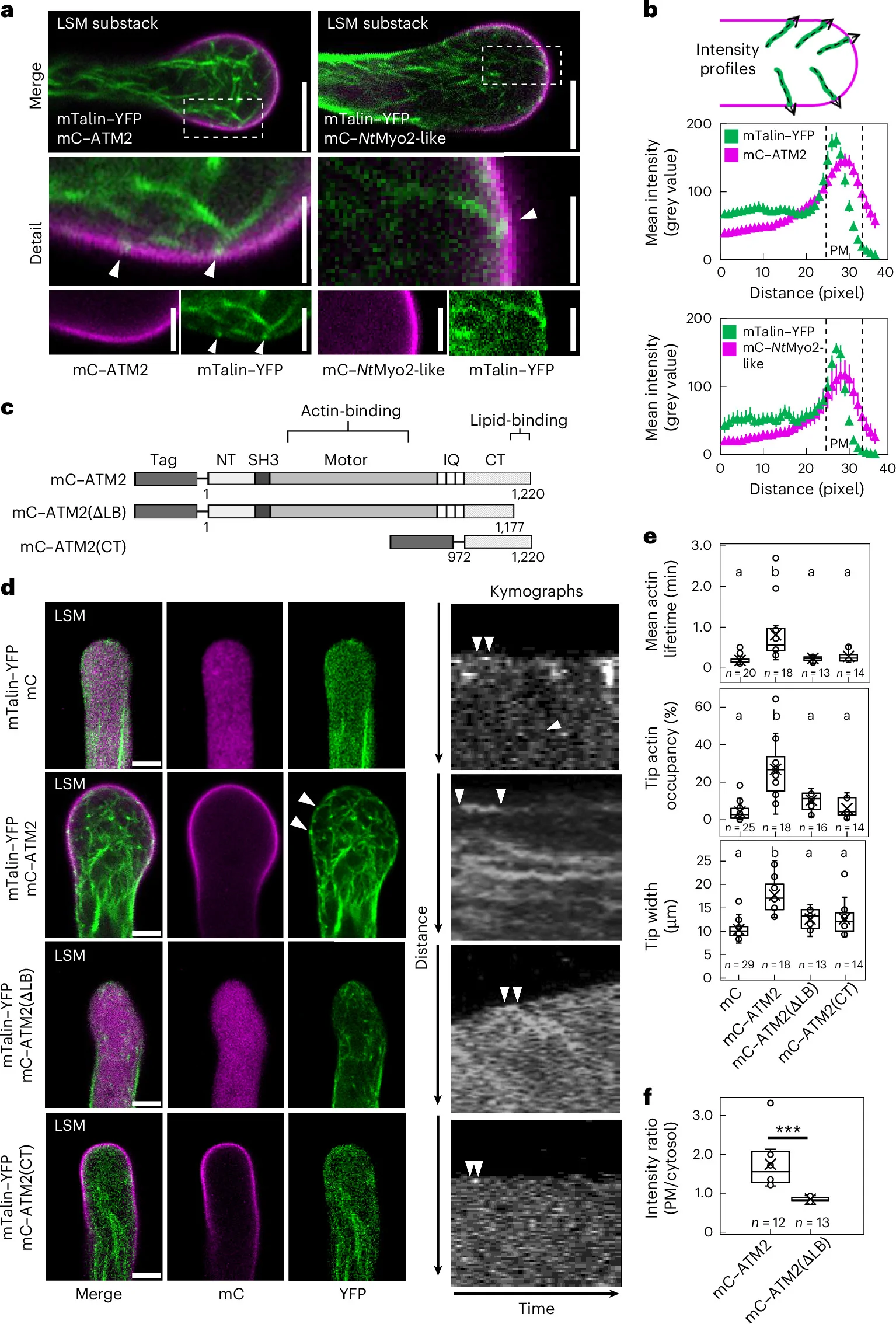
Overexpression of ATM2 reduces actin dynamics and induces tip swelling morphology in pollen tube cells. The effects of expressing class VIII myosins on the distribution and motility of the actin cytoskeleton were monitored by LSM in tobacco (*N. tabacum*) pollen tubes. The expression of all constructs was driven by the *pLat52* promoter. **a**, Coexpression of mC–ATM2 (left) or its tobacco homologue, mC–*Nt*Myo2-like (right), with mTalin–YFP, imaged by LSM. Top: merged maximum-intensity *Z*-projections. Bottom: details (white boxes) of locally increased mTalin–YFP signals at the PM (arrowheads). Scale bars, 10 µm. Images are representative of at least three independent experiments. **b**, Quantification of mTalin–YFP fluorescence along actin filaments at the intersection with the PM upon coexpression of mC–ATM2 or mC–*Nt*Myo2-like. Top: illustration of the acquisition of intensity profiles along PM-intersecting filaments (arrows). Middle: mean intensity profiles for mTalin–YFP (green) and mC–ATM2 (magenta). Bottom: mean intensity profiles for mTalin–YFP (green) and mC–*Nt*Myo2-like (magenta). The data points indicate means ± standard deviations from 22 traces from six cells (mC–ATM2) or 32 traces from seven cells (mC–*Nt*Myo2-like). The region of PM intersection is indicated. **c**–**f**, The distribution and motility of actin were monitored by LSM in tobacco (*N. tabacum*) pollen tubes coexpressing the actin marker mTalin–YFP with either an mC-control or mC-tagged variants of ATM2, including full-length ATM2 (mC–ATM2), an ATM2 variant lacking the C-terminal lipid-binding region (mC–ATM2(ΔLB)) or an ATM2 variant lacking the N-terminal actin-binding and protein-interaction regions (mC–ATM2(CT)). A schematic representation of the mC fusions of ATM2 variants used is shown in **c**. The numbers indicate ATM2 residue positions. Motor, motor-ATPase domain; SH3, SRC-homology 3-domain. To capture actin dynamics, *Z*-stacks (2-µm steps) were acquired in 10-s intervals over 5 min (**d**). Left: representative maximum-intensity projections of merged mTalin–YFP and mC fluorescence channels. Middle and right: separate *Z*-projection images of the mC channel (control and ATM2 variants) or the YFP channel (mTalin). The arrowheads indicate locally increased mTalin–YFP signals at the PM. Scale bars, 5 µm. Kymographs extracted from the pollen tube tip regions are shown on the far right. Stabilized actin filaments are represented by horizontal lines. The arrowheads indicate individual mTalin traces. As shown in **e**, pollen tube cells coexpressing the actin marker mTalin–YFP with an mC control, mC–ATM2, mC–ATM2(ΔLB) or mC–ATM2(CT) were analysed for kymographic mean actin lifetime (top), actin occupancy in the cell apex (middle) and tip swelling (bottom). The mean actin lifetime of mTalin-labelled actin filaments was calculated from kymographs. The data represent observations from 20 cells (mC), 18 cells (mC–ATM2), 13 cells (mC–ATM2(ΔLB)) or 14 cells (mC–ATM2(CT)). The tip actin occupancy was quantified as the relative area covered by mTalin–YFP signal at the pollen tube tip. The data represent observations from 25 cells (mC), 18 cells (mC–ATM2), 16 cells (mC–ATM2(ΔLB)) or 14 cells (mC–ATM2(CT)). Pollen tube tip width was determined using Fiji. The data represent observations from 29 cells (mC), 18 cells (mC–ATM2), 13 cells (mC–ATM2(ΔLB)) or 14 cells (mC–ATM2(CT)) from three or four independent biological experiments. Statistical differences were assessed by one-way analysis of variance (ANOVA) followed by Tukey’s post-hoc test; letters denote categories of values exhibiting statistically significant differences (for mean actin lifetime, *P* < 1.559 × 10^−6^; for tip actin occupancy, *P* = 3.222 × 10^−10^; for tip width, *P* = 3.407 × 10^−11^). Panel **f** shows the degree of PM association of mC–ATM2 or the lipid-binding-deficient mC–ATM2(ΔLB) variant according to the fluorescence intensity ratio of mC signals at the PM versus cytosol. The data are from three independent experiments and represent observations from 12 cells (mC–ATM2) or 13 cells (mC–ATM2(ΔLB)). The asterisks indicate a significant change from the control according to a two-tailed Student’s *t*-test (***P = 2.47 × 10^−5^). In **e** and **f**, the centre lines show the medians, the Xs indicate the means, the box limits indicate the 25th and 75th percentiles and the whiskers extend to 1.5 times the interquartile range from the 25th and 75th percentiles. Data points outside this range are plotted as individual outliers.

**Extended Data Fig. 2:**
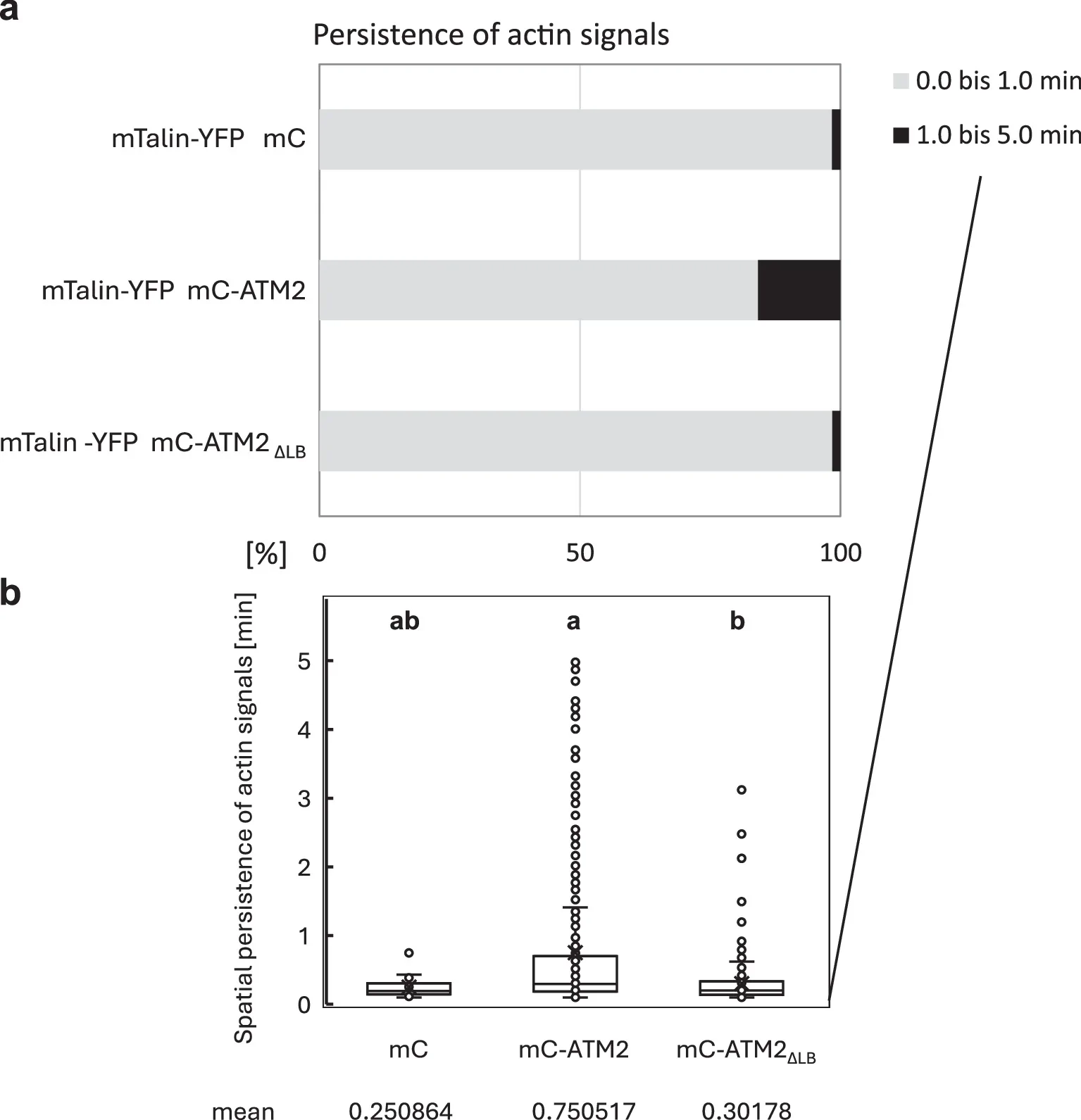
Detailed analysis of mean actin lifetimes in pollen tube cells expressing an mC-control, mC-ATM2, or mC-ATM2_ΔLB_. Data from the analysis of mean lifetimes of mTalin-labeled actin filaments were calculated from kymographs and analyzed for the proportion of filaments displaying low-mobility. (**a**) Proportion of mTalin-YFP signals displaying a spatial persistence of 0 min - 1 min (grey, high mobility) or of 1 min - 5 min (black, low mobility). (**b**) Exact numbers underlying the representation in (**a**). Data represent observations from 20 cells (mC); 18 cells (mC-ATM2); 13 cells (mC-ATM2_ΔLB_), respectively from 3-4 independent biological experiments. Center lines show the medians, the x indicate the means. Box limits indicate the 25th and 75th percentiles. Whiskers extend 1.5 times the interquartile range from the 25th and 75th percentiles. Data points outside this range are plotted as individual outliers.

**Extended Data Fig. 3:**
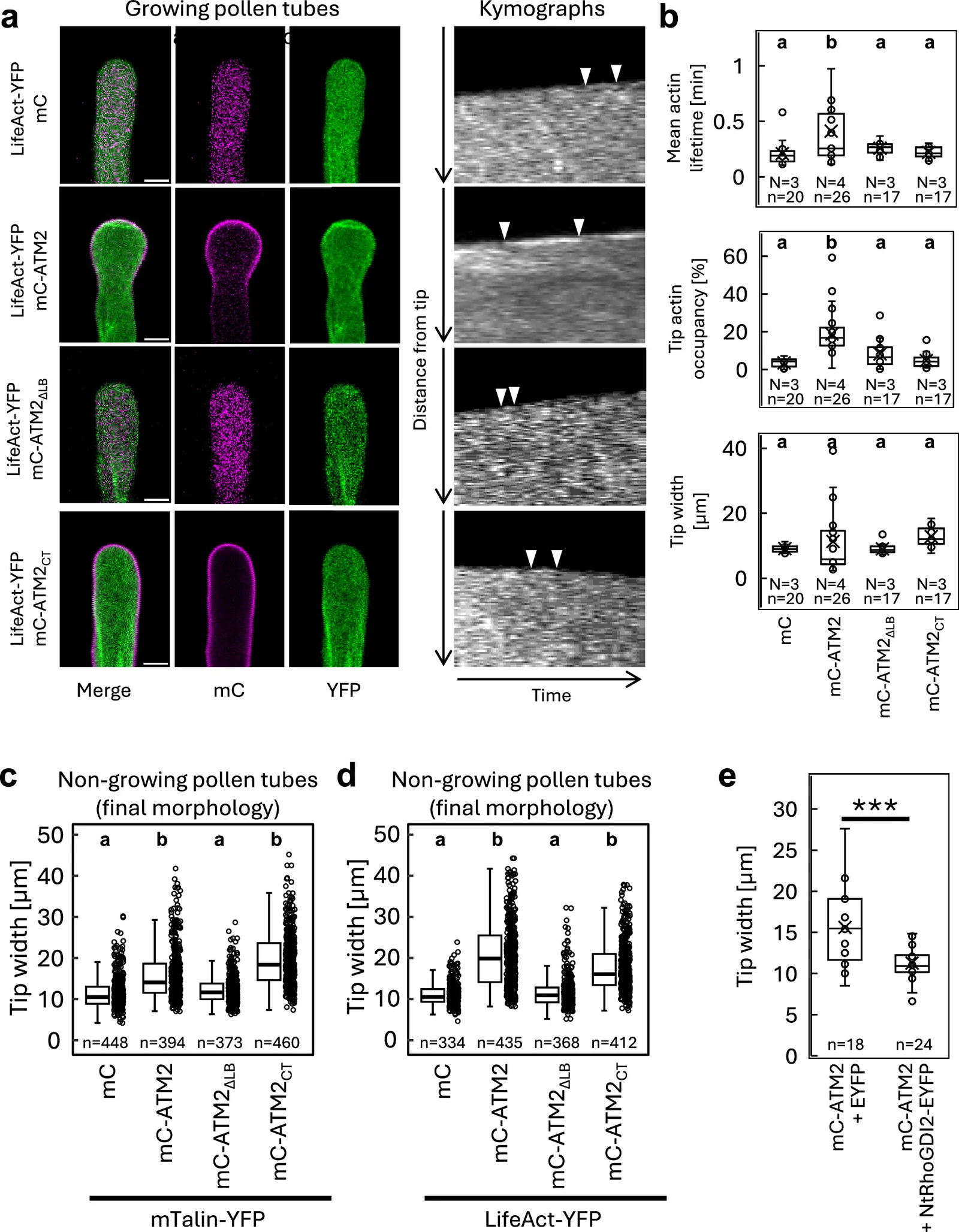
Overexpression of ATM2 reduces actin dynamics and induces tip swelling morphology in pollen tube cells (based on LifeAct-YFP). The effects of expressing class VIII-myosins on distribution and motility of the actin cytoskeleton were monitored by laser-scanning confocal microscopy (LSM) in tobacco (*N. tabacum*) pollen tubes. Expression of all constructs was driven by a *pLat52* promoter. (**a**, **b**) Distribution and motility of actin were monitored by LSM in tobacco (*N. tabacum*) pollen tubes coexpressing the actin marker, LifeAct-YFP, with either an mC-control, or with mC-tagged variants of ATM2, including full length ATM2 (mC-ATM2), an ATM2 variant lacking the C-terminal lipid-binding region (mC-ATM2_ΔLB_), or an ATM2 variant lacking N-terminal actin-binding and protein-interaction regions (mC-ATM2). (**a**) To capture actin dynamics, Z-stacks (2 µm steps) were acquired in 10 s intervals over 5 min. Left column, representative maximum intensity projections of merged LifeAct-YFP and mC fluorescence channels. Mid and right column, separate Z-projection images of the YFP channel (LifeAct) or the mC channel (control and ATM2 variants), respectively. Scale, 10 µm. Right panels, Kymographs extracted from the pollen tube tip regions. Stabilized actin filaments are represented by horizontal lines. Arrowheads indicate individual LifeAct-YFP traces. (**b**) Pollen tube cells coexpressing LifeAct-YFP with an mC-control, mC-ATM2, with mC-ATM2_ΔLB_, or with mC-ATM2 were analyzed for mean actin lifetime (top), actin occupancy in the cell apex (mid), or tip swelling (bottom). The mean actin lifetime (in min) of mTalin-labeled actin filaments was calculated from kymographs. The tip actin occupancy was quantified as the relative area covered by mTalin-YFP signal at the pollen tube tip. Pollen tube tip width (in µm) was determined from brightfield images using Fiji. Data represent observations from 20 cells (mC); 26 cells (mC-ATM2); 17 cells (mC-ATM2_ΔLB_); or 17 cells (mC-ATM2), respectively. Data represent 3-4 independent biological experiments. Statistical differences were assessed by one-way ANOVA followed by Tukey’s post-hoc test; letters denote categories of values exhibiting statistical significant differences (mean actin lifetime, p = 2.746 × 10^−8^; tip actin occupancy, p = 0.0007442; tip width, p = 0.1695). (**c**, **d**) Pollen tube tip swelling was analyzed upon cessation of growth 20 h after transformation in cells coexpressing either YFP-mTalin or LifeAct-YFP with an mC-control, mC-ATM2, with mC-ATM2_ΔLB_, or with mC-ATM2, as indicated. (**c**) Data for YFP-mTalin. Data are from three independent experiments representing 448 cells (mC-control), 394 cells (mC-ATM2), 372 cells (mC-ATM2_ΔLB_) and 454 cells (mC-ATM2), respectively. Statistical differences were assessed by one-way ANOVA followed by Tukey’s post-hoc test; letters denote categories of values exhibiting statistical significant differences (p = 1.081249 × 10^−114^). (**d**) Data for LifeAct-YFP. Data are from three independent experiments representing 334 cells (mC-control), 435 cells (mC-ATM2), 368 cells (mC-ATM2_ΔLB_) and 414 cells (mC-ATM2), respectively. Statistical differences were assessed by one-way ANOVA followed by Tukey’s post-hoc test; letters denote categories of values exhibiting statistical significant differences (p = 2.247899 × 10^−136^). (**e**) To determine whether pollen tube tip swelling induced by expressing was mC-ATM2 involved the ROP-pathway, pollen tube tip width was determined for cells coexpressing either mC-ATM2 with an EYFP control (left bar) or mC-ATM2 with NtRhoGDI2-EYFP (right bar). Data are from two independent experiments representing 18 cells (EYFP) and 24 cells (NtRhoGDI2-EYFP), respectively. Asterisks indicate a significant change from the control according to a Student’s T-test (***, p = 0.000316). (**b** - **e**) Center lines show the medians, the x indicate the means. Box limits indicate the 25th and 75th percentiles. Whiskers extend 1.5 times the interquartile range from the 25th and 75th percentiles. Data points outside this range are plotted as individual outliers.

When mC–ATM2 was coexpressed with an mTalin–YFP marker for actin^57^ in tobacco pollen tubes, we observed enhanced actin signals at the membrane-proximal ends of actin filaments intersecting with the PM (Fig. 2a, arrowheads). The effect was observed upon expression of an mC fusion of *Arabidopsis* ATM2 (Fig. 2a, left) as well as a fusion of tobacco ATM2 (*Nt*Myo2-like; Fig. 2a, right). Actin filaments were only observed in the clear zone upon overexpression of mC–ATM2 or mC–*Nt*Myo2-like, and no enhanced actin–PM contacts were evident upon expression of the mTalin–YFP marker alone (Fig. 2d, top). The laser scanning confocal microscopy (LSM) images in Fig. 2a represent substacks and not individual confocal sections to capture the three-dimensional network of actin filaments. Quantification of the mTalin–YFP signal across the filament–PM intersections indicates increased mTalin–YFP fluorescence immediately inwards of the PM-associated mC–ATM2 fluorescence (Fig. 2b), leading us to speculate on a possible stabilizing effect of ATM2 on actin at the PM–actin interface. To determine whether the overexpression of ATM2 variants would mediate a stabilized actin cytoskeleton, actin dynamics and cell morphologies were assessed by LSM in pollen tubes overexpressing mC-tagged variants of ATM2 (Fig. 2c,d), including full-length ATM2 (mC–ATM2), a variant lacking the C-terminal lipid-binding domain (mC–ATM2(ΔLB)) and a C-terminal fragment of ATM2 lacking the actin-binding motor domain (mC–ATM2(CT)), together with an actin marker, mTalin–YFP^23,57^. Figure 2c gives an overview of the ATM2 variants used. In control pollen tubes coexpressing an mC protein and mTalin–YFP, actin was dynamic according to kymograph analysis (Fig. 2d, top right; the arrowheads indicate the duration of an individual actin trace) and predominantly occupied the shank region of the control cells (Fig. 2d, top), in a pattern resembling previous observations^23^. Moreover, the control cells displayed normal pollen tube cell morphology with tip diameters of approximately 10 µm (Fig. 2d). By contrast, the expression of mC–ATM2 resulted in reduced actin dynamics (indicated by kymographic mean actin lifetime), enhanced actin occupancy in the pollen tube tip region and substantial pollen tube tip swelling (Fig. 2d). Quantitative analysis of mean actin lifetime, tip actin occupancy and pollen tube tip diameter (Fig. 2e) indicates statistically significant effects. Deletion of the C-terminal lipid-binding region of ATM2 in the mC–ATM2(ΔLB) variant abolished PM association (Fig. 2d,f), and expression of mC–ATM2(ΔLB) did not result in altered actin dynamics, altered actin occupancy or changed cell morphology (Fig. 2d,e). Similarly, the expression of mC–ATM2(CT) had no effect on actin dynamics, actin occupancy or pollen tube tip width, despite the retained PM association of this C-terminal ATM2 fragment (Fig. 2d,e). Further quantitative analysis of the spatial persistence of actin signals verified enhanced persistence of actin filaments upon expression of mC–ATM2, compared with the mC controls or with tubes expressing mC–ATM2(ΔLB) (Extended Data Fig. 2). To rule out a marker effect of using mTalin^58^, we also assessed the effects of the three ATM2 variants (Fig. 2c) on actin dynamics using the alternative actin marker LifeAct–YFP, confirming the results (Extended Data Fig. 3a,b). The effects of the ATM2 variants on tip swelling were additionally assessed by determining terminal cell morphologies in non-growing pollen tubes expressing either mTalin–YFP or LifeAct–YFP (Extended Data Fig. 3c,d). In this experimental set-up, the C-terminal ATM2(CT) fragment also induced moderate tip swelling. Moreover, we observed that apical tip swelling upon expressing full-length mC–ATM2 was abolished in the presence of the coexpressed GDI, *Nt*RhoGDI2–EYFP (Extended Data Fig. 3e), indicating that ATM2-induced tip swelling was mediated by ROP/GDI-dependent regulation^24,32^ that is also subject to control by PIP5K2 (ref. ^23^).

**Extended Data Fig. 4:**
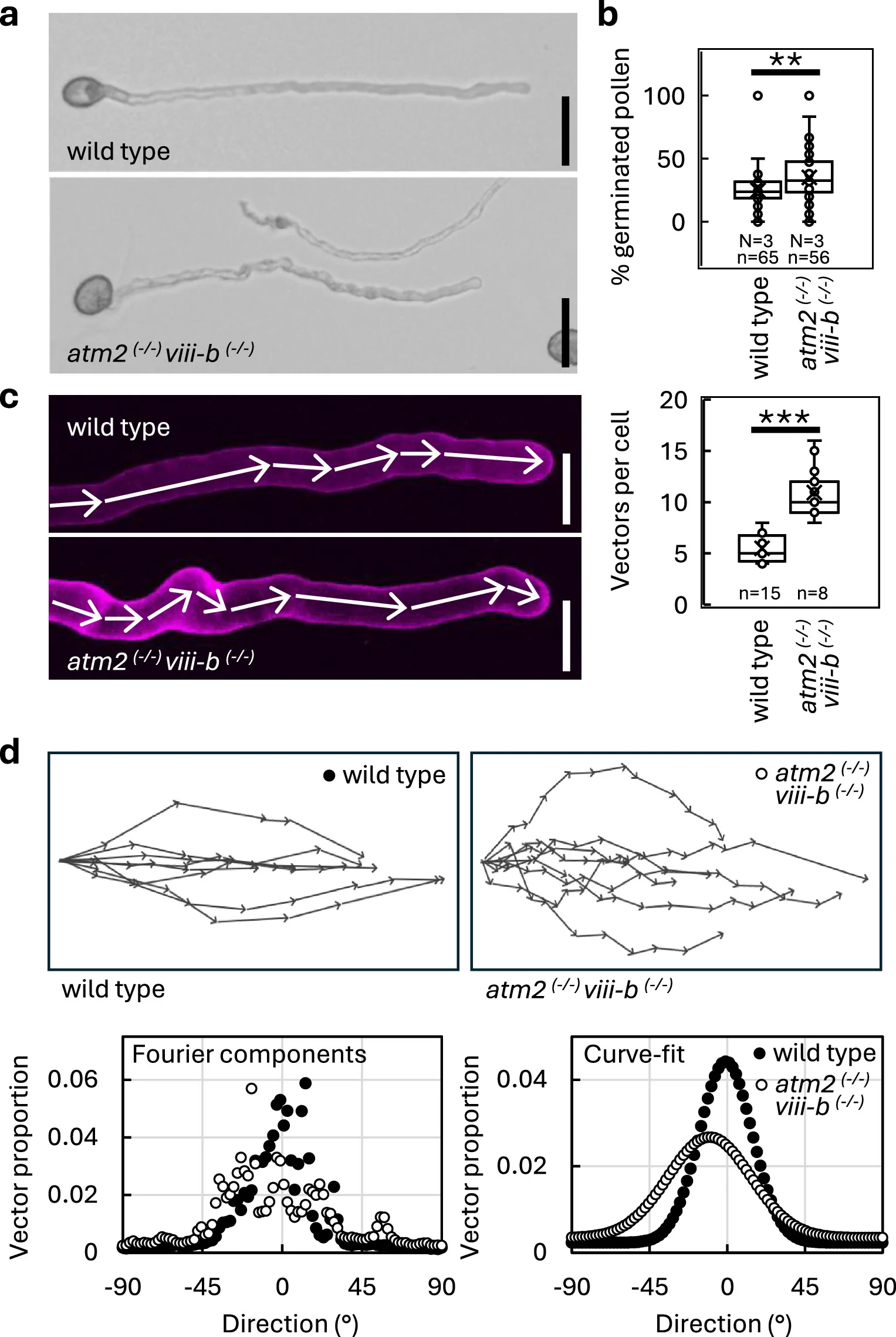
Arabidopsis mutants with reduced expression of the *ATM2* and *VIII-B* genes display defects in pollen germination and pollen tube growth. (**a**) Pollen of double-homozygous *atm2 viii-b* mutants were collected and germinated on pollen germination medium. The microscopic appearance indicated a growth defect. (**b**) Quantification of pollen germination. Data are from three independent experiments and represent 3272 (wild type) and 1485 (*atm2 viii-b*) scored pollen, respectively. (**c**) The pollen tube growth defect of the *atm2 viii-b* mutant is characterized by frequent changes in the growth direction. Left, pollen tube morphology for wild type (upper panel) or the *atm2 viii-b* mutant (lower panel). To assess the aberrant growth, flattened images of three-dimensional confocal scans were analyzed by adding vectors according to the stages of directional cell expansion, as indicated by the arrows. Right panel, Mean number of vectors needed to describe wild type pollen tubes (left bar) or *atm2 viii-b* mutant pollen tubes (right bar). (**b**, **c**) Center lines show the medians, the x indicate the means. Box limits indicate the 25th and 75th percentiles. Whiskers extend 1.5 times the interquartile range from the 25th and 75th percentiles. Data points outside this range are plotted as individual outliers. The asterisks indicate a significant change from the wild type control according to a 2-tailed Student’s T-test (% germinated pollen, **, p = 0.00449; Vectors per cell, ***, p = 4.71867 × 10^−6^). (**d**) The sum of all arrows describing one pollen tube was termed a ‘vector map’. Representative vector maps for wild type pollen tubes (upper left) or *atm2 viii-b* mutant pollen tubes (upper right). Vector maps were analyzed using the ImageJ directionality plugin, providing Fourier components indicating the degree of deviation from straight growth (0°). Lower left, Raw Fourier component output; lower right, Ideal curve fit derived from the raw Fourier component output.

These observations are supported by the analysis of the germination and growth of pollen tubes of an *Arabidopsis* line carrying homozygous transfer-DNA insertions in the *ATM2* (SALK_052429) and *VIII-B* (SALK_009323) genes, resulting in reduced transcript abundance^59^ (Extended Data Fig. 4). Pollen from *atm2* *viii-b* mutants germinated better than that of wild-type controls (Extended Data Fig. 4a,b), in line with previous reports of enhanced pollen germination in *Arabidopsis* mutants with a destabilized actin cytoskeleton^60–62^. The *atm2* *viii-b* mutant pollen displayed a noticeable growth defect characterized by aberrant directional cell expansion (Extended Data Fig. 4c,d). The growth of wild-type controls and the *atm2* *viii-b* mutant pollen was assessed by generating growth vector maps based on flattened images of 3D scans of growing pollen tubes stained with propidium iodide (Extended Data Fig. 4c,d). The *atm2* *viii-b* mutant pollen tubes required a significantly higher number of vectors to describe their directional growth (Extended Data Fig. 4c), and the required vectors displayed a significantly larger range of angles deviating from the main direction of growth than was observed in wild-type pollen, as analysed using the ImageJ directionality plugin^63^ (Extended Data Fig. 4d).

The combined data from the gain-of-function and the loss-of-function experiments indicate an unexpected stabilizing effect of ATM2 on actin, which required membrane association of ATM2 via its C-terminal lipid-binding region.

### ATM2 displays no lipid-binding preference in vitro

**Fig. 3:**
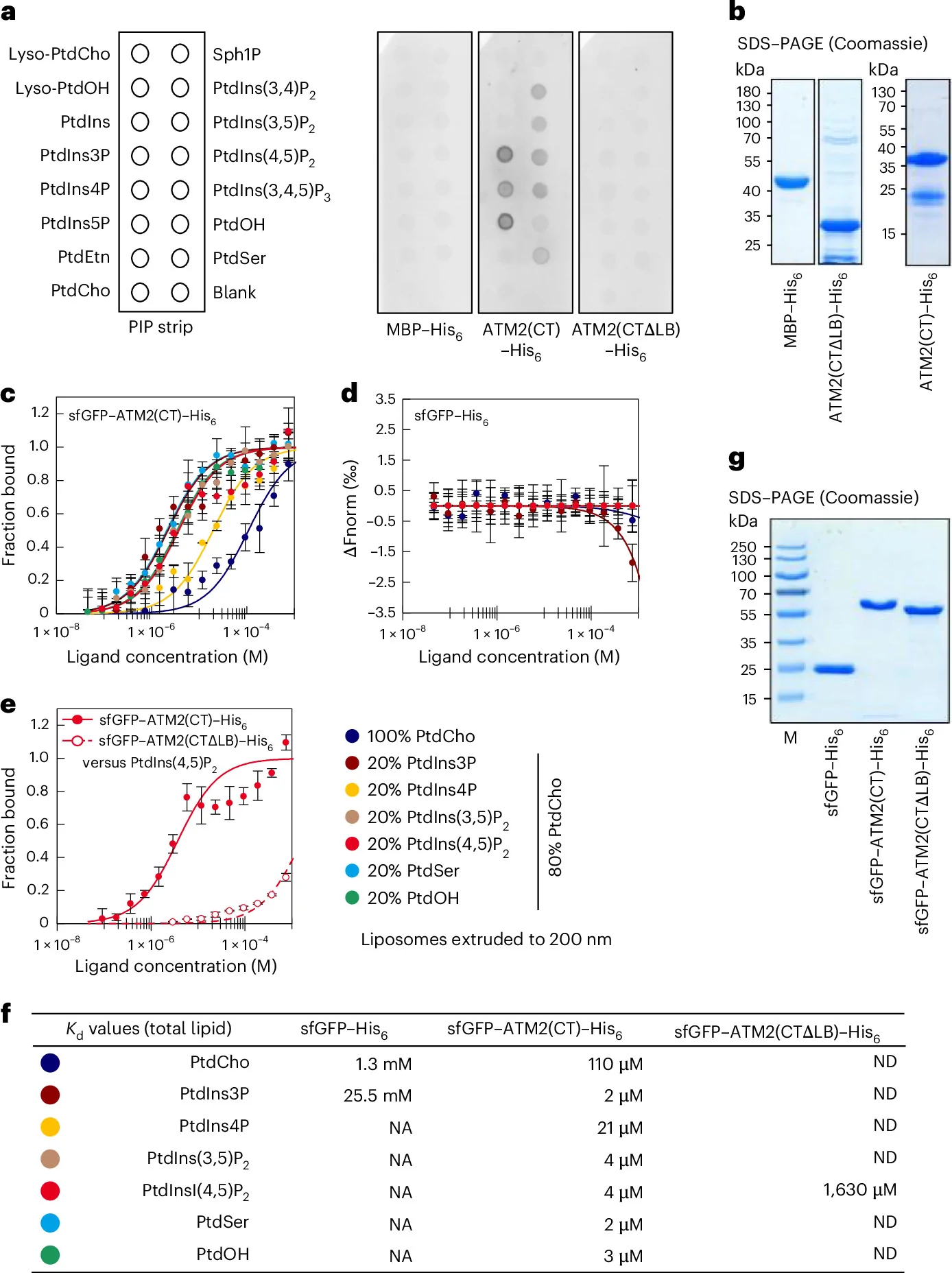
Lipid-binding features of recombinant C-terminal ATM2 fragments. Lipid binding of the polybasic C-terminal region of ATM2 was revisited by biochemical analysis, including quantitative MST analysis. ATM2 variants representing the C-terminal region of ATM2 (ATM2(CT), residues 972–1220) or the derived lipid-binding-deficient variant ATM2(CTΔLB) (residues 972–1177) were recombinantly expressed as fusions to C-terminal His_6_ tags, enriched and used for in vitro protein–lipid interaction tests. For MST analysis, proteins were additionally fused to N-terminal sfGFP tags. Enriched MBP–His_6_ and sfGFP alone were used as negative controls. **a**, Protein–lipid overlay assay for binding of an MBP–His_6_ control, ATM2(CT)–His_6_ or ATM2(CTΔLB)–His_6_ to phospholipids (10 nmol) using commercial PIP strips (Echelon Biosciences Inc.). Images of the developed protein–lipid overlay blots are representative of three experiments. **b**, Coomassie-blue-stained SDS–PAGE gel of enriched recombinant His_6_ fusions used for lipid-binding tests by lipid overlay assays. The deduced fragment masses are 43.3 kDa for MBP–His_6_, 29.5 kDa for ATM2(CT)–His_6_ and 24.5 kDa for ATM2(CTΔLB)–His_6_. **c**–**e**, MST analysis of the binding of enriched sfGFP–ATM2(CT)–His_6_ to DOPC liposomes containing 20 mol% of various anionic phospholipids. In **c**, binding curves depict the fraction of bound protein plotted versus the logarithmic ligand concentration. Proteins were incubated at an approximate concentration of 40 nM with liposomes serially diluted over 16 steps from 3 mM to 45.78 nM total lipid. The MST-on time was 2.5 s; data were fitted with the KD-fit model. Each data point represents the mean of four independent measurements; the error bars indicate standard deviations. Panel **d** shows MST control measurements with enriched sfGFP–His_6_ and DOPC liposomes containing 20 mol% of the respective anionic lipids, as in (**c**). The ΔFnorm value reflects the change in the local concentration or hydration shell of sfGFP as a function of temperature and ligand concentration. Flat traces across all titration points demonstrate that the migration behavior of sfGFP remained unaltered, confirming no interaction with any of the liposomes. Please note that all liposome regimes tested in (**c**) were also tested in (**d**), and that the traces all overlap. As no binding was detected for sfGFP–His_6_, the data are represented as Fnorm in ‰ against the logarithmic ligand concentration. Each data point represents the mean of four independent measurements; the error bars indicate standard deviations. Panel **e** shows MST analysis comparing the binding of enriched ATM2 (solid line) and ATM2(CTΔLB)–His_6_ (dashed line) to DOPC liposomes containing 20 mol% PtdIns(4,5)P_2_. MST-on time was 2.5 s, and the data were fitted using the KD-fit model. Each data point represents the mean of four independent measurements; the error bars indicate standard deviations. ATM2 data are the same as in **c**. **f**, *K*_d_ values representing binding affinities for measurements presented in **c** and **e**. Signal-to-noise ratios were above 5, reflecting reliable binding measurements. Relative affinities are given for binding to PtdIns(4,5)P_2_-containing liposomes as a reference (right column). NA, not applicable; ND, not determined. **g**, Coomassie-blue-stained SDS–PAGE gel of enriched recombinant sfGFP fusions used for lipid-binding tests by MST. The deduced fragment masses are 28.5 kDa for sfGFP–His_6_, 57.6 kDa for sfGFP–ATM2–His_6_ and 52.4 kDa for sfGFP–ATM2(CTΔLB)–His_6_. M, protein size marker, as indicated. Source data

Lipid-binding characteristics were revisited for two ATM2-derived protein fragments, ATM2(CT) and ATM2(CTΔLB) (additionally lacking the 43 C-terminal residues representing the polybasic lipid-binding region) (Fig. 3). In previous analyses, a recombinant C-terminal fragment of ATM2 bound to various anionic PM lipids in vitro, with no clear preference for any particular lipid^53^. Here, purified recombinant ATM2(CT) carrying a C-terminal His_6_ tag (ATM2(CT)–His_6_) again bound to various lipids in in vitro lipid overlay assays (Fig. 3a), including to all phosphoinositides, phosphatidylserine (PtdSer) and phosphatidic acid (PtdOH). By contrast, the ATM2(CTΔLB)–His_6_ variant displayed no detectable binding to any lipids, similar to the negative control, maltose-binding protein (MBP)–His_6_ (Fig. 3a). Coomassie-stained SDS–PAGE gels of the protein fragments used are shown in Fig. 3b. Non-cropped SDS–PAGE gels can be found in the Source Data. To obtain more quantitative information on lipid binding, we analysed purified recombinant fusions of ATM2 fragments to superfolder GFP (sfGFP) by microscale thermophoresis (MST) (Fig. 3c–f)^64^, where lipid ligands can be presented in a liposome regime that more closely resembles a biological membrane^65^. Here we used dioleoyl-phosphatidylcholine (DOPC) as a liposome backbone, to which 20 mol% of various lipids were added (Fig. 3c–e). The MST analysis shows that an sfGFP-tagged C-terminal fragment of ATM2 bound all anionic lipids tested with similar affinities (Fig. 3c,f), including PtdIns3P, PtdIns4P, PtdIns(3,5)P_2_, PtdIns(4,5)P_2_, PtdSer and PtdOH. The carrier lipid, PtdCho, was bound with substantially lower affinity (Fig. 3c,f), and there was no detectable binding of the sfGFP tag alone to either liposome regime used (Fig. 3d). Deleting the C-terminal lipid-binding region of ATM2 resulted in a 400× reduction in lipid-binding affinity, here tested against PtdIns(4,5)P_2_ (Fig. 3e,f). Coomassie-stained SDS–PAGE gels of the protein fragments used for the MST analyses are shown in Fig. 3g. Non-cropped SDS–PAGE gels can be found in the Source Data. The data confirm that the C-terminal portion of ATM2 is necessary and sufficient for ATM2–lipid interactions and that ATM2 displays no clear preference for binding to any anionic lipid in vitro.

### PtdIns(4,5)P_2_ depletion abolishes ATM2 effects on actin dynamics

**Fig. 4:**
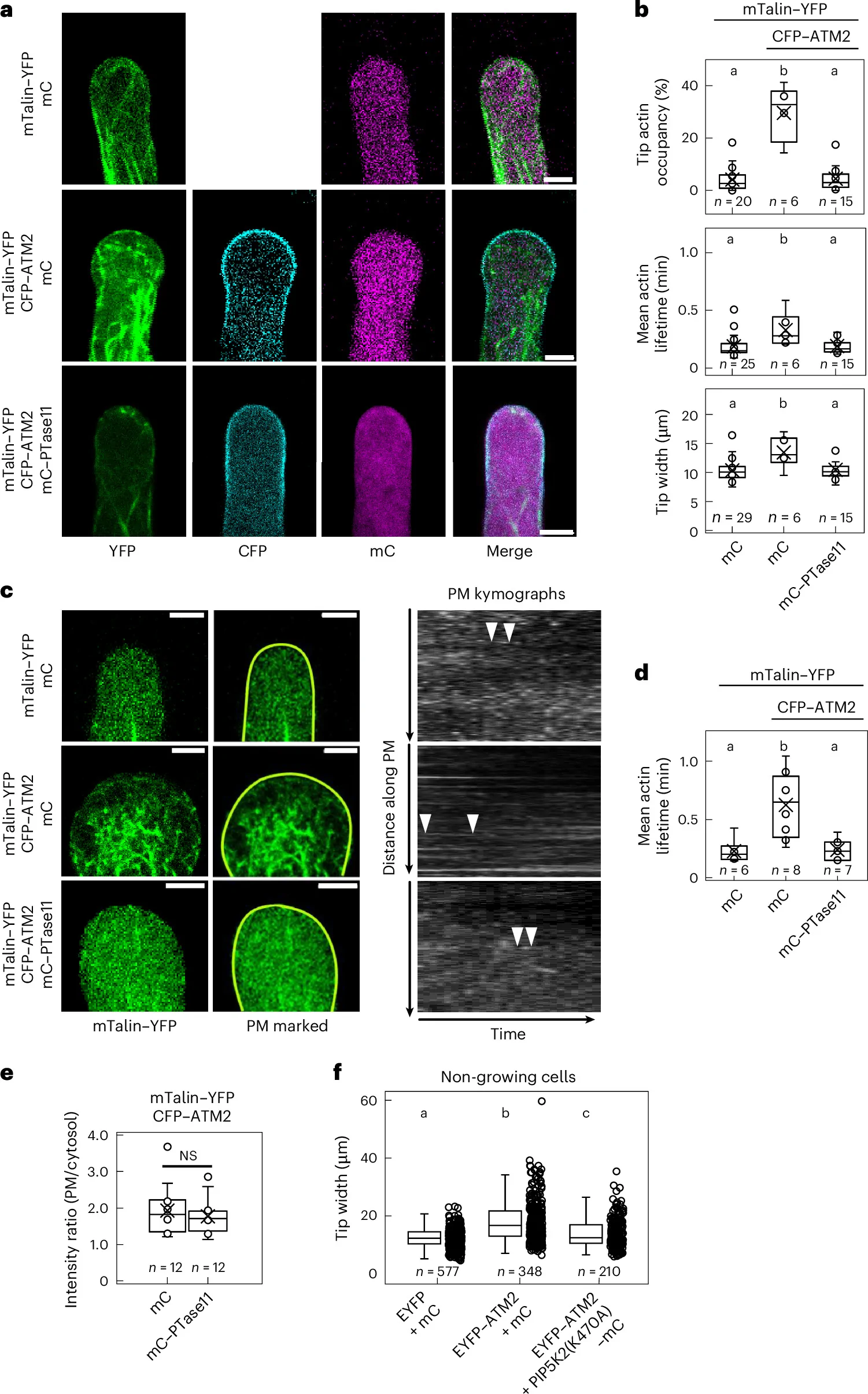
PtdIns(4,5)P_2_ depletion in the PM abolishes ATM2 effects on actin dynamics. The effects of altered PM PtdIns(4,5)P_2_ abundance on the function of ATM2 were studied for pollen tubes coexpressing CFP–ATM2 either with an mC control or with the PtdIns(4,5)P_2_-specific 5-phosphatase mC–*At*PTase11, an enzyme converting PtdIns(4,5)P_2_ to PtdIns4P. Transgenic pollen tube cells were then analysed for tip actin occupancy, mean actin lifetime and apical tip swelling. Expression was driven by a *Lat52* promoter. **a**, Representative fluorescence images of pollen tubes coexpressing mTalin–YFP with an mC control (top row), with CFP–ATM2 and an mC control (middle row) or with CFP–ATM2 and mC–*At*PTase11 (bottom row). Individual fluorescence channels and merged images are shown. Scale bars, 5 µm. **b**, Apical actin occupancy (top), mean actin lifetime of mTalin–YFP-labelled actin filaments (middle) and pollen tube tip width (bottom). The data represent observations from 20–29 cells (mC-control), 6 cells (CFP–ATM2 plus mC) or 15 cells (mC–*At*PTase11). Data were collected from at least three independent experiments. Statistical differences were assessed by one-way ANOVA followed by Tukey’s post-hoc test; letters denote categories of values exhibiting statistically significant differences (for tip actin occupancy, *P* = 1.15 × 10^−12^; for mean actin lifetime, *P* = 7.79 × 10^−3^; for tip width, *P* = 2.28 × 10^−3^). **c**, The mean actin lifetime of PM-proximal actin decorated by mTalin–YFP was recorded by acquiring kymographs along the PM periphery of the cell apex (yellow lines). Left: images illustrating the analysis done. Right: PM kymographs recorded along the yellow lines. Quantification is shown in **d**. Scale bars, 5 µm. **d**, PM-peripheral mean actin lifetime (mTalin–YFP) for control pollen tubes expressing mC and for cells coexpressing CFP–ATM2 either with an mC control or with mC–*At*PTase11. The data represent observations from 6 cells (mC/CFP control), 8 cells (CFP–ATM2/mC) or 7 cells (CFP–ATM2/mC–*At*PTase11). Data were collected from at least three independent experiments. Statistical differences were assessed by one-way ANOVA followed by Tukey’s post-hoc test; letters denote categories of values exhibiting statistically significant differences (*P* = 1.03 × 10^−3^). **e**, Degree of PM association of CFP–ATM2 in control cells coexpressing mC or in cells coexpressing mC–PTase11 according to the fluorescence intensity ratio of mC signals at the PM versus cytosol. The data represent observations from 12 cells for both mC and mC–PTase11. Statistical differences were assessed by one-way ANOVA followed by Tukey’s post-hoc test. NS, not significant (*P* = 0.5494). **f**, Apical tip swelling was assessed in tobacco pollen tubes coexpressing EYFP and mC controls, EYFP–ATM2 plus mC or EYFP–ATM2 plus the catalytically dead PIP5K2(K470A)–mC. Statistical differences were assessed by one-way ANOVA followed by Tukey’s post-hoc test; letters denote categories of values exhibiting statistically significant differences (*P* = 6.06 × 10^−61^). In **b** and **d**–**f**, the centre lines show the medians, the Xs indicate the means, the box limits indicate the 25th and 75th percentiles and the whiskers extend to 1.5 times the interquartile range from the 25th and 75th percentiles. Data points outside this range are plotted as individual outliers.

**Extended Data Fig. 5:**
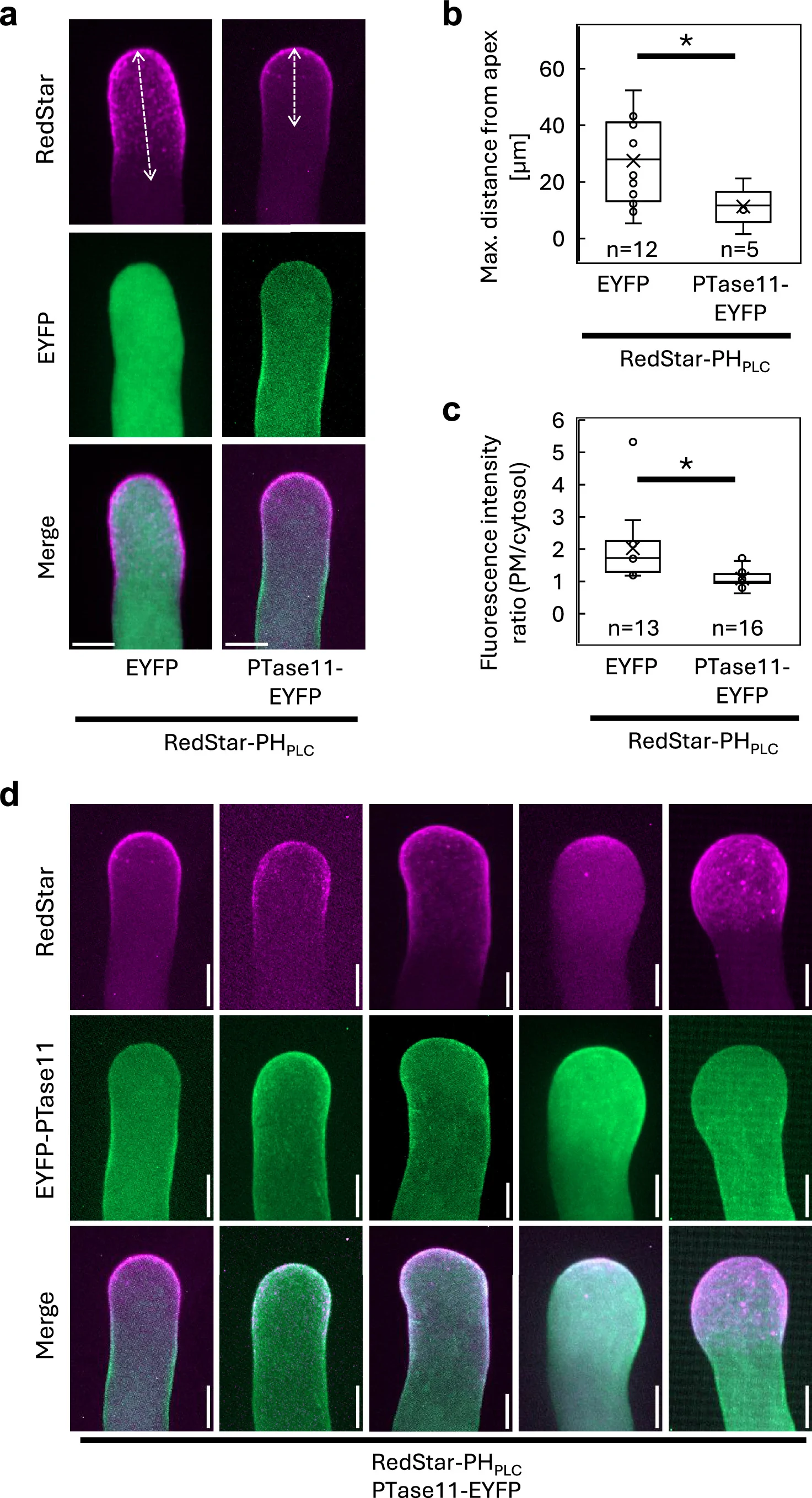
Expression of Ptase11-EYFP reduces PM-association and the extent of the PM region occupied by the PtdIns(4,5)P_2_-specific biosensor, RedStar-PH_PLC_, in tobacco pollen tubes. To document the effects of Ptase11-EFP expression on the distribution of RedStar-PH_PLC_, the fluorescence distribution of the biosensor was analyzed by LSM in tobacco pollen tubes coexpressing either an EYFP control or Ptase11-EYFP. Expression of all constructs was driven by a *pLat52* promoter. (**a**) Representative images of pollen tubes coexpressing RedStar-PH_PLC_ with EYFP (top panels) with Ptase11-EYFP (mid panels). Individual fluorescence channels and merged images (bottom panels), as indicated. Scale, 5 µm. (**b**) PM association of RedStar-PH_PLC_ was assessed according to the PM/cytosol ration of detected fluorescence. Data are from 13 (EYFP) and 16 pollen tubes (Ptase11-EYFP), respectively. (**c**) The spatial extent of the PM region occupied by RedStar-PH_PLC_ was assessed by determining the maximum distance from the cell apex (indicated by arrows in (**A**)). Data are from 12 (EYFP) or 5 pollen tubes (Ptase11-EYFP). (**b**, **c**) Data represent 3-4 independent biological experiments. Center lines show the medians, the x indicate the means. Box limits indicate the 25th and 75th percentiles. Whiskers extend 1.5 times the interquartile range from the 25th and 75th percentiles. Data points outside this range are plotted as individual outliers. The asterisks indicate a significant change from the wild type control according to a 2-tailed Student’s T-test (max. distance from apex, *, p = 0.04084; fluorescence intensity ratio (PM/cytosol), *, p = 0.013). (**d**) Images for the coexpression of RedStar-PH_PLC_ and Ptase11-EYFP (n = 5). Scale, 5 µm.

**Extended Data Fig. 6:**
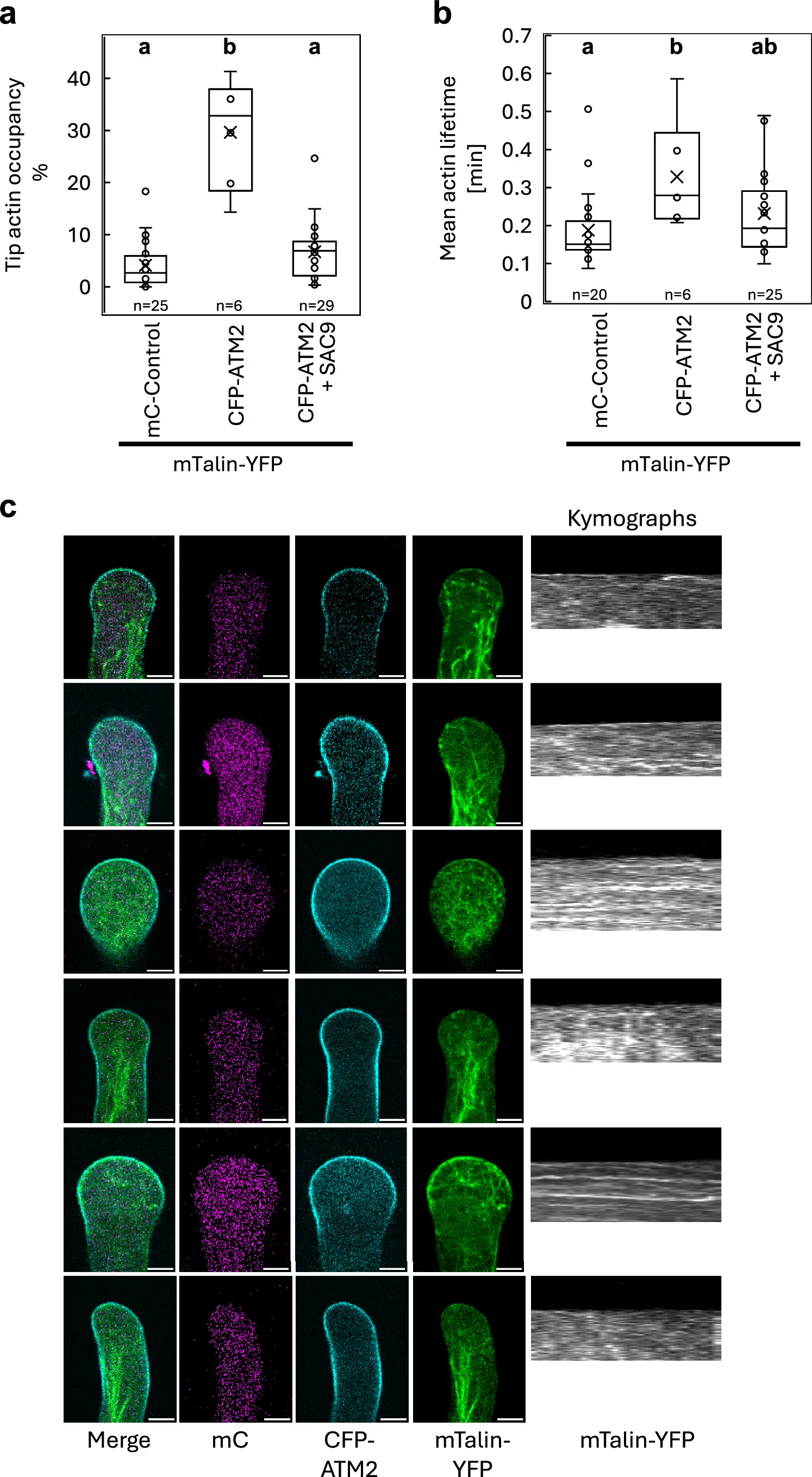
PtdIns(4,5)P_2_ depletion in the PM upon expression of SAC9 abolishes ATM2 effects on actin dynamics. Effects of altered PM PtdIns(4,5)P_2_ abundance on the function of ATM2 were studied for pollen tubes coexpressing CFP-ATM2 either with an mC control, or with the PtdIns(4,5)P_2_-specific 5-phosphatase, SAC9, an enzyme converting PtdIns(4,5)P_2_ to PtdIns4P. Transgenic pollen tube cells were then analyzed for cell morphology or mean actin lifetime. Expression was driven by a *Lat52* promoter. (**a**) Actin occupancy in the cell apex was determined for cells expressing fluorescent fusion proteins, as indicated. Data were collected from at least two independent experiments (mC-control, 25 cells; CFP-ATM2, 6 cells; mC-SAC9, 25 cells). (**b**) Mean actin lifetime of mTalin-YFP signals was recorded by acquiring kymographs. Data were collected from at least three independent experiments (mC-control, 20 cells; CFP-ATM2, 6 cells; mC-SAC9, 25 cells). (**a**, **b**) Center lines show the medians, the x indicate the means. Box limits indicate the 25th and 75th percentiles. Whiskers extend 1.5 times the interquartile range from the 25th and 75th percentiles. Data points outside this range are plotted as individual outliers. Statistical differences were assessed by one-way ANOVA followed by Tukey’s post-hoc test; letters denote categories of values exhibiting statistical significant differences (Tip actin occupancy, p = 3.45368 × 10^−12^; Mean actin lifetime, p < 0.0230722). (**c**) Images for the coexpression of mC, CFP-ATM2 and mTalin-YFP (n = 6). Left panels, merged images, as indicated. Right panels, Kymographs for the mTalin-YFP signal of pollen tube tip regions. Scale, 5 µm.

While the lipid-binding data indicated no binding of ATM2 to any particular lipid, the reduced actin dynamics upon overexpressing mC–ATM2 resembled the effects of overproducing PtdIns(4,5)P_2_ (refs. ^23,24,26^). We therefore tested whether PtdIns(4,5)P_2_ was required for ATM2 effects on actin dynamics. The influence of PtdIns(4,5)P_2_ on the function of full-length ATM2 was assessed in pollen tube cells upon depletion of PtdIns(4,5)P_2_ by the coexpressed PtdIns(4,5)P_2_-specific 5-phosphatase PTase11 (Fig. 4a–e). PTase11 specifically degrades PtdIns(4,5)P_2_ to PtdIns4P and has previously been described as an enzyme that can counteract the effects of increased PtdIns(4,5)P_2_ biosynthesis in pollen tubes^66^. When coexpressed with a RedStar–PH_PLC_ biosensor for PtdIns(4,5)P_2_, PTase11–EYFP reduced the spatial extent of the PM region occupied by RedStar–PH_PLC_ in tobacco pollen tubes, compared with an EYFP control (Extended Data Fig. 5). While coexpression with mC–PTase11 did not alter the PM association of CFP–ATM2 (Fig. 4e), the stabilizing effect of CFP–ATM2 on actin decorated by an mTalin–YFP marker in pollen tube cells was abolished (Fig. 4a–d), resulting in morphologically normal cell shapes. In the presence of mC–PTase11, expressed CFP–ATM2 no longer influenced tip actin occupancy, mean actin lifetime or pollen tube tip width (Fig. 4b). Similar data were obtained by using the coexpression of full-length ATM2 with SAC9 (refs. ^67,68^), another PtdIns(4,5)P_2_-specific 5-phosphatase of the suppressor of actin (SAC) family (Extended Data Fig. 6), indicating that the abolished function of ATM2 was a consequence of PtdIns(4,5)P_2_ depletion. To assess whether the artificial depletion of PtdIns(4,5)P_2_ by mC–PTase11 specifically influenced the PM-proximal stabilization of actin by CFP–ATM2, we next assessed PM-proximal actin dynamics by PM-focused kymograph analysis for an mTalin–YFP marker, as indicated in Fig. 4c,d. While the expression of CFP–ATM2 alone resulted in an enhanced lifetime of PM-proximal actin signals (Fig. 4c,d), this effect was abolished to control levels upon coexpression of CFP–ATM2 with mC–PTase11 (Fig. 4c,d), indicating that the artificial depletion of PtdIns(4,5)P_2_ rendered full-length ATM2 ineffective. While mC–ATM2 displayed pronounced PM association (Fig. 2a,d) that was lost upon the deletion of the lipid-binding C terminus in the mC–ATM2(ΔLB) variant (Fig. 2d), the CFP–ATM2 marker retained PM association upon coexpression with mC–PTase11 (Fig. 4a,e), even though the effects of CFP–ATM2 on cell morphology and actin dynamics were abolished (Fig. 4a,b). Evidently, binding to lipids other than PtdIns(4,5)P_2_ enables the PM association of ATM2, but PtdIns(4,5)P_2_ is required for the effect on actin. A coexpressed catalytically dead variant of PIP5K2–mC (PIP5K1(K470A)–mC) exerted a dominant negative effect on pollen tube tip swelling arising from the expression of EYFP–ATM2 (Fig. 4f), consistent with the notion that ATM2 function requires PtdIns(4,5)P_2_ formation by PIP5K2. The combined data lead us to conclude that PtdIns(4,5)P_2_ is required for the effect of ATM2 on actin dynamics and that lipid-mediated PM recruitment of ATM2 is by itself not sufficient to enable its regulatory effects on actin. These conclusions suggest added regulatory interplay between ATM2 and PtdIns(4,5)P_2_, beyond mere PM recruitment.

### ATM2 promotes PtdIns(4,5)P_2_ formation in vivo and in vitro

**Fig. 5:**
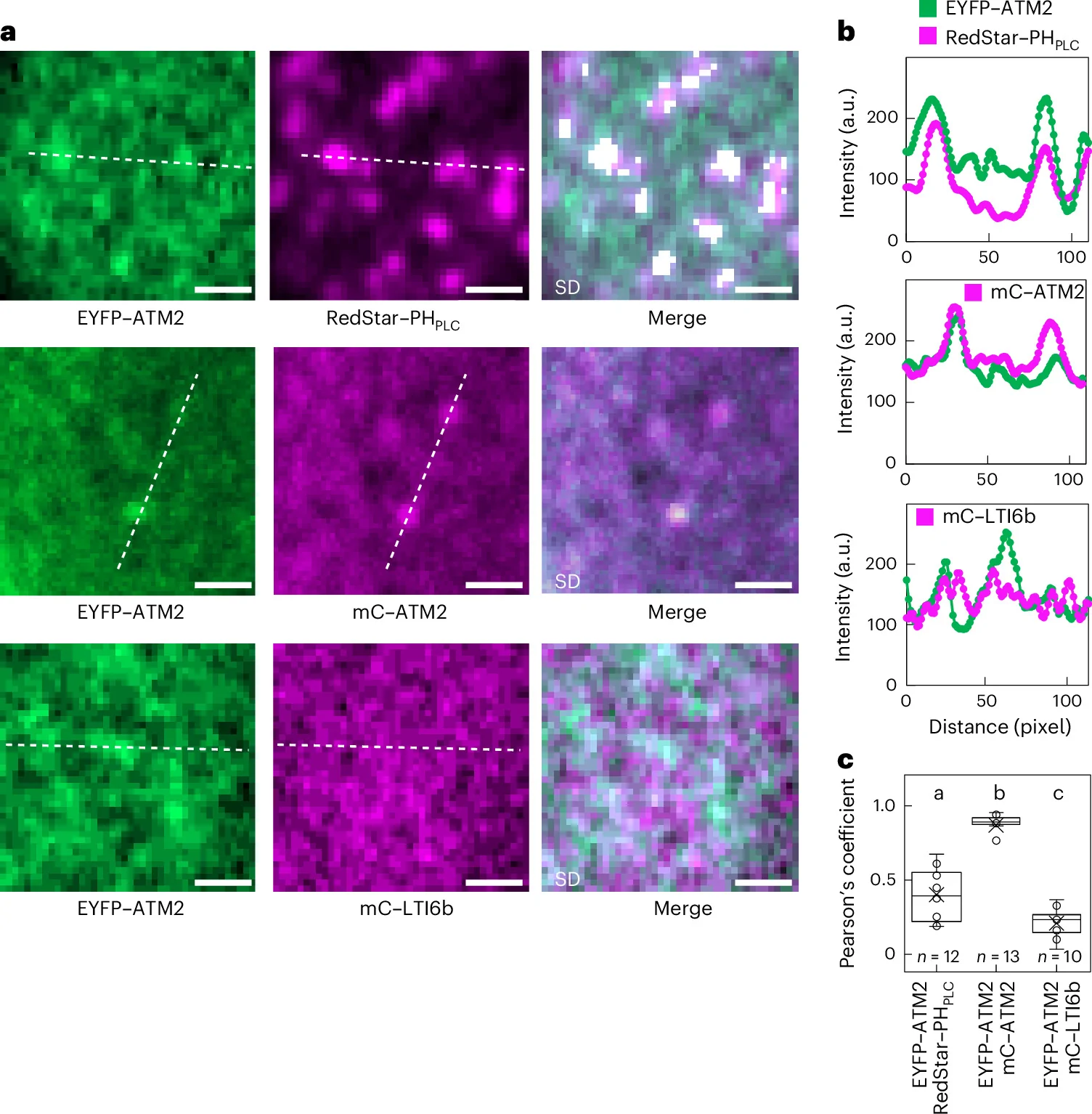
EYFP–ATM2 partially colocalizes with RedStar–PH_PLC_ in PM nanodomains. The fluorescence distribution of EYFP–ATM2 and a coexpressed PtdIns(4,5)P_2_ biosensor, RedStar–PH_PLC_, was recorded by SD in tobacco pollen tubes. Expression was driven by a *Lat52* promoter. **a**, Coexpression of EYFP–ATM2 and RedStar–PH_PLC_ (top) and control experiments, including coexpression of EYFP–ATM2 and mC–ATM2 (middle) or EYFP–ATM2 and the diffuse PM marker mC–LTI6b (bottom). Images are maximum-intensity projections from *Z*-stacks acquired in 0.3-µm slices. EYFP channel (left), RedStar/mC channel (middle) and merged images (right) are shown. Scale bars, 5 µm. **b**, Fluorescence intensity plots for the EYFP and RedStar channels, recorded along the dashed lines in **a**. **c**, Pearson’s colocalization coefficients for EYFP versus red fluorescence distributions from images as shown in **a**, representing 12 cells, *n* = 7 (EYFP–ATM2/RedStar–PH_PLC_); 13 cells, *n* = 2 (EYFP–ATM2/mV–ATM2); and 10 cells, *n* = 2 (EYFP–ATM2/mC–LTI6b). Statistical differences were assessed by one-way ANOVA followed by Tukey’s post-hoc test; letters denote categories of values exhibiting statistically significant differences (*P* = 1.69 × 10^−13^). The centre lines show the medians, the Xs indicate the means, the box limits indicate the 25th and 75th percentiles and the whiskers extend to 1.5 times the interquartile range from the 25th and 75th percentiles. Data points outside this range are plotted as individual outliers.

**Fig. 6:**
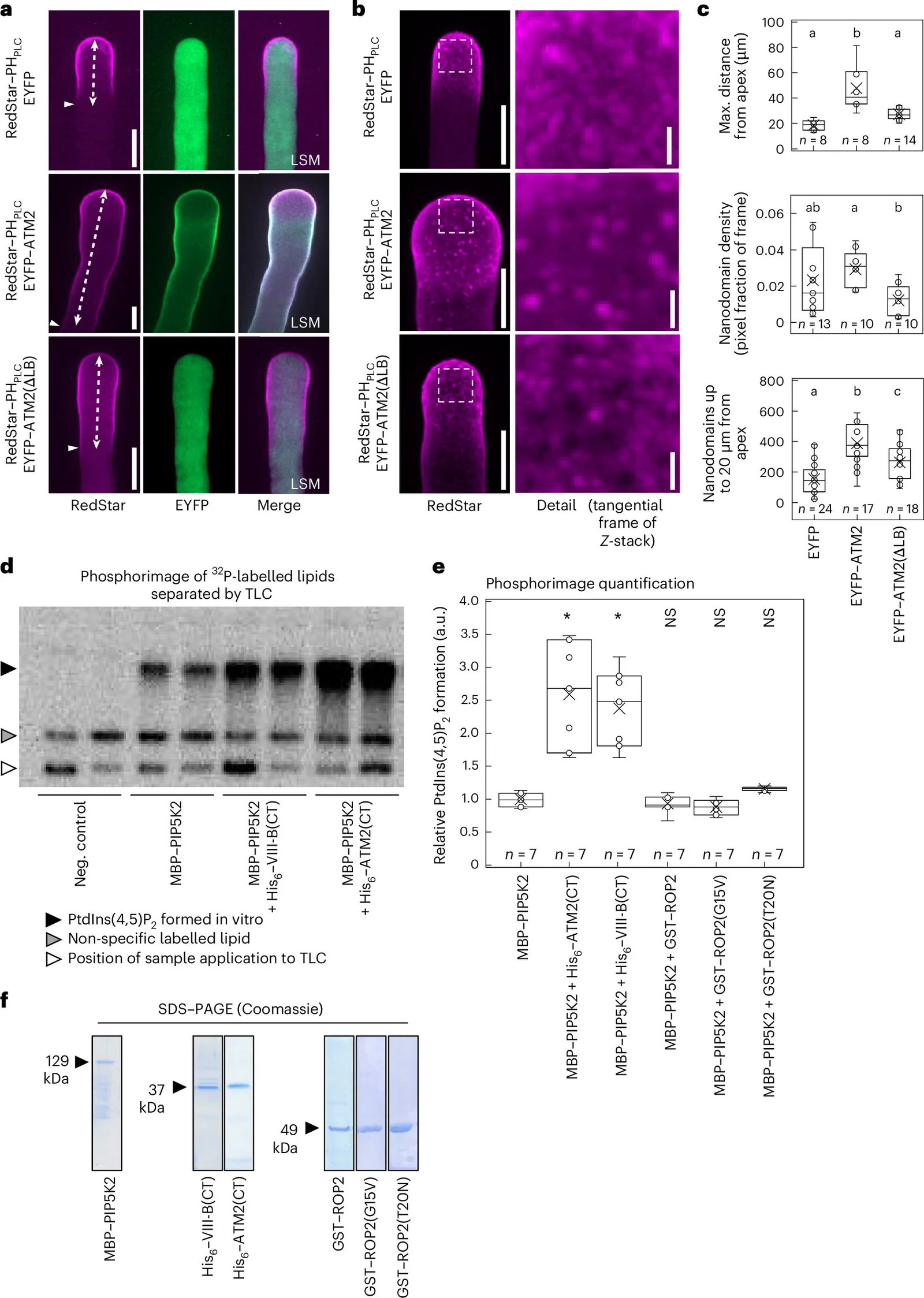
EYFP–ATM2 promotes PtdIns(4,5)P_2_ formation by activating PIP5K2. The functional interplay of ATM2 with PtdIns(4,5)P_2_ and PIP5K2 was analysed in more detail by in vivo and in vitro experiments. **a**, Spatial extent of the subapical PM domain decorated by RedStar–PH_PLC_ upon coexpression with an EYFP control (top), with EYFP–ATM2 (middle) or with EYFP–ATM2(ΔLB) (bottom). Images were acquired by LSM. Note the loss of PM association for EYFP–ATM2(ΔLB). The arrows indicate the extent of the RedStar–PH_PLC_-labelled membrane domain as the maximum distance from the apex. Scale bars, 5 µm. **b**, Nanodomain patterns of RedStar–PH_PLC_ upon coexpression with an EYFP-control (top), with EYFP–ATM2 (middle) or with EYFP–ATM2(ΔLB) (bottom). Images were acquired by SD. Only the RedStar channel is shown. Scale bars, 10 µm. The right panels show detailed views of SD surface scans, representing a substack of the two most PM-peripheral confocal sections for the regions of interest marked by the squares in the respective left panels. Scale bars, 1 µm. **c**, Top: the spatial extent of the PM domain labelled by RedStar–PH_PLC_ upon coexpression with the EYFP control, EYFP–ATM2 or EYFP–ATM2(ΔLB) was measured as the maximum distance from the apex, as indicated by arrows in **a**. The data represent observations from 8 cells (EYFP control), 8 cells (EYFP–ATM2) or 14 cells (EYFP–ATM2(ΔLB)). Middle: the area density of punctate signals decorated by RedStar–PH_PLC_ was analysed as the fraction of fluorescent pixels per frame from SD images from pollen tubes expressing the EYFP control, EYFP–ATM2 or EYFP–ATM2(ΔLB), as indicated in **b**. The data represent observations from 13 cells (EYFP control), 10 cells (EYFP–ATM2) or 10 cells (EYFP–ATM2(ΔLB)). Bottom: the number of nanodomains per cell was counted in a PM region up to 20 µm from the cell apex. The data represent observations from 24 cells (EYFP control), 17 cells (EYFP–ATM2) or 18 cells (EYFP–ATM2(ΔLB)). Statistical differences were assessed by one-way ANOVA followed by Tukey’s post-hoc test; letters denote categories of values exhibiting statistically significant differences (for max. distance from apex, *P* ≤ 7.068 × 10^−6^; for nanodomain density, *P* = 0.0334158; for nanodomains up to 20 µm from tip, *P* = 3.326 × 10^−7^). In **a**–**c**, expression was driven by a *Lat52* promoter. **d**, Purified recombinant MBP–PIP5K2 fusion protein expressed in *E. coli* was tested for in vitro catalytic activity, converting PtdIns4P to PtdIns(4,5)P_2_ in the presence of γ[^32^P]ATP. The formation of radiolabelled PtdIns(4,5)P_2_ was monitored by phosphorimaging after acidic extraction and chromatographic separation of the reaction products. Assays were performed with *E. coli* extract alone as a negative control, with MBP–PIP5K2 alone or upon coincubation of MBP–PIP5K2 with enriched recombinant C-terminal fragments of the class VIII myosins, VIII-B or ATM2, as indicated. The panel shows a representative phosphorimage. The arrowheads indicate the migration of ^32^P-PtdIns(4,5)P_2_ (filled), the migration of a non-specific hydrophobic compound present in the *E. coli* extract (grey) or the origin of migration (open). The experiment was performed five times with similar results. TLC, thin-layer chromatography. **e**, Quantification of PtdIns(4,5)P_2_ production from the assays shown in **d**, reported in relative units. Additional controls included coincubation of MBP–PIP5K2 with the small GTPase ROP2 (a PtdIns(4,5)P_2_-binding protein) and its constitutively active (G15V) or dominant-negative (T20N) variants. The asterisks indicate statistical differences from the MBP–PIP5K2 control, according to a Student’s *t*-test (for His_6_–ATM2, *P* = 0.000199878; for His_6_–VIII-B, *P* = 5.8808 × 10^−5^; for GST–ROP2, *P* = 0.316415; for GST–ROP2(G15V), *P* = 0.06479; for GST–ROP2(T20N), *P* = 0.238928). In **c** and **e**, the centre lines show the medians, the Xs indicate the means, the box limits indicate the 25th and 75th percentiles and the whiskers extend to 1.5 times the interquartile range from the 25th and 75th percentiles. Data points outside this range are plotted as individual outliers. **f**, Coomassie-blue-stained SDS–PAGE gel of enriched recombinant fusion proteins used for the in vitro lipid phosphorylation assays. The deduced protein (fragment) masses are indicated. Source data

While there was no preference of ATM2 for binding to PtdIns(4,5)P_2_ in vitro (Fig. 3), the depletion of PtdIns(4,5)P_2_ by PTase11 or SAC9 abolished ATM2 effects on actin dynamics at the PM (Fig. 4c,d) but not its PM association (Fig. 4e), leading us to speculate that an additional, PM-associated process might exert a relevant contribution to actin stabilization by ATM2. When the lateral PM distribution of coexpressed EYFP–ATM2 and RedStar–PH_PLC_ was observed by SD (Fig. 5), the markers displayed largely overlapping patterns (Fig. 5a, top; see also Fig. 5b,c). Control experiments showed close-to-perfect colocalization of coexpressed EYFP–ATM2 and mC–ATM2 markers (Fig. 5a, middle; see also Fig. 5b,c) versus a significantly lower degree of colocalization of EYFP–ATM2 with a diffuse PM marker, mC–LTI6b^69^ (Fig. 5a, bottom; see also Fig. 5b,c). Pearson’s colocalization coefficients for all three combinations of markers indicate that the degree of colocalization of EYFP–ATM2 and RedStar–PH_PLC_ was significantly higher than the coincidental overlap of EYFP–ATM2 with mC–LTI6b (Fig. 5c). While analysing the distribution of the RedStar–PH_PLC_ biosensor upon coexpression of different ATM2 variants, we noticed that the distribution of the RedStar–PH_PLC_ marker was influenced by coexpression with the PM-associating EYFP–ATM2, but not by coexpression with an EYFP control or the lipid-binding-deficient variant, EYFP–ATM2(ΔLB) (Fig. 6a). The extent of the subapical PM domain marked by RedStar–PH_PLC_ was significantly expanded when EYFP–ATM2 was coexpressed, whereas no significant effects were observed upon coexpression of EYFP or EYFP–ATM2(ΔLB) (Fig. 6a). Figure 6b shows representative SD images of RedStar–PH_PLC_-decorated PM nanodomains upon coexpression with EYFP (top), EYFP–ATM2 (middle) or EYFP–ATM2(ΔLB) (bottom). Quantitative analysis of the patterns of RedStar–PH_PLC_ distribution indicates that expression of EYFP–ATM2 resulted in an expanded area of the PM being occupied by the biosensor and in overall more PtdIns(4,5)P_2_ nanodomains per cell (Fig. 6c). By contrast, the area density of RedStar–PH_PLC_ signals was significantly reduced when EYFP–ATM2(ΔLB) was expressed. Together, the data indicate a promoting effect of PM-associated EYFP–ATM2 on the abundance of PtdIns(4,5)P_2_ in the subapical PM.

As PtdIns(4,5)P_2_ with a role in controlling actin dynamics is formed by PIP5K2 (ref. ^23^), and PIP5K2 interacted with ATM2 (Fig. 1f,g), we hypothesized that ATM2 might exert an effect on PtdIns(4,5)P_2_-formation by PIP5K2. In vitro PI4P 5-kinase activity tests with purified recombinant PIP5K2 protein were performed in the absence or presence of purified recombinant fragments of ATM2 or VIII-B proteins (Fig. 6d). All proteins were recombinantly expressed in *Escherichia coli*, PIP5K2 as a fusion to an N-terminal MBP tag and a C-terminal His_6_ tag^21^, and C-terminal fragments of ATM2 or VIII-B as fusions to N-terminal His_6_ tags^53^. The in vitro lipid phosphorylation assays with PtdIns4P substrate (Fig. 6d) show that PtdIns(4,5)P_2_ formation by MBP–PIP5K2 (filled arrowhead) was substantially enhanced by either of the added class VIII myosin fragments, His_6_–ATM2 or His_6_–VIII-B (Fig. 6d,e). A negative control using *E. coli* extract alone did not result in PtdIns(4,5)P_2_ formation (Fig. 6d, left). Quantitative analysis of the ^32^P-labelled PtdIns(4,5)P_2_ reaction product indicates significant activation of MBP–PIP5K2 in the presence of His_6_–ATM2 or His_6_–VIII-B in vitro (Fig. 6e). Further PIP5K2 activity assays were performed in the presence of recombinant wild-type ROP2 from *Arabidopsis* or its constitutively active or dominant-negative substitution variants, ROP2(G15V) or ROP2(T20N)^70^, and these proteins all had no effect on PtdIns(4,5)P_2_ formation by PIP5K2 in the in vitro tests (Fig. 6e). Coomassie-stained SDS–PAGE gels of all protein fragments used are shown in Fig. 6f. Non-cropped SDS–PAGE gels can be found in the Source Data.

The in vitro activity tests with purified recombinant proteins are consistent with ATM2 exerting a regulatory effect on PIP5K2 activity. Importantly, the enhanced in vitro activity of purified PIP5K2 in the presence of purified ATM2 or VIII-B fragments indicates that the activation of PtdIns(4,5)P_2_ formation required no further elements. In sum, the data from the in vivo observation of the altered distribution of the PtdIns(4,5)P_2_ biosensor, RedStar–PH_PLC_, in tobacco pollen tubes (Fig. 6a–c) and from in vitro experiments (Fig. 6d,e) are consistent with a promoting effect of ATM2 on the formation of PtdIns(4,5)P_2_ in the subapical PM of pollen tube cells.

## Discussion

### ATM2 reduces actin dynamics at the PM–actin interface

ATM2 is thought to contribute to forming an actin–PM interface by binding to actin via its N-terminal actin-binding region and to the PM via its C-terminal polybasic lipid-binding domain^53^. Here we show not only that ATM2 is a tether for actin filaments at the PM but also that ATM2 exerts a regulatory effect on the PM lipid landscape to control actin dynamics (Figs. 2 and 4 and Extended Data Figs. 2 and 3). The reduced actin dynamics upon expressing ATM2 in pollen tubes resemble the effects of overproducing ROPs^35^, or of PtdIns(4,5)P_2_ via overexpressing nanodomain-associated PIP5K2 (refs. ^23,26^). Our data provide information on the so-far-elusive molecular role of class VIII myosins, which serve as adaptors between actin filaments and the PM and exert a stabilizing effect on actin that is linked to regulatory functions of PtdIns(4,5)P_2_ (refs. ^23,24,26^).

PtdIns(4,5)P_2_ is required for ATM2-mediated effects on actin dynamics, according to the two key findings (1) that the effects of full-length ATM2 on actin dynamics were abolished upon coexpression with the PtdIns(4,5)P_2_-degrading enzymes PTase11 (Fig. 4a–e) and SAC9 (Extended Data Fig. 6) and (2) that coexpression with the catalytically dead PIP5K2(K470A)–EYFP exerted a dominant negative effect on ATM2-mediated pollen tube tip swelling (Fig. 4f). As the expression of the PtdIns(4,5)P_2_-binding portion of ATM2, mC–ATM2, by itself influenced actin dynamics and cell morphology in non-growing pollen tubes (Fig. 2d,e and Extended Data Fig. 3c,d), we conclude that the N-terminal domains of ATM2 also have a role in mediating ATM2 effects on actin dynamics, possibly by mediating interactions with actin or with other relevant proteins. Actin stabilization by ATM2 was observed mostly at the PM (arrowheads in Figs. 2a,d and 4c), consistent with the notion that a PtdIns(4,5)P_2_-dependent, PM-proximal process mediates ATM2 effects on actin dynamics. Interestingly, the PM association of CFP–ATM2 was retained in the presence of mC–PTase11 (Fig. 4a,e), which upon coexpression nonetheless suppressed ATM2-mediated effects on actin dynamics and cell morphology (Fig. 4a–d). As PTase11 and SAC9 convert PtdIns(4,5)P_2_ to PtdIns4P, which can be bound by ATM2 as well as other anionic PM lipids (Fig. 3), such non-specific binding to anionic lipids might mediate the membrane association of ATM2. We conclude that the PtdIns(4,5)P_2_-dependent actin-stabilizing effect of ATM2 involves more steps than mere membrane recruitment of ATM2, and we propose that the observed activation of PIP5K2 and enhanced PtdIns(4,5)P_2_ formation (Fig. 6) are relevant in this context.

### ATM2 activates PIP5K2, promoting PtdIns(4,5)P_2_ formation

So far, for plant models, the intrinsic molecular mechanisms that control how and when PtdIns(4,5)P_2_ is formed have remained elusive. Previous reports on the effects of PtdIns(4,5)P_2_ have relied on the artificial perturbation of PtdIns(4,5)P_2_ formation—for example, by artificially overproducing or underproducing PtdIns(4,5)P_2_ (refs. ^18–26^)—demonstrating the essential involvement of PtdIns(4,5)P_2_ in secretion^18,19^, endocytosis^22^ and the control of actin dynamics^23,24^. However, only a few studies have shown how PtdIns(4,5)P_2_ formation might be intrinsically regulated—for instance, by phosphorylation of biosynthetic enzymes^71–74^. There is currently no report of intrinsic regulation of PIP5K2 with a role in controlling actin dynamics.

The observation of altered fluorescence distribution of the PtdIns(4,5)P_2_ biosensor RedStar–PH_PLC_ upon coexpression with ATM2–EYFP (Fig. 6a–c) indicates that ATM2 might contribute to the intrinsic regulation of PtdIns(4,5)P_2_ production in vivo. In pollen tubes, PtdIns(4,5)P_2_ with a role in controlling actin dynamics is mainly formed by the enzyme PIP5K2 (refs. ^23,26^). FRET/FLIM analysis (Fig. 1f), yeast-two-hybrid analysis (Fig. 1g) and the direct in vitro activation of purified recombinant PIP5K2 protein by a purified recombinant C-terminal ATM2 fragment (Fig. 6d,e) indicate that an interaction between ATM2 and PIP5K2 might modulate PtdIns(4,5)P_2_ formation by PIP5K2. Our data are consistent with an intrinsic regulatory effect of ATM2 on PtdIns(4,5)P_2_ formation by PIP5K2, which in vivo probably occurs in PM nanodomains occupied by PIP5K2 (ref. ^23^).

In the absence of structural information on the proposed PIP5K2–ATM2 complex, it remains unclear how the ATM2-mediated activation of PIP5K2 can be explained at the protein-structural level. On the basis of the in vitro data, it appears that the interaction between the C-terminal portion of ATM2 and PIP5K2 is sufficient to promote enhanced PtdIns(4,5)P_2_ formation (Fig. 6d,e). It has previously been proposed that the plant-specific N-terminal domains of *Arabidopsis* PIP5K1 exert an autoinhibitory role^75^. As PIP5K2 is 87% identical in sequence to PIP5K1, it is possible that PIP5K2 is autoinhibited by self-interaction between the catalytic domain of the enzyme and its N-terminal regulatory domains, as previously postulated for PIP5K1 (ref. ^75^). The linker region between the catalytic domain and the N-terminal domains of PIP5K2 is of key importance for the association of PIP5K2 with PM nanodomains^23^ and is specifically required for the effect of PIP5K2 on actin dynamics^23^, leading us to speculate that the linker region might mediate protein–protein interactions with proteins in PM nanodomains mediating actin–PM contacts. As ATM2 is an interacting protein acting in this context, it appears plausible that an interaction of ATM2 with the linker region of PIP5K2 might interfere with the proposed autoinhibition, thereby effecting the activation of PIP5K2 and enhanced PtdIns(4,5)P_2_ production. While this scenario is consistent with previously published data on the catalytic behaviour of plant PI4P 5-kinases^26,75^, the exact molecular mechanism of ATM2-mediated activation of PIP5K2 remains currently unresolved. Current research is directed towards combining molecular modelling and experimental protein structure determination to delineate the mechanism of PIP5K2 activation by ATM2 at the biochemical level.

### A self-reinforcing regulatory loop to stabilize actin–PM attachment

**Fig. 7:**
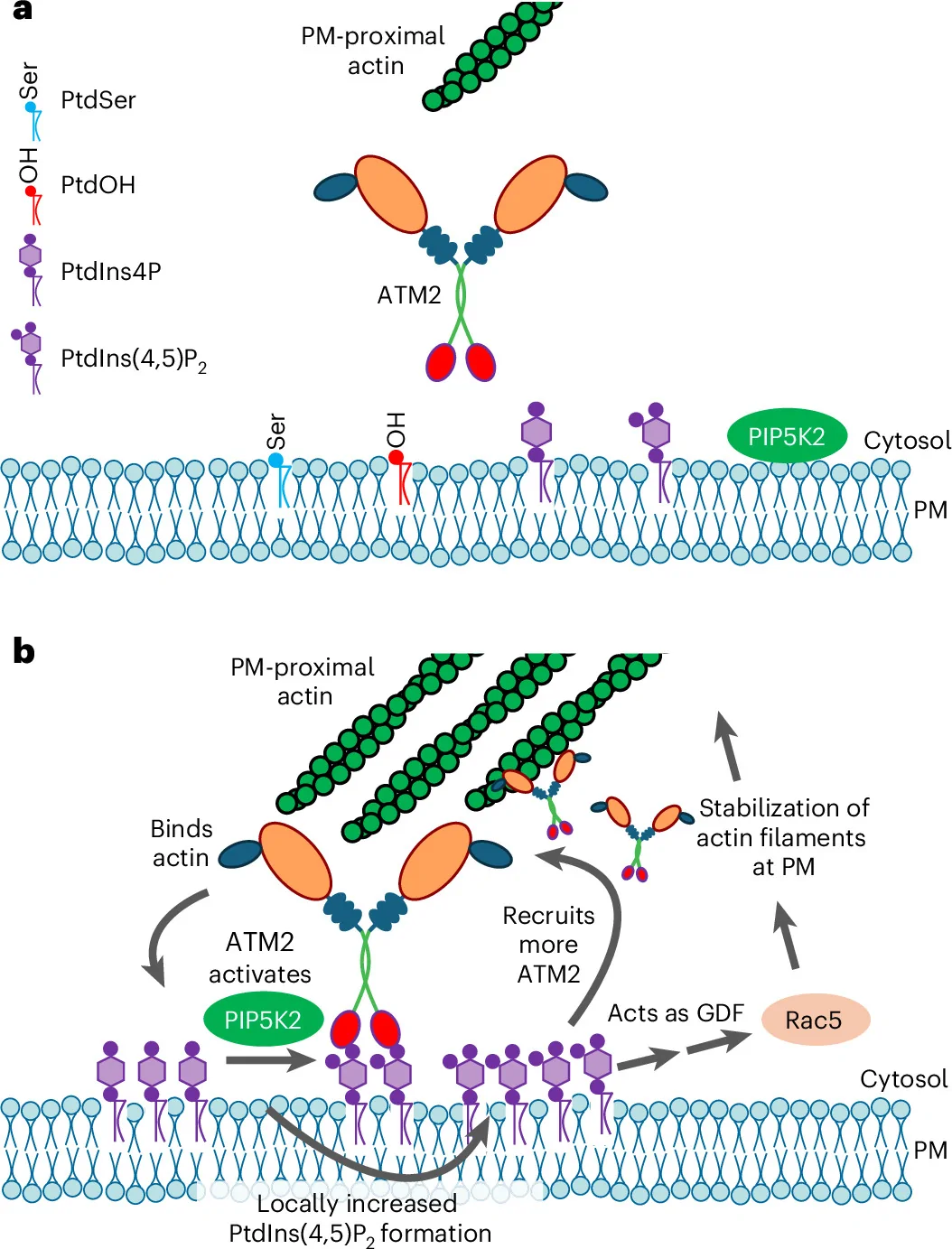
Model of ATM2-mediated local potentiation of PtdIns(4,5)P_2_ production at the actin–PM interface. The class VIII myosin ATM2 forms an interface between actin filaments and the PM by binding to anionic phospholipid headgroups and to actin filaments. **a**, ATM2 can bind various anionic phospholipids at the PM, including PtdSer, PtdOH, PtdIns4P and PtdIns(4,5)P_2_, mediating PM recruitment. However, PM recruitment alone does not induce an effect on actin dynamics, which requires PIP5K2. **b**, When ATM2 encounters PIP5K2 at the PM, it might at least transiently occur in complex with PIP5K2 and further elements of the Rac/ROP signalling system controlling actin dynamics. In the presence of ATM2, the catalytic activity of PIP5K2 is enhanced, thereby promoting PtdIns(4,5)P_2_ formation at actin–PM contact sites. The locally increased abundance of PtdIns(4,5)P_2_ acts as a positional signal for the recruitment of further ATM2 proteins and serves as a GDF on *Nt*RhoGDI2 or related proteins, mediating enhanced activation of Rac/ROP proteins, to facilitate actin stabilization. Alternative explanations are possible. GDF, guanine nucleotide displacement factor; Rac5, tobacco ROP with a role in mediating actin stabilization.

The effects of ATM2 on actin tie into the system of known regulators of actin dynamics, such as PtdIns(4,5)P_2_, ROPs and GDIs. However, the regulation of actin dynamics is probably not a linear series of events based just on ATM2 PM recruitment, as illustrated in the model schematic in Fig. 7.

ATM2 binds generally to anionic membrane lipids, including PtdIns4P, PtdIns(4,5)P_2_, PtdSer and PtdOH, via its C-terminal polybasic region, and PM association is required for ATM2 effects on actin dynamics (Fig. 2d,e and Extended Data Fig. 3). When ATM2 encounters and binds PIP5K2 at the PM, ATM2 locally promotes PtdIns(4,5)P_2_ production (Fig. 6), resulting in enhanced presence of PtdIns(4,5)P_2_ in nanodomains that can serve to recruit more ATM2 proteins as well as other, PtdIns(4,5)P_2_-dependent factors. By activating PIP5K2 and the production of PtdIns(4,5)P_2_ in PM nanodomains, ATM2 affects GDIs (Extended Data Figs. 1b and 3e), resulting in the release of more ROPs for activation at the PM^32^ to promote actin stabilization (Fig. 7). The mutual interdependencies between ATM2, PIP5K2 activation and PtdIns(4,5)P_2_ formation illustrated in Fig. 7 introduce an element of self-reinforcement that might explain why ATM2 effects on actin dynamics may require PM association. In this scenario, only PtdIns(4,5)P_2_ production is locally promoted, making the process PtdIns(4,5)P_2_-dependent without a requirement for high-affinity preferential binding of ATM2 to any specific lipid. The proposed self-reinforcing loop between PtdIns(4,5)P_2_-dependent recruitment of ATM2 and the ensuing ATM2-dependent promotion of PtdIns(4,5)P_2_ formation by PIP5K2 might thus contribute to the intrinsic regulation of PIP5K2 and PtdIns(4,5)P_2_ abundance, which contribute to defining actin–PM contact sites.

In sum, the data indicate that the class VIII myosin ATM2 influences actin dynamics by modulating PM nanostructure. The latter effect resembles that of the class XI myosin XI-K, which contributes to the formation of PM nanodomains during the assembly of cell surface receptor complexes involved in plant immunity^76^. So far, an involvement of PM lipids in myosin XI-K function has not been addressed, and it remains to be seen whether a similar mode of action is involved as shown here for ATM2. Reciprocally, the contribution of class VIII myosins to the physiology of sporophytic tissues is also open for exploration.

### Limitations of this study

The experimental set-up used in this study limits some aspects of interpretation. While our data clearly show an effect of ATM2 on actin dynamics, several limitations should be noted. First, the use of the transient expression system provides evidence based only on gain-of function and dominant-negative effects. Second, effects of the actin markers used on actin dynamics are known, and therefore we aimed to provide data using alternative markers, mTalin and LifeAct. A particular issue relates to the in planta interaction tests of ATM2 against PIP5K2, for which an N-terminally truncated ATM2 variant was selected, as no coexpressors of full-length ATM2 and PIP5K2 survived to be used in the FRET/FLIM experiments. We hope to be able to address the interactive behaviour of full-length ATM2 in the future. Finally, the molecular basis for the observed ATM2-mediated activation of PIP5K2 remains unresolved.

## Methods

### Plant materials and growth conditions

Wild-type *N. tabacum* (ecotype Samsun N) plants were grown on soil in a greenhouse at 21–26 °C and ambient humidity under long-day conditions (16 h light, 8 h darkness) and semi-controlled light intensities.

The *Arabidopsis thaliana* plants used were of the Columbia-0 ecotype. Mutant analysis was done with the alleles SALK_052429 (*atm2*) and SALK_009323 (*viii-b*)^48^. Wild-type and mutant plants were grown in parallel in peat pellets at 21 °C and ambient humidity under long-day conditions (16 h light, 8 h darkness) and light intensity of 110 µmol m^−2^ s^−1^. For the pollen germination assay, pollen grains collected from freshly opened flowers harvested in the morning were evenly spread onto the flattened surfaces of pollen germination medium agarose pads (10% sucrose, 1.5% agarose, 0.01% H_3_BO_3_, 5 mM CaCl_2_, 5 mM KCl, 1 mM MgSO_4_ and 5 mM MES, pH adjusted to 6.0 with KOH) and incubated in a humid chamber in darkness at room temperature for 4 h to allow pollen germination. For germination assessment, a drop of liquid pollen germination medium (pollen germination medium without agarose) was added onto each agarose pad and covered with a coverslip. The entire surface of each pad was systematically imaged using an Olympus Provis AX 70 transmitted light microscope to record all pollen grains present on the pad. For pollen tube morphology analysis, the germinated pollen was stained with propidium iodide solution (stock solution: 1 mg ml^−1^ in distilled water, diluted 1:10 in liquid pollen germination medium) and imaged with an inverted Leica TCS SP8 confocal microscope using an HC PL APO CS2 ×63/1.20 water objective with excitation at 514 nm and emission at 550–658 nm.

### cDNA constructs

#### Constructs for transient expression in pollen tubes

The actin marker constructs LifeAct–RFP and mTalin–YFP were described previously^23^. The RedStar–PLC_PH_ construct was assembled as previously described^18^. The *ATM2* coding sequence was amplified from *Arabidopsis* flower cDNA using the primer combination 5′-ATGCGGCGCGCCATGATGTTATCGGCATCG-3′/5′-ATGCCTCGAGCTAGCCTCTTTTTCCCCACC-3′. The coding sequence for ATM2(ΔLB) (ATM2(1–1177)) was amplified using the primers 5′-ATGCGGCGCGCCATGATGTTATCGGCATCG-3′/5′-ATGCGCGGCCGCCTAAAATTCATCTTCTGG-3′. For *ATM2* (ATM2(972–1220)), the primers 5′-ATGCGGCGCGCCATGCGCAATGTGGCGACA-3′ and 5′-ATGCCTCGAGCTAGCCTCTTTTTCCCCACC-3′ were used. The coding sequence for ATM2(ΔNT) was cloned as previously described^53^. The coding sequence for tobacco *Nt*Myo2-like isoform X2 was amplified from tobacco (*N. tabacum*) cDNA, using the primer combination 5′-ATGCGGCGCGCCATGTTGTCCGTTTCGCATAA-3′/5′-ATGCGCGGCCGCTCACTTGCTCTTCTTACCCCA-3′. Amplicons were moved as AscI/XhoI or AscI/NotI fragments into pEntry::pLAT52 vectors in frame with the coding sequences for EYFP, mC or CFP. The constructs mC–*At*SAC9 and mC–*At*PTase11 were identical to those previously reported^66^. For *At*PTase11, the coding sequence was additionally excised with XhoI and NotI and cloned downstream of the *pEntry*::*pLAT52*::*mC* cassette.

#### Constructs for recombinant expression

The construct encoding ATM2–His_6_ (representing ATM2(972–1220)–His_6_) for bacterial expression was used as described previously^53^. The *ATM2*_*ΔLB*_*-His*_*6*_ construct (representing ATM2(972–1177)–His_6_) was generated using the primer combination 5′-ATCGCATATGCGCAATGTGGCGACAAAA-3′/5′-ATCGGGATCCCTAAAATTCATCTTCTGG-3′ restriction sites and moved into pMalc5G as an NdeI/BamHI fragment. Cloning of the His_6_–MBP construct^14^ and the *MBP-PIP5K2* construct^71^ were described previously. To optimize protein enrichment and detection, a C-terminal His_6_ tag was added by site-directed mutagenesis using the primers 5′-ACCACCACCACTAGAGTCGACGGAT-3′/5′-CCGGTGGTGAGCGTCTTCAA-3′. For sfGFP fusion constructs, the *sfGFP* coding sequence was amplified using the primers 5′-ATGCCCATGGAAAGTAAAGGCGAAGAGCTGTT-3′ and 5′-ATGCGGATGGACGAGCTGTATAAACATATG-3′ and moved as an NcoI/NdeI fragment into *pET28b*. The *ATM2* coding sequence was amplified using the primers 5′-ATCGGTCGACATGCGTAATGTTG-3′/5′-ATCGGGCAAACGTGGCGCGGCCGC-3′ and moved in-frame downstream of sfGFP as a SalI/NotI fragment. The coding sequence for the truncated variant ATM2(CTΔLB) was amplified using the primers 5′-ATCGGTCGACATGCGTAATGTTG-3′/5′-ATCGCAGAAGATGAATTTGCGGCCGCA-3′, and the amplicon was moved into *pET28b* in-frame behind sfGFP as a SalI/NotI fragment, generating a C-terminal His_6_ fusion.

#### Constructs for yeast-two-hybrid analysis

The pBT3C-Ost4 constructs for using PIP5K2 or PIP5K6 as bait were used as described previously (ref. ^77^ for PIP5K2 and ref. ^71^ for PIP5K6). The cDNA encoding the myosin XI-2 bait fragment was amplified using the primer combination 5′-ATGCGGCCATTACGGCCAATGATAAGCACTCCTGTGAGGA-3′/5′-ATGCGGCCGAGGCGGCCAAGTGCAAGAATACAAATGCTGG-3′, and the amplicon was moved as an SfiI/SfiI fragment into the pBT3C-Ost4 plasmid. The coding sequences for the respective C-terminal myosin fragments used as prey proteins and other controls were amplified using the following primer combinations: ATM2(972–1220), 5′-ATGCGGCCATTACGGCCCGCAATGTGGCGACAAAATCAAAG-3′/5′-ATGCGGCCGAGGCGGCCCTAGCCTCTTTTTCCCCACC -3′; RabC2a, 5′-ATGCGGCCATTACGGCCATGGGATCTTCTTCTGGGCAAAG-3′/5′-ATGCGGCCGAGGCGGCCCTAGCTGCAACAGCCGGATTG-3′; RabD1, 5′-ATGCGGCCATTACGGCCATGAGCAACGAGTACGATT-3′/5′-ATGCGGCCGAGGCGGCCCTACTGACCGCAGCAACCGCC-3′; VIII-B(921–1134), 5′-ATGCGGCCATTACGGCCCGCAATGAGAAGAAAAAAAC-3′/5′-ATGCGGCCGAGGCGGCCTCAATAACTTTTCTTGCACC-3′; XI-1(881–1520), 5′-ATGCGGCCATTACGGCCACCGGTGCTCTTAAAGATGC-3′/5′-ATGCGGCCGAGGCGGCCTCAATCTGACCTTTCCAACAAG-3′; XI-2(883–1505), 5′-ATGCGGCCATTACGGCCACTGGAGCACTCCAAGCAGCC-3′/5′-ATGCGGCCGAGGCGGCCTTAGTGCAAGAATACAAATG-3′; XI-A(883–1730), 5′-ATGCGGCCATTACGGCCACAGGAGCACTTCAAGATGC-3′/5′-ATGCGGCCGAGGCGGCCTCATTCCTTGAGAAAGAGGAAG-3′; XI-C(892–1538), 5′-ATGCGGCCATTACGGCCACAGGAGCACTTAAAGAGGC-3′/5′-ATGCGGCCGAGGCGGCCTCACTCAGAGACTGGCAATAAG-3′; XI-E(887–1529), 5′-ATGCGGCCATTACGGCCACAGGCGCACTGAAAGAGGC-3′/5′-ATGCGGCCGAGGCGGCCTTAGTCAGAACATGGCAATAG-3′; XI-H(890–1516), 5′-ATGCGGCCATTACGGCCACGGGAGTCCTTGAAGCTGCC-3′/5′-ATGCGGCCGAGGCGGCCTTATATCAAGAAACTGAAAGC-3′; XI-I(889-1522), 5′-ATGCGGCCATTACGGCCCTTGAGAAACGATTGAGAAC-3′/5′-ATGCGGCCGAGGCGGCCTCAAATGATCTGCTTTGAGG-3′. Amplicons were moved as SfiI/SfiI-fragments into pPR3-N.

All oligonucleotides were commercially synthesized and obtained from Merck/Sigma-Aldrich.

### Overview of cDNA constructs used

The cDNA constructs used in this study are listed in Supplementary Table 2.

### Expression and enrichment of recombinant proteins in *E. coli*

Recombinant proteins were generated as previously described^26,53,71^. In brief, ATM2 variants were expressed in *E. coli* Rosetta 2 (DE3) cells (genotype F− ompT hsdSB(rB− mB−) gal dcm (DE3) pRARE2 (CamR) (Merck). Precultures were grown overnight in 50 ml liquid 2 YT media 1% (w/v) yeast extract, 1.6% (w/v) tryptone, 0.5% (w/v) NaCl and 0.2% (w/v) glucose) at 37 °C with shaking at 150 rpm and used to inoculate 1 l (ATM2) or 3 l (PIP5K2) 2 YT cultures at 0.1 optical density (600 nm) units. Expression cultures were grown in 5 l baffled flasks at 37 °C with shaking at 120 rpm to an optical density (600 nm) of 0.8 units. Expression of recombinant proteins was induced by adding 0.1 mM isopropyl β-d-thiogalactopyranoside and incubating for 4 h (ATM2) or for 2 h (PIP5K2) at 22 °C. Enrichment of recombinant proteins or protein fragments was performed as previously described (ref. ^78^ for MBP–PIP5K2 and ref. ^53^ for C-terminal fragments of VIII-B and ATM2). His_6_-tagged sfGFP fusions were enriched by Ni^2+^-affinity chromatography using 5-ml HisTrap HP (Sigma-Aldrich) columns and an ÄKTA FPLC (GE Healthcare) according to the manufacturer’s instructions.

### Transient expression of cDNA constructs in tobacco pollen tubes

Transient expression of cDNA constructs in tobacco pollen tubes was achieved by particle bombardment exactly as previously described^23^. Gold particles were coated with 4 µg of EYFP construct plasmid DNA, 6 µg of CFP or mC construct plasmid DNA and 5 µg of RedStar–PH_PLC_ plasmid DNA.

### Confocal microscopy

Images were acquired using a Zeiss LSM880 Airyscan system or a Zeiss Cell observer with a Yokogawa CSU-X1 SD unit. For LSM880 imaging, a ×40 objective without immersion was used, and images were captured with ZEN Black image analysis software with the pinhole set to 1 Airy unit. The distribution and dynamics of actin filaments were obtained by using mTalin–YFP as an actin marker. YFP emission was detected at 514 nm using an HFT 514-nm main beam splitter. *Z*-stacks of 2-µm slices were captured as time-lapses of 10-s intervals over the course of 5 min (30 cycles) using the high-resolution *Z* piezo.

For localization studies, *Z*-stacks and time series were captured by SD microscopy. Images were acquired using ×63 oil immersion. First, the cytoplasmic streaming was checked by bright-field acquisition as time-lapse with 10 s/18 cycles (3 min). *Z*-stacks were acquired in 0.3-µm slices. The acquisition time was 400–600 ms for constructs fused to EYFP and 700–900 ms for mC/RFP at 25% laser power.

FRET/FLIM measurements were performed using a Leica STELLARIS 8 FALCON confocal microscope equipped with a ×40 water-immersion objective. EYFP was excited with the 509-nm laser line and mC with the 584-nm laser line. Fluorescence lifetime data were acquired in FLIM mode using two-line repetitions. The pinhole was set to 1 Airy unit. For lifetime fitting, a minimum photon count of 10,000 photons per decay curve was used and fitted with a biexponential decay model.

### Image analysis

Pollen tube tip width was determined by measuring the diameter in the apical region either on bright-field images or on fluorescence snapshots using Fiji/ImageJ version 1.54f^79^.

For the relative positions of actin filaments decorated by LifeAct–RFP and PIP5K2–EYFP, three stacks per cell were analysed. The LifeAct–RFP channel was analysed using the ImageJ skeleton plug-in^80^. End-points of each actin filament were collected in a region-of-interest manager, and the distance of each end-point from the PIP5K2–EYFP signal was manually determined. For LifeAct–RFP end-points fully overlapping with PIP5K2–EYFP, the distance was considered zero. Skeletons shorter than 3 pixels were considered as noise.

Actin dynamics were analysed as previously described^13,23^. In brief, *Z* image stacks were analysed over a 5-min time series with excitation of the YFP of the actin marker mTalin–YFP. Stacks were converted to *Z*-projections with maximum intensity using Fiji^79^. Backgrounds were subtracted using a rolling ball radius of 30 pixels. Due to the strong difference in fluorescence intensities between the pollen tube tip and the basal region of the tube, projections were divided into tip and body and analysed separately. Kymographs were generated from the tip region and analysed without bias for the length of the fluorescence signals using the machine learning program ilastik via segmentation and pixel classification^81^. The Euclidean diameter of the segments was extracted, and segments <5 pixels were excluded as background. Further quantitative analysis of the actin dynamics was carried out using Excel (version 2609 build 16.0.20430.20032) 64-bit. The lengths of the fluorescence signals of the kymographs of all evaluated pollen tubes were normalized against the width of the total length of the kymograph (in pixels). As a measure for actin dynamics, the mean lifetime of the actin signals was then calculated using the normalized values.

Actin occupancy was analysed by extracting the median plane of the *Z*-projections for actin dynamic analysis, and pixel size of both actin filaments and pollen tube tips was assessed using ilastik. The ratio of both values was used for calculating the occupancy in per cent. To analyse PM–actin attachment, the PM was marked in *Z*-projections with a segmented line, kymographs were generated using the reslice function in Fiji and mean lifetimes were calculated.

To examine the spatial correlation between ATM2 and RedStar–PH_PLC_, the first (most peripheral) optical section of the *Z*-stacks recorded by SD was analysed. Fluorescence channels were examined individually. Global colocalization was quantified using the Pearson’s coefficient function implemented in the Fiji plug-in JACoP^82^. For local colocalization, manually selected ATM2 domains were saved as regions of interest and analysed separately.

To visualize overlap between ATM2 and RedStar–PH_PLC_, surface sections acquired by SD were split into individual channels and converted into binary images by thresholding. The overlapping regions were identified using the image calculator, saved as regions of interest and then overlaid onto the original image for visualization.

For quantifying the area density of RedStar–PH_PLC_ signals at the pollen tube tip, the first slice of SD-acquired *Z*-projections was extracted. Pixel sizes were determined using ilastik and presented as a ratio to the membrane area analysed. To further quantify the distribution of the signals, the *Z*-projection was used to extract exact *x* and *y* coordinates using the Fiji plug-in TrackMate^83^.

Fluorescence intensity profiles were obtained from SD images. Using ImageJ, a segmented line was manually drawn along one side of the PM from the tip towards the shank. The selection was straightened (width 10 pixels) to generate a tip-to-shank membrane projection. Extracted fluorescence intensity profiles were normalized to a 0–1 intensity range. For the visualization of the spatial distribution of the mC–ATM2 signal, normalized intensity values ± s.e.m. were plotted. To obtain the dimension of mC–ATM2 domains, the spatial expanse of the signal was determined as a continuous membrane segment with normalized intensity ≥0.3. To distinguish between tip-focused and shank distribution, normalized intensities were calculated for the apical tip region (0–20 µm) and the adjacent shank region (20–40 µm) for each profile.

FRET/FLIM data were quantified on the basis of donor fluorescence lifetime. Donor-only samples were used to determine the reference lifetime, and donor–acceptor combinations were analysed for changes in donor lifetime. Fluorescence decay curves were fitted with a biexponential decay model in Leica LAS X software. Selected fitted decay parameters were extracted and compared between donor-only controls and donor–acceptor combinations.

The directional growth of pollen tubes was assessed by manually tracing vectors of pollen tube expansion in 3D projections of confocal imaging stacks. Vector maps were imported in the ImageJ directionality plug-in as previously described^63^. The mean directionality output for wild-type controls and the *atm2* *viii-b* double mutant is given as raw Fourier components and as fitted curves.

### Statistical evaluation

Statistical evaluation was performed as indicated, using ANOVA with Tukey’s post-hoc test performed using R (v4.61); or for pairwise comparisons by two-sample *t*-tests, assuming pooled variance with unknown equal standard deviation, performed using the free web-based service, statskingdom.de.

### In vitro lipid phosphorylation assay

The catalytic activity of recombinant PI4P 5-kinases was determined by their ability to phosphorylate PtdIns4P in the presence of [γ-^32^P]ATP, following established procedures^84^. In brief, the lipid substrate PtdIns4P (Avanti) was sonicated in the presence of Triton X-100 for 10 min in a sonicating bath. Afterwards, recombinant lipid kinase was mixed with or without ATM2, and 10 μCi [γ-^32^P]ATP was added. The reaction was started by combining the lipid and protein components. The reaction mix was incubated at room temperature for 15 min. Lipids were extracted using acidic phase separation. Radiolabelled lipid reaction products were separated by thin-layer chromatography and visualized by autoradiography. Incorporation of ^32^P was quantified by phosphorimaging using an Amersham Typhoon scanner (GE), BAS imaging screens (Fujifilm) and ImageQuant v10.2.

### Protein–lipid overlay assay

Protein–lipid overlay assays were performed using commercial PIP strips (Echelon Biosciences) containing 100 pmol of each immobilized lipid. The strips were blocked with 3% (w/v) milk powder in TBS buffer, washed three times with TBS and incubated for 45 min to 1 h with 0.5–2.5 mg ml^−1^ protein in 3% (w/v) BSA with gentle shaking. After three additional washes with TBS, the strips were incubated with primary antibody (mouse anti-penta-His, BSA-free, Qiagen, cat. no. 34670, diluted at 1:2,000) for 1 h at room temperature or overnight at 4 °C. After two washes with TBS-T and one wash with TBS, secondary antibody conjugated to alkaline phosphatase (goat anti-mouse IgG-AP, Sigma-Aldrich/Merck, cat. no. A9316, diluted at 1:30,000) was applied for 1 h. Unbound antibodies were removed by two washes with TBS-T, and membranes were equilibrated in AP buffer. Chromogenic detection was performed in 6 ml AP buffer with 21 μl NBT (75 mg ml^−1^ in 70% DMF) and 27 μl X-phosphate (50 mg ml^−1^ in 100% DMF). Reactions were stopped by the addition of ddH_2_O. All steps were carried out in the dark.

### Protein–lipid interaction analysis by MST

MST^85^ was used to quantify protein–lipid interactions^86^. Measurements were performed using a Monolith NT.115 instrument with MO.Control software (v.1.6.1; NanoTemper Technologies). Target proteins were covalently labelled with an sfGFP tag and a His_6_ tag for enrichment, and protein concentrations were adjusted to yield a constant fluorescence signal. Enriched recombinant sfGFP–His_6_ protein was used as a negative control. Protein dilutions were prepared in MST buffer (50 mM Tris/HCl pH 7.5, 150 mM NaCl, 0.05% (w/v) Tween-20). Liposomes composed of 80 mol% PtdCho and 20 mol% phosphoinositides were prepared as described previously^65^. After extrusion through a polycarbonate membrane (pore size 0.2 µm), 1.5 ml MST buffer without Tween-20 was added, followed by centrifugation at 20,000 *g* for 20 min at 22 °C. The sedimented liposomes were resuspended in MST buffer to 3 mM total lipid concentration. For MST experiments, a serial dilution of liposomes over 16 different concentrations was prepared in MST buffer from 3 mM down to 45.78 nM. Target protein concentration was maintained at ~40 nM. Five microlitres of target protein solution was mixed with 5 μl of ligand liposome dilutions, incubated for 10 min at room temperature and loaded in Monolith NT.115 standard capillaries (NanoTemper Technologies). The measurement was performed with 20% excitation power and medium MST-Power at an ambient temperature of 22 °C. The data were analysed with MO. Affinity Analysis (v.2.3., NanoTemper Technologies) using the signal from an MST-on time of 1.5 s.

### Yeast-two-hybrid analysis

Protein–protein interactions were assessed using split-ubiquitin membrane-based yeast-two-hybrid analysis (Dualmembrane Kit 3, Dualsystems Biotech) following established protocols^87^. In brief, bait constructs encoding *At*PIP5K2 or *At*PIP5K6 and prey with pollen-specific class VIII myosins or class XI myosins were co-transformed into the NMY51 yeast strain (Dualsystems Biotech). For controls, the bait protein was co-transformed with either NubI fused to the ER-resident protein Alg5 (positive control) or NubG fused to Alg5 (negative control). Interaction-dependent growth was tested on selective SD medium lacking leucine, tryptophan and histidine (SD−LWH).

### Accession numbers

The accession numbers for the genes in this study are At5g54280 for *ATM2*, At2g43790 for *MPK6*, LOC107765078 for Nt*Myo2-like isoform X2*, At1g77740 for *PIP5K2*, At3g07960 for *PIP5K6*, At1g47510 for *PTase11*, At5g03530 for *RABC2A*, At3g11730 for *RabD1*, At3g59770 for *SAC9*, At4g27370 for *VIII-B*, At1g17580 for *XI-1*, At5g43900 for *XI-2*, At1g04600 for *XI-A*, At1g08730 for *XI-C*, At1g54560 for *XI-E*, At4g28710 for *XI-H* and At4g33200 for *XI-I*.

### Reporting summary

Further information on research design is available in the Nature Portfolio Reporting Summary linked to this article.

## Supplementary information

Supplementary Information Supplementary Tables 1 and 2.

Reporting Summary

Peer Review File

## Source data

Source Data Uncropped Coomassie-stained SDS–PAGE gels for Figs. 3b and 6f.

## Data Availability

The data supporting the findings of this study are available in the Article and its Supplementary Information. All materials generated in this study are freely available upon request.
