# Supplementary material for: The class VIII myosin ATM2 stabilizes actin–plasma membrane contacts in tobacco pollen tubes by locally promoting PtdIns(4,5)P2 formation
Source: Nat Plants. 2026 Oct 16;12(10):2089–107. doi: 10.1038/s41477-026-02427-0 (PMC13680279; doi:10.1038/s41477-026-02427-0)
Supplement: Supplementary file 1 [file 41477_2026_2427_MOESM1_ESM.pdf]

nature plants

Supplementary information

https://doi.org/10.1038/s41477-026-02427-0

# The class VIII myosin ATM2 stabilizes actin–plasma membrane contacts in tobacco pollen tubes by locally promoting PtdIns(4,5)$P_2$ formation

In the format provided by the authors and unedited

# Supplementary Table 1. Raw data of FRET/FLIM analyses of EYFP-PIP5K2 vs. mC-ATM2$_{\Delta NT}$

| Construct | Date | Number | Path | | Channel | Region | Lifetime (τ) 2 ns | Mean τ, Intensity Weighted ns | Mean τ, Amplitude Weighted ns | χ² |
|---|---|---|---|---|---|---|---|---|---|---|
| K2-EYFP | 21.04.2026 | 1 | T1_K2_flim | PIP5K2-EYFP | HyD X 2 | Overall Decay | 2.920 | 2.889 | 2.654 | 1.308 |
| K2-EYFP | 21.04.2026 | 3 | T2_K2_flim | PIP5K2-EYFP | HyD X 2 | Overall Decay | 3.031 | 2.988 | 2.903 | 3.878 |
| K2-EYFP | 21.04.2026 | 5 | T3_k2_flim | PIP5K2-EYFP | HyD X 2 | Overall Decay | 2.979 | 2.934 | 2.756 | 2.457 |
| K2-EYFP | 21.04.2026 | 7 | T4_k2_flim | PIP5K2-EYFP | HyD X 2 | Overall Decay | 2.924 | 2.898 | 2.754 | 1.127 |
| ATM2dNT-mC_K2-EYFP | 21.04.2026 | 24 | T1_ATM2-mC_K2-flim | ATM2-mC_PIP5K2-EYFP | HyD X 2 | Overall Decay | 2.946 | 2.905 | 2.816 | 2.210 |
| ATM2dNT-mC_K2-EYFP | 21.04.2026 | 26 | T2_ATM2-mC_K2-flim | ATM2-mC_PIP5K2-EYFP | HyD X 2 | Overall Decay | 2.868 | 2.839 | 2.673 | 1.637 |
| ATM2dNT-mC_K2-EYFP | 21.04.2026 | 28 | T3_ATM2-mC_K2-flim | ATM2-mC_PIP5K2-EYFP | HyD X 2 | Overall Decay | 2.855 | 2.824 | 2.638 | 1.397 |
| ATM2dNT-mC_K2-EYFP | 21.04.2026 | 30 | T4_ATM2-mC_K2-flim | ATM2-mC_PIP5K2-EYFP | HyD X 2 | Overall Decay | 2.803 | 2.763 | 2.625 | 1.070 |
| ATM2dNT-mC_K2-EYFP | 21.04.2026 | 32 | T5_ATM2-mC-K2-flim | ATM2-mC_PIP5K2-EYFP | HyD X 2 | Overall Decay | 2.864 | 2.838 | 2.689 | 1.074 |
| ATM2dNT-mC_K2-EYFP | 21.04.2026 | 34 | T6_ATM2-mC-K2-flim | ATM2-mC_PIP5K2-EYFP | HyD X 2 | Overall Decay | 2.950 | 2.916 | 2.782 | 2.049 |
| ATM2dNT-mC_K2-EYFP | 21.04.2026 | 36 | T7_ATM2-mC_K2-flim | ATM2-mC_PIP5K2-EYFP | HyD X 2 | Overall Decay | 2.861 | 2.830 | 2.699 | 0.823 |
| ATM2dNT-mC_K2-EYFP | 21.04.2026 | 38 | T8_ATM2-mC_K2_flim | ATM2-mC_PIP5K2-EYFP | HyD X 2 | Overall Decay | 2.789 | 2.743 | 2.526 | 1.459 |
| ATM2dNT-mC_K2-EYFP | 21.04.2026 | 40 | T9_ATM2-mC_K2-flim | ATM2-mC_PIP5K2-EYFP | HyD X 2 | Overall Decay | 2.930 | 2.890 | 2.767 | 1.645 |

**Supplementary Table 2. Constructs used in this study**

| Gene of interest | Fluorophor/Tag | Backbone | Reference |
|---|---|---|---|
| mC | | *pEntry* | |
| EYFP | | *pEntry* | |
| $PH_{PLC}$ | RedStar | *pEntry* | 38 |
| NtMyo2-like | mC | *pEntry* | |
| ATM2 | EYFP/mC/CFP | *pEntry* | |
| $ATM2_{\Delta LB}$ | EYFP/mC | *pEntry* | |
| $ATM2_{CT}$ | mC | *pEntry* | |
| $ATM2_{\Delta NT}$ | mC | *pEntry* | |
| PIP5K2 | EYFP | *pEntry* | |
| PIP5K2 K470A | mC | *pEntry* | |
| $PIP5K2_{344-754}$ | EYFP | *pEntry* | |
| mTalin | YFP | *pEntry* | 57 |
| LifeAct | YFP/RFP | *pEntry* | 23, 55 |
| PTase11 | EYFP/mC | *pEntry* | 66 |
| SAC9 | mC | *pEntry* | 66 |
| LTI6b | mC | *pEntry* | 69 |
| PIP5K2 | | *pBT3C-Ost4* | 77 |
| XI-2 | | *pBT3C-Ost4* | |
| PIP5K6 | | *pBT3C-Ost4* | 71 |
| pAI-Alg5 | | *pPR3N* | |
| pDL2-Alg5 | | *pPR3N* | |
| ATM2 | | *pPR3N* | |
| VIII-B | | *pPR3N* | |
| XI-1 | | *pPR3N* | |
| XI-2 | | *pPR3N* | |
| XI-A | | *pPR3N* | |
| XI-C | | *pPR3N* | |
| XI-E | | *pPR3N* | |
| XI-H | | *pPR3N* | |
| XI-I | | *pPR3N* | |
| RabC2a | | *pPR3N* | |
| RabD1 | | *pPR3N* | |
| NtRhoGDI2 | | *pPR3N* | |
| MBP | $His_6$ | *pET28b* | |
| $ATM2_{CT\Delta LB}$ | $His_6$ | *pET28b* | |
| $ATM2_{CT}$ | $His_6$ | *pET28b* | |
| sfGFP | $His_6$ | *pET28b* | |
| sfGFP-$ATM2_{CT}$ | $His_6$ | *pET28b* | |
| sfGFP-$ATM2_{CT\Delta LB}$ | $His_6$ | *pET28b* | |
| PIP5K2 | MBP | *pMAL* | |
| VIII-$B_{CT}$ | $His_6$ | *pET28b* | |
| $ATM2_{CT}$ | $His_6$ | *pET28b* | |
| ROP2 | GST | *pGEX* | |
| $ROP2_{G15V}$ | GST | *pGEX* | |
| $ROP2_{T20N}$ | GST | *pGEX* | |
