# Supplementary material for: The class VIII myosin ATM2 stabilizes actin–plasma membrane contacts in tobacco pollen tubes by locally promoting PtdIns(4,5)P2 formation
Source: Nat Plants. 2026 Oct 16;12(10):2089–107. doi: 10.1038/s41477-026-02427-0 (PMC13680279; doi:10.1038/s41477-026-02427-0)
Supplement: Supplementary file 2 [file 41477_2026_2427_MOESM2_ESM.pdf]

nature portfolio

Corresponding author(s): Ingo Heilmann

Last updated by author(s): Aug 31, 2026

Reporting Summary

Nature Portfolio wishes to improve the reproducibility of the work that we publish. This form provides structure for consistency and transparency in reporting. For further information on Nature Portfolio policies, see our Editorial Policies and the Editorial Policy Checklist.

Statistics

For all statistical analyses, confirm that the following items are present in the figure legend, table legend, main text, or Methods section.

n/a	Confirmed	
☐	☒	The exact sample size (*n*) for each experimental group/condition, given as a discrete number and unit of measurement
☐	☒	A statement on whether measurements were taken from distinct samples or whether the same sample was measured repeatedly
☐	☒	The statistical test(s) used AND whether they are one- or two-sided<br>*Only common tests should be described solely by name; describe more complex techniques in the Methods section.*
☐	☒	A description of all covariates tested
☐	☒	A description of any assumptions or corrections, such as tests of normality and adjustment for multiple comparisons
☐	☒	A full description of the statistical parameters including central tendency (e.g. means) or other basic estimates (e.g. regression coefficient) AND variation (e.g. standard deviation) or associated estimates of uncertainty (e.g. confidence intervals)
☐	☒	For null hypothesis testing, the test statistic (e.g. *F*, *t*, *r*) with confidence intervals, effect sizes, degrees of freedom and *P* value noted<br>*Give P values as exact values whenever suitable.*
☒	☐	For Bayesian analysis, information on the choice of priors and Markov chain Monte Carlo settings
☒	☐	For hierarchical and complex designs, identification of the appropriate level for tests and full reporting of outcomes
☐	☒	Estimates of effect sizes (e.g. Cohen's *d*, Pearson's *r*), indicating how they were calculated

Our web collection on statistics for biologists contains articles on many of the points above.

Software and code

Policy information about availability of computer code

Data collection	n/a
Data analysis	n/a

For manuscripts utilizing custom algorithms or software that are central to the research but not yet described in published literature, software must be made available to editors and reviewers. We strongly encourage code deposition in a community repository (e.g. GitHub). See the Nature Portfolio guidelines for submitting code & software for further information.

Data

Policy information about availability of data

All manuscripts must include a data availability statement. This statement should provide the following information, where applicable:

- Accession codes, unique identifiers, or web links for publicly available datasets
- A description of any restrictions on data availability
- For clinical datasets or third party data, please ensure that the statement adheres to our policy

There are no restrictions on the availability of data included in this manuscript. All genetic resources are available on request.

Research involving human participants, their data, or biological material

Policy information about studies with human participants or human data. See also policy information about sex, gender (identity/presentation), and sexual orientation and race, ethnicity and racism.

Reporting on sex and gender	n/a
Reporting on race, ethnicity, or other socially relevant groupings	n/a
Population characteristics	n/a
Recruitment	n/a
Ethics oversight	n/a

Note that full information on the approval of the study protocol must also be provided in the manuscript.

Field-specific reporting

Please select the one below that is the best fit for your research. If you are not sure, read the appropriate sections before making your selection.

☒ Life sciences ☐ Behavioural & social sciences ☐ Ecological, evolutionary & environmental sciences

For a reference copy of the document with all sections, see nature.com/documents/nr-reporting-summary-flat.pdf

Life sciences study design

All studies must disclose on these points even when the disclosure is negative.

Sample size	No sample size calculations were performed before an experiment. We aimed for the largest possible dataset, given our experimental constraints. Our sample sizes are defined in part by the (low) experimental transformation efficiency. Within experiments, all transgenic cells that could be found and analyzed within the experimental time window were included in the analyses. Even though in consequence our sample sizes are low, the data show statistical relevance.
Data exclusions	No data were excluded from our report. To indicate possible outliers, we chose to represent data as box plots whenever possible, including all individual datapoints.
Replication	The data in our manuscript are strictly reproducible.
Randomization	We performed targeted experiments comparing the effects of defined variants of ATM2 or other relevant proteins. Therefore, randomization during sampling does not appear relevant for our study. Quantitative image analysis was performed by using non-biased approaches, such as calculating colocalization coefficients (Pearson's, Manders) that use all pixels in an entire region of interest, or by using machine-learning-based pattern-finding algorithms (Ilastik, Fiji trackmate) to minimize human bias.
Blinding	The experimental design was not blinded.

Reporting for specific materials, systems and methods

We require information from authors about some types of materials, experimental systems and methods used in many studies. Here, indicate whether each material, system or method listed is relevant to your study. If you are not sure if a list item applies to your research, read the appropriate section before selecting a response.

Materials & experimental systems

n/a	Involved in the study	
☒	☐	Antibodies
☒	☐	Eukaryotic cell lines
☒	☐	Palaeontology and archaeology
☒	☐	Animals and other organisms
☒	☐	Clinical data
☒	☐	Dual use research of concern
☐	☒	Plants

Methods

n/a	Involved in the study	
☒	☐	ChIP-seq
☒	☐	Flow cytometry
☒	☐	MRI-based neuroimaging

Plants

Seed stocks	All plant material used is specified in the Methods section.
Novel plant genotypes	There are no novel plant genotypes reported.
Authentication	Our study is concerned with the characterization of specific proteins, using the comparison of protein variants and their effects on in vitro pollen tube growth. All cDNA-constructs were verified by sequencing. In our experimental design no further genetic authentication is necessary, as every fluorescent cell observed represents an individual transformation event.
