# Supplementary material for: The class VIII myosin ATM2 stabilizes actin–plasma membrane contacts in tobacco pollen tubes by locally promoting PtdIns(4,5)P2 formation
Source: Nat Plants. 2026 Oct 16;12(10):2089–107. doi: 10.1038/s41477-026-02427-0 (PMC13680279; doi:10.1038/s41477-026-02427-0)
Supplement: Supplementary file 3 [file 41477_2026_2427_MOESM3_ESM.pdf]

nature portfolio

Peer Review File

The class VIII myosin ATM2 stabilizes actin-plasma membrane contacts in tobacco pollen tubes by locally promoting PtdIns(4,5)P2 formation

Corresponding Author: Professor Ingo Heilmann

This file contains all reviewer reports in order by version, followed by all author rebuttals in order by version.

Version 0:

Reviewer comments:

Reviewer #1

(Comments for the Author)
This manuscript presents a comprehensive follow-up study on the role of the Arabidopsis class VIII myosin ATM2 in linking actin cytoskeleton dynamics to plasma membrane nano-organisation. Building on the group's previous work (Fratini et al.; Kastner et al.), the authors now explore how ATM2 modulates phosphatidylinositol 4,5-bisphosphate nano-domain formation and, in turn, influences actin dynamics and ROP signalling at the pollen tube tip.
The study is well-structured, and the experimental approaches are generally sound and carefully executed. The manuscript is clearly written, the data are of high quality, and the authors avoid overinterpreting their results. The work convincingly demonstrates that ATM2 binds both actin and PtdIns(4,5)P_2, that this interaction is required for its plasma membrane localisation and actin-stabilising activity, and that ATM2 can enhance PtdIns(4,5)P_2 abundance and PIP5K2 catalytic activity, thereby contributing to a positive feedback loop promoting local actin stabilisation.
Overall, this work provides valuable insights into the molecular mechanisms linking the actin cytoskeleton with membrane lipid organisation and reinforces the central role of PtdIns(4,5)P_2 nano-domains in polarised tip growth. However, while the study is solid and the data primarily support the conclusions, I have several points that should be addressed :

1. While the experiments are carefully performed and the conclusions well supported, the overall conceptual advance beyond the group's previous work (Fratini et al., Kastner et al.) is somewhat limited. Much of the study confirms earlier findings, with the main new elements being (i) the cellular characterisation of ATM2's effect on a PtdIns(4,5)P_2 biosensor and (ii) the interaction between ATM2 and RhoGDI2, an effector of the ROP pathway. These are significant additions but do not fully resolve the physiological significance of ATM2 function, particularly regarding the ROP signalling pathway. The study's impact would be significantly enhanced if the authors complement the transient overexpression experiments with genetic approaches. For example, CRISPR–Cas–generated knockout lines of ATM2 and/or RhoGDI2 would allow testing whether these proteins are indeed required for PtdIns(4,5)P_2 patterning, actin organisation, and ROP signalling in vivo.

2. A significant concern is using GFP–mTalin as the primary reporter for actin dynamics. This probe is known to interfere with actin organisation and dynamics, as reported in Ketelaar et al. (Plant Physiol. 2004). The authors should confirm their main findings using alternative actin reporters that are less perturbative, such as Lifeact or an already described actin-binding domain (ABD). These tools would provide more confidence in the observed effects of ATM2 (and truncated versions) on actin dynamics.

3. In an elegant set of experiments, the authors tested whether ATM2 binding to PtdIns(4,5)P_2 is consistent with the effects of PIP5K2 (Fratini et al., 2021) or PTase11 on ATM2 function in controlling actin dynamics (cf. Fig. 3). They further explored whether a ~3-fold difference in binding affinities to PtdIns(4,5)P_2 versus PtdIns4P could provide a molecular basis for the PtdIns(4,5)P_2-dependency of ATM2's regulatory role on actin. The data from both the in vivo observation and in vitro assays are consistent with a promoting effect of ATM2 on PtdIns(4,5)P_2 formation in the plasma membrane.
However, the authors did not investigate the contribution of PI4P, although tools such as SAC1 overexpression are available and could be readily employed. Such experiments would help determine whether the observed effects on the PtdIns(4,5)P_2 biosensor are specific or if other phosphoinositides are also involved. This is particularly relevant in light of previous findings showing that ROP signaling is also regulated by the distribution of phosphatidylserine (PS) at the plasma membrane (Platre et al., Science, 2019). Addressing lipid specificity experimentally — or at least discussing this limitation more thoroughly —

would significantly strengthen the mechanistic conclusions of the manuscript.

4. The manuscript refers to overexpression of PTPase11 or SAC9 as "artificial" perturbations of PtdIns(4,5)P_2, while transient expression of PIP5K2 is presented as a means to assess physiological PtdIns(4,5)P_2 function. This distinction is unclear and inconsistent, as overexpression of PIP5K2 is a strong perturbation of PtdIns(4,5)P_2 homeostasis.

Reviewer #2

(Comments for the Author)
In this manuscript, the authors studied how the Arabidopsis class VIII-myosin ATM2 regulates the accumulation/repartition of PtdIns(4,5)P2 and actin dynamics in pollen tubes, which has an impact on the morphology of these later. The authors performed an interesting multidisciplinary approach combining pollen tube swelling assay, cell biology, biochemistry, protein-protein interaction tests, among others. They showed that the overexpression of ATM2 stabilized actin and induces tip swelling in pollen tube cells. ATM2-effects on actin and pollen tube swelling required PtdIns(4,5)P2, although in absence of PtdIns(4,5)P2, ATM2 is properly localized to the plasma membrane. ATM2 overexpression stimulated PtdIns(4,5)P2 abundance in the pollen tube as revealed using a PtdIns(4,5)P2-biosensor. Interestingly, ATM2 interacted with the PI4P 5-kinase, PIP5K2 and could stimulate its activity by an unknown mechanism. The author proposed that ATM2 locally promoted the accumulation of PtdIns(4,5)P2, which self-reinforces ATM2-recruitment and control actin-dynamics and pollen tube morphology.
The work presented in the manuscript is original and interesting and the manuscript is well written. Phenotypical analysis achieved through pollen tube swelling assay as well as biochemistry experiments are convincing. However, I have several caution concerning some cell biology approaches and their interpretation as detailed below. In addition, I believe that some protein-protein interactions need to be validated in plant cells and not only in a heterologous system, yeast. An important question that is note discussed is how the depletion of PtdIns(4,5)P2 interferes with ATM2 function, if it does not disturb its plasma membrane localization. I think that saying that ATM2 modulate membrane lipid nano-organization is not supported by experimental data. Indeed, as detailed below, although the authors show that ATM2 influence PtdIns(4,5)P2 repartition in pollen tubes, the nanoscale organization of the lipid is not affected. In summary, I think that the authors described an interesting process but that needs to be strengthened by some additional experiments and controls as well as by adding additional explanations, as proposed below.

Major comments

Line 29: The title "The class VIII myosin ATM2 stabilizes actin-plasma membrane contacts by locally promoting PtdIns(4,5)P2 nanodomain formation in tobacco pollen tubes" is not appropriated. Indeed, although the authors show that ATM2 overexpression expand the plasma membrane region where the fluorescent PtdIns(4,5)P2-biosensor is detected in pollen tube (Figure 5B), they also show that ATM2 does not modify the nanodomain density of the PtdIns(4,5)P2-biosensor. Therefore, the notion of nanodomain should be retired from the title. The same comment can be done in Figure 7, where the authors indicated in the scheme "locally increased PtdIns(4,5)P2 formation (nanodomain)". This work does not provide evidence that the local concentration of PtdIns(4,5)P2 in nanodomains increases in presence of ATM2.

Line 42, summary. The authors say: "ATM2 colocalized and interacted with the PI4P 5-kinase, PIP5K2…"
I did not see any co-localisation experiment between ATM2 and PIP5K2 in the manuscript. The co-localization experiment provided is between ATM2 and the sensor RedStar-PHPLC (Figure 5A).

Figure 1A: Please specify which promoter was used to express EYFP-ATM2. If a constitutive promoter was used, why is pollen tube tip swelling not observed as in Figure 2B where ATM2 was over-expressed with pLat52 promoter? This needs some precisions.

Figure 1B: PIP5K2-EYFP is supposed to be localized in the plasma membrane but, as especially observed in the detail view (white box), a PIP5K2-EYFP signal is also detected under the plasma membrane. The authors should comment this localization.

Figure 1B: Qualitative data are provided, but please provide also quantitative information by measuring the proportion of actin filaments associated with PIP5K2-EYFP domains.

Figure 1C:
In this figure, I do not understand how the authors can state that when mC-ATM2 was co-expressed with mTalin-YFP marker for actin, they observed enhanced actin signals at the membrane-proximal ends of actin filaments intersecting with the plasma membrane. How was it quantified? Did they compare with the situation where mTalin-YFP was expressed alone, without mC-ATM2?
To facilitate the interpretation of the data, please provide all the channels and not only the merge pictures.

Figure 2B: The authors propose a locally increased mTalin-YFP signal at the plasma membrane in presence of ATM2. How do the authors reconciliate the homogenous distribution of ATM2 in the plasma membrane with a patterning of actin in hot

spots in the plasma membrane?

Figure 3A: The fact that PtdIns(4,5)P2 depletion using PTase11 absolutely does not disturb ATM2 plasma membrane localization is intriguing, especially because the authors then show that ATM2 preferentially binds to PtdIns(4,5)P2 (Figure 4). In the discussion the authors propose that PtdIns4P could ensure the anchoring of ATM2 in the plasma membrane. I think it needs some precisions.

As previously mentioned, how does the depletion of PtdIns(4,5)P2 interfere with ATM2 function, if it does not disturb its plasma membrane localization? The authors should propose some possible mechanisms.

Figure 3: Please provide a control showing that PtdIns(4,5)P2 depletion indeed occurs in this system by expressing PTase11. It can be easily monitored using the PtdIns(4,5)P_2 biosensor, RedStar-PHPLC.

Figure 3A. Compared to figures 1 and 2, some images seem to be post-processed such as CFP-ATM2 (top and bottom) and mCherry (top). Please provide other images.

Figure 3. While mC-Control is indicated in the graphs (C), the corresponding images are not indicated in (A), please provide them.

Figure 4A. Please provide SDS-PAGE + coomassie blue staining of the purified proteins: MBP-His6, ATM2CT-His6 and ATM2CTΔLB-His6.

Figure 4. Even if I fully understand when authors say that "a drawback of the lipid overlay assay is that lipids are presented in very high abundance but not in a membraneous formulation, and it may be difficult to assess quantitative differences in lipid", I am a little bite surprise that the two lipids displaying the highest affinity/signal for ATM2CT-His6 (PtdIns3P and PtdIns5P) (Figure 4A) were not analyzed by microscale thermophoresis. Therefore, I think it is a little bite overstated to say line 262 that "ATM2 displays a binding preference for PtdIns(4,5)P2 in vitro" since the only comparison is done with PtdIns4P. At least, please modify the sentence.

Figure 5A. In this co-localization analysis, controls are missing. As a negative control, please provide data with a lipid sensor or a plasma membrane protein that does not colocalize with ATM2 in nanodomains. A positive control for colocalization in nanodomains, for instance by co-expressing EYFP-ATM2 and mCherry-ATM2, would be also important.

In figure 5A, EYFP-ATM2 protein seems to organize, at least partially, in plasma membrane nanodomains, whereas in figure 1A, the same protein displays a homogenous distribution in the plasma membrane. Since in both analyses spinning disc microscopy was used and z-projections are presented, please comment this inconsistency.

Figure 5B. The nanodomain density of RedStar-PHPLC is presented but without the corresponding microscopy images. Please add images showing RedStar-PHPLC nanodomains without ATM2, with ATM2 and with ATM2deltaLB.

Figure 5C: Are the authors sure that myosins XI are correctly produced in yeast since no interaction was detected in this test and no positive control of the interactions between a given myosin XI and another protein is provided?

In figure 5, the interaction between ATM2 and PIP5K2 proteins was only investigated in a heterologous system, yeast. The same remark can be done concerning the interaction tests presented in Figure 6A. Focusing on ATM2/PIP5K2, NtRhoGDI2/PIP5K2 and NtRhoGDI2/ATM2 couples, the authors should provide evidences for protein-protein interactions in plant cells by the technic of their choice (co-IP, FRET…).

Figure 5D: It is difficult for the reader to easily interpret this panel. Please indicate in the legend what the black and white arrowheads correspond to. What does the intermediate band without any arrowhead labelling correspond to?

Figure 6B and C: The authors claim that the expression of NtRhoGDI2-EYFP influenced the plasma membrane distribution of mC-ATM2 along the pollen tube. However, I think that the microscopy images in 6B do not support that. Indeed, I cannot observe that mC-ATM2 distribution is more restricted to the tip when co-expressed with NtRhoGDI2-EYFP. What is seen is that mC-ATM2 signal is maybe more intense at the tip in presence of NtRhoGDI2-EYFP, but not more confined.
In addition, according to the spatial distribution of mC-ATM2 signal in panel C, a peak of fluorescence should be observed at around 60 um from the tip in presence of unfused EYFP. This peak cannot be observed in the image provided in panel B. The authors need to clarify these two points.

Concerning the data from Figure 6, it remains unclear how NtRhoGDI2-EYFP abolishes the tip swelling induced by ATM2. Please provide hypotheses.

In several sentences of the manuscript, the authors say that ATM2 stabilizes actin-dynamics. To my opinion it sounds weird because "dynamics" and "stabilization" are antinomic.

Minor comments

The title of Figure 1 is "The PtdIns(4,5)P2 biosensor RedStar-PHPLC and EYFP-ATM2 occupy overlapping subapical

plasma membrane domains of tobacco pollen tubes, coinciding with actin-plasma membrane contacts”. I suggest to replace “domains” by “zones” to avoid confusion with nanodomains.

Figure 4 C and D. To facilitate the comparison between the lipid binding properties of sfGFP-ATM2CT and sfGFP-tag alone, it would be preferable to present all the data on the same graph.

I think it could be interesting to cite in the manuscript the work of Wang et al. 2024, PNAS, on myosin XI nanodomain organization.

Version 1:

Reviewer comments:

Reviewer #1

(Comments for the Author)
The reviewer previously questioned the specificity of ATM2 binding to PtdIns(4,5)P_2. The authors’ new data show that ATM2 binds anionic lipids non-specifically, remains associated with the plasma membrane after PtdIns(4,5)P_2 depletion, yet loses its actin regulatory function. The reviewer advises removing claims of PtdIns(4,5)P_2 specificity from the title, abstract, and figure legends. For example, the abstract sentence “ATM2 binds both actin and phosphatidylinositol 4,5-bisphosphate (PtdIns(4,5)P_2), a lipid required for ATM2-PM attachment” could be revised to: “ATM2 binds both actin and anionic lipids, including PtdIns(4,5)P_2, which is required for ATM2-PM attachment.”

The authors should consider adding a section on "limitations of the study" in the Discussion, as not all the requested experiments were done, mainly because of time and technical problems.

I could not find information about the lines/constructs used in this study. A recap table in the Materials and Methods would be useful. For example, the constructs mTalin:YFP and YFP-mTalin (Supplemental Figure 3 legend and pannels) are mentioned, but there is no information about the differences between them.

In general, I would prefer graphs to display individual data points rather than only mean values. It would also be more reader-friendly to consistently include n= (sample size) and N= (number of experiments) on all graphs. This information is sometimes in the legend but not always.

Specific Comments
Line 942: Spelling mistake – “ceels” → “cells”. Please indicate the number of replicates.
Figure 1F: Spelling mistake – “ceels” → “cells”. Please indicate the number of replicates. The authors should justify the sample size (n=4 cells) and provide the raw data in the supplemental material, as the sample size is very small.
Figures 2A and 2D: Scale bars are missing.
Figure 2F: N=?
Figure 4: There is substantial variation in sample sizes for the mC-control (20–29 cells) vs. ATM2 (only 6 cells). Please explain this discrepancy and ensure it does not affect the statistical analysis. Given the small sample size (n=6), I encourage the authors to show the individual data points in the supplemental material.
Figure 4E: N=?
Figure 4F: n=? N=?
Figure 5: N=?
Figure labels: The authors refer to the RedStar-PH biosensor inconsistently as “red channel” or “mC”. This is confusing; please clarify and standardize the labels throughout the manuscript.
Figure 6B: “detail” is unclear. Please add a scale bar and clarify the panel.
Figure 6F: Please show the full blot in the supplemental material.
Figure 6C: N=?
Figure 6E: n=? N=?
Supplemental Figure 1D: n=? N=?
Supplemental Figure 2B: N=?
Supplemental Figure 4B: spelling mistake “Data are from three independen experiments”
Supplemental Figure 5B: N=?
Supplemental Figure 5: Only 5 pollen tubes were analyzed for Ptase11-YFP. Given the small sample size, please display the images. N=? n=?
Supplemental Figure 6A: Only 6 cells were analyzed for CFP-ATM2. Please display the images, as the sample size is very small.

Model: Since the paper no longer focuses on RhoGDI, should the authors retain it in the scheme?

Reviewer #2

(Comments for the Author)
In this revised manuscript, the authors performed numerous additional experiments and carefully answered to all my comments. To my opinion, this work is very interesting and is now complete. Below, I have just few comments on the additional experiments that were provided in this revision. Although additional experiments are not required, I suggest the authors to rearrange some figures, measure PCC values for the colocalization test in Figure 1E, and justify with more details in the manuscript the use of ATM2 variant lacking part of its N-terminus.

Line 42, summary. The authors say: "ATM2 colocalized and interacted with the PI4P 5-kinase, PIP5K2…"
I did not see any co-localisation experiment between ATM2 and PIP5K2 in the manuscript. The co-localization experiment provided is between ATM2 and the sensor RedStar-PHPLC (Figure 5A).

Reply:
We agree with the reviewer that we had not shown colocalization and that our statement had been misleading. We have now performed additional experiments to address the spatial relations of coexpressed ATM2 and PIP5K2. Coexpression of PIP5K2 with full-length ATM2 might be lethal. Therefore, we analyzed the coexpression of PIP5K2 with the slightly shorter ATM2deltaNT variant, and we observed very similar PM patterns of ATM2deltaNT and PIP5K2 by spinning disc microscopy, presented in Figure 1 with the notice that the shorter ATM2 protein variant was used.

Reviewer 2. In this co-localization analysis (Figure 1E), I understand the possible limit of working with the full length ATM2 protein but are the authors sure that ATM2 and ATM2deltaNT display the same subcellular localization? I read in their response that this truncated ATM2 protein was used in previous studies, but I think that this choice needs to be at least briefly explained in the manuscript.
In Figure 1E, it seems that ATM2 and PIP5K2 are co-localized but I suggest to the authors to quantify this colocalization to strengthen the conclusion, for instance by measuring the Pearson's correlation coefficient. A negative control for colocalization, for instance ATM2 with an unrelated protein, would have been beneficial, but it is probably not obligatory given the rather particular localization of the two proteins of interest.

Figure 1C:
In this figure, I do not understand how the authors can state that when mC-ATM2 was co-expressed with mTalin-YFP marker for actin, they observed enhanced actin signals at the membrane-proximal ends of actin filaments intersecting with the plasma membrane. How was it quantified? Did they compare with the situation where mTalin-YFP was expressed alone, without mC-ATM2? To facilitate the interpretation of the data, please provide all the channels and not only the merge pictures.

Reply:
We appreciate this comment and have altered the figure to now represent the individual channels as well as the merged overlays. In the separate mTalin-YFP channel, an enhanced signal intensitiy at the intersection with the PM can now clearly seen. The intensitiy of the actin marker fluorescence was now quantified along the filaments and showed a marked increase when the filaments intersected with the mC-ATM2-decorated PM (also quantified). The pattern was observed upon expression of Arabidopsis ATM2 as well as for the tobacco homolog, NtMyo2-like. The data are shown as part of the revised Figure 2. While the data clearly show enhanced actin marker fluorescence at the PM, this pattern can not easily be compared to a control expressing the actin marker with mC only, as with no ATM2 present, the filaments in the cell apex cannot be seen or imaged. Therefore, we regret that this requested control cannot be provided.

Reviewer 2. Without presenting a control where mTalin-YFP was expressed alone, without mC-ATM2, I believe it is difficult to conclude on a putative effect of ATM2 solely using panel A (Figure 2). However, the authors provide in Figure 2 panel D pictures where mTalin-YFP was co-expressed with mC, which is to my opinion the required control for panel A, even if quantification is not possible for this control. Therefore, I suggest to present this control earlier in the manuscript or at least to cite panel D when interpreting panel A.

In figure 5A, EYFP-ATM2 protein seems to organize, at least partially, in plasma membrane nanodomains, whereas in figure 1A, the same protein displays a homogenous distribution in the plasma membrane. Since in both analyses spinning disc microscopy was used and z-projections are presented, please comment this inconsistency.

Reply:
We appreciate the comment but are a bit confused, as for our eyes the images in Figure 1 A show a quite inhomogenous PM pattern for the EYFP-ATM2 marker. This inhomogenous pattern was always observed when fluorescent fusions of ATM2 were imaged by spinning disc microscopy. We are now providing an added detail view of the EYFP-ATM2deltaNT marker imaged by spinning disc microscopy in Figure 1 E to illustrate the PM pattern in relation to that of PIP5K2-mC.

Reviewer 2. I agree that in Figure 1B (new figure), the localization of EYFP-ATM2 is inhomogenous, as stated by the authors, however it is difficult to see nanoclusters to my opinion, contrary for instance to what is observed with RedStar-PHPLC that clearly arranges in nanodomains. That is why I thought that the picture with EYFP-ATM2 was probably not representative. In the new figure 1E, EYFP-ATM2ΔNT is also not homogeneously distributed, but again, I think it is not localized in clear nanodomains. I just wanted to make the authors aware of these aspects.

In figure 5, the interaction between ATM2 and PIP5K2 proteins was only investigated in a heterologous system, yeast. The same remark can be done concerning the interaction tests presented in Figure 6A. Focusing on ATM2/PIP5K2, NtRhoGDI2/PIP5K2 and NtRhoGDI2/ATM2 couples, the authors should provide evidences for protein-protein interactions in plant cells by the technic of their choice (co-IP, FRET…).

Reply:
The in planta interaction at the center of our study is that of ATM2 with PIP5K2, and we performed FRET/FLIM experiments to verify the interaction in vivo in pollen tube cells, as suggested. We have been able to record FRET/FLIM data for ATM2deltaNT vs. PIP5K2 and observed a significant reduction of the CFP-ATM2deltaNT donor fluorescence lifetime in the presence of PIP5K2-EYFP, but not for the EYFP control. The interaction data are presented in Figure 1 noting that the shorter ATM2 variant was used.

Reviewer 2. Figure 1F. Thank you for performing this very important experiment. I suggest to the authors to indicate in their graph the fluorescence lifetime of the donor alone (CFP-ATM2ΔNT), which is usually presented in FRET-FLIM analyses. It will allow to compare the fluorescence lifetime in presence of EYFP and PIP5K2-EYFP. I do not understand what is the meaning/interest of the values Mean τ (int.) and Mean τ (amp.). For Mean τ (amp.), there is no statistical difference between the negative control EYFP and PIP5K2-EYFP.
The results appear conclusive, showing statistical differences between the negative control and the interaction test (CFP-ATM2ΔNT+ PIP5K2-EYFP), however, I would still like to point out that the observed decrease in the tau value for CFP-ATM2ΔNT+ PIP5K2-EYFP compared to the negative control is slight.
I would suggest to the authors to add a panel showing the FRET-FLIM acquisition with the fluorescence lifetime indicated by a pseudo color code. Indeed, this information is interesting for the readers since one of the advantages of FRET-FLIM is to “visualize” interaction at the subcellular level. This panel could replace the table with fluorescence lifetime values that is, to my opinion, not very informative compared to the graph that is presented in Figure 1F.

Open Access This Peer Review File is licensed under a Creative Commons Attribution 4.0 International License, which permits use, sharing, adaptation, distribution and reproduction in any medium or format, as long as you give appropriate credit to the original author(s) and the source, provide a link to the Creative Commons license, and indicate if changes were made.
In cases where reviewers are anonymous, credit should be given to 'Anonymous Referee' and the source.
The images or other third party material in this Peer Review File are included in the article’s Creative Commons license, unless indicated otherwise in a credit line to the material. If material is not included in the article’s Creative Commons license and your intended use is not permitted by statutory regulation or exceeds the permitted use, you will need to obtain permission directly from the copyright holder.
To view a copy of this license, visit https://creativecommons.org/licenses/by/4.0/

Response to Referees

(all original content of the decision letter is repeated; responses are highlighted in grey)

22nd January 2026

Dear Professor Heilmann,

First of all, please apologize the delayed handling of your manuscript. Your Article, "The class VIII myosin ATM2 stabilizes actin-plasma membrane contacts by locally promoting PtdIns(4,5)P2 nanodomain formation in tobacco pollen tubes" has now been seen by two referees. You will see from their comments below that while they find your work of interest, some important points are raised. We are interested in the possibility of publishing your study in Nature Plants, but would like to consider your response to these concerns in the form of a revised manuscript before we make a final decision on publication.

As you can read below, both reviewers think that the findings are interesting and generally well supported by the data, but both reviewers think that the impact of the findings could be further strengthened by additional experiments. In our opinion the main points are:

1. Reviewer #1 thinks that the study's impact would be significantly enhanced if the transient overexpression experiments were complemented with genetic approaches, and if the specificity of ATM2 for the lipid PtdIns(4,5)P2 were assessed.

Reply:
We thank the reviewer for these suggestions and have performed additional experiments to address these points.

For the loss-of-function experiments, we obtained an Arabidopsis mutant (Liu et al., Plant Commun 7, 101616, 2026, doi 10.1016/j.xplc.2025.101616) that is reduced in expression of both pollen-specific expressed class VIII-myosins, ATM2 and VIII-B. The mutant pollen tubes displayed interesting germination behavior and a growth defect that are consistent with destabilized actin, as is also consistent with our ATM2 overexpression data (where actin was over-stabilized). The additional data (new Extended Data Figure 4), thus, provide complementary evidence in support of our story.

We also performed additional analyses to test the binding of a purified recombinant ATM2-fragment to various additional lipids. The data indicate that there is no clear binding preference and that various anionic lipids bind with similar affinities, including PtdIns-bisphosphates, PtdIns-monophosphates, PtdOH, or PtdSer. This observation is in line with our lipid-overlay data and supports the notion that ATM2 function does not require preferential binding of ATM2 to any one particular lipid. Instead, we propose that the stimulation of PtdIns(4,5)P2 production by ATM2 results in a localized change in membrane lipid composition that facilitates actin-plasma membrane (PM) attachment and manifests as a "PtdIns(4,5)P2-specific" effect. The data are included in the updated Figure 3.

2. Reviewer #1 thinks that the main conclusions based on the GFP–mTalin actin reporter should be complemented with alternative actin reporters that are less perturbative, and reviewer #2 thinks that protein-protein interactions need to be validated in plant cells.

Reply:
We thank the reviewers for these suggestion and have performed the requested experiments.

We agree that more than one actin marker provides a better view of potential marker effects on actin dynamics. We have repeated all actin dynamics measurement for the various truncations of ATM2 by using the less-perturbative marker, LifeAct, and we obtained similar effects on actin dynamics as when we had used the mTalin marker before: The expression of ATM2 also stabilized LifeAct-decorated actin, and lipid binding was required for this effect. While the overall patterns were thus similar, when using LifeAct mostly PM-proximal actin at the cell periphery was stabilized, whereas the use of mTalin additionally showed some stabilized actin also in the cell center. These differences are likely a consequence of mTalin being a larger and more "perturbative" actin-binding marker, as indicated by the reviewers. We are now citing the sugested reference (Ketelaar et al., Plant Physiol. 136, 3990-3998,2004, doi 10.1104/pp.104.050799) for the effect. Overall, we find our data stronger by the addition of the LifeAct experiments, as the effects of ATM2 can now be reported based on two independent actin markers. We additionally performed experiments to assess the cell morphologies arising from expressing ATM2 or its various truncations, either in the presence of mTalin or LifeAct as actin markers, again with similar results. The data are included in the Extended Data Figure 3.

The *in planta* interaction at the center of our study is that of ATM2 with PIP5K2, and we performed FRET/FLIM analysis to verify this interaction *in vivo* in pollen tube cells, as suggested. The FRET/FLIM analysis was performed with an ATM2 variant lacking part of its N-terminus (here termed ATM2deltaNT), as was used in previous studies (Kastner et al., Biochimie 203, 65-76,2022, doi 10.1016/j.biochi.2022.10.002). The FRET/FLIM data for ATM2deltaNT vs. PIP5K2 indicate a significant reduction of the CFP-ATM2deltaNT donor fluorescence lifetime in the presence of PIP5K2-EYFP, but not for the EYFP control. The interaction data are presented in Figure 1, noting that the ATM2deltaNT protein variant was used.

Two further protein-protein interactions presented in our initial submission (ATM2 vs. NtRhoGDI2 and PIP5K2 vs. NtRhoGDI2) have not been explored in more detail, as we have mostly removed the respective mention from the text. The respective yeast-two-hybrid experiments are still shown as part of Extended Data Fig. 1, but are now only used as additional evidence for the proposed model (Fig. 7). We hope that our narrower focus with more depth will meet the favour of the reviewers.

3. Reviewer #2 states that it should be clarified how the depletion of PtdIns(4,5)P2 interferes with ATM2 function if it does not disturb its plasma membrane localization.

Reply:
We appreciate this comment, which is especially relevant as our newly obtained data show that the lipid-binding region of ATM2 did not display specific binding to any particular anionic lipid *in vitro* (updated new Fig. 3). The retained PM-association of ATM2 upon PtdIns(4,5)P2-depletion by two different specific lipid phosphatases is consistent with our additional *in vitro* lipid binding data (see updated Figure 3) indicating that other lipids also bind ATM2, thereby likely retaining it at the PM. We consider it a key finding of our study that depleting PtdIns(4,5)P2 did not result in reduced PM-association of ATM2 but nonetheless abolished the effect of ATM2 on actin dynamics. To further support the notion that PtdIns(4,5)P2 formation by PIP5K2 is required for the effect of ATM2 on actin, we performed additional experiments expressing the catalytically inactive variant PIP5K2 K470A, and we observed a dominant negative effect over ATM2 (included in the new Figure 4). Our combined data converge on a model (Figure 7) where ATM2 promotes PtdIns(4,5)P2-formation by its interaction partner, PIP5K2. In this model, ATM2 associates with the PM by binding to anionic lipids (Figure 7 A). A regulatory effect ensues when ATM2 encounters PIP5K2 (Figure 7 B). The activation of PIP5K2 by ATM2 locally promotes PtdIns(4,5)P2-formation, thereby aiding the recruitment of PM-associating factors controlling actin dynamics as well as recruiting more ATM2, resulting in a self-amplifying cycle of PtdIns(4,5)P2 production at sites of actin-PM-attachment. At these sites, PtdIns(4,5)P2 might now be the defining element of the PM, focusing the recruitment of various proteins to the PM and resulting in "PtdIns(4,5)P2-dependent" actin stabilization. We have expanded the text and the model figure to better illustrate this logic.

All referees also raise some other points that should be addressed. As usual, please do not hesitate to get in touch if you would like to discuss these issues further.

We therefore invite you to revise your manuscript taking into account all reviewer and editor comments. Please highlight all changes in the manuscript text file, preferably in Microsoft Word format.

We are committed to providing a fair and constructive peer-review process. Do not hesitate to contact us if there are specific requests from the reviewers that you believe are technically impossible or unlikely to yield a meaningful outcome.

When revising your manuscript please:

* Include a "Response to referees" document detailing, point-by-point, how you addressed each referee comment. If no action was taken to address a point, you must provide a compelling argument. This response will be sent back to the referees along with the revised manuscript.

* If you have not done so already please begin to revise your manuscript so that it conforms to our Article format instructions at http://www.nature.com/nplants/info/final-submission. Refer also to any guidelines provided in this letter.

* Include a revised version of any required reporting checklist. It will be available to referees (and, potentially, statisticians) to aid in their evaluation if the manuscript goes back for peer review. A revised checklist is essential for re-review of the paper.

* Pay close attention to our href="https://www.nature.com/nature-portfolio/editorial-policies/image-integrity">Digital Image Integrity Guidelines. and to the following points. Please ensure:
-- that unprocessed scans are clearly labelled and match the gels and western blots presented in figures.
-- that control panels for gels and western blots are appropriately described as loading on sample processing controls
-- that all images in the paper are checked for duplication of panels and for splicing of gel lanes.
-- that you retain unprocessed data and metadata files after publication, ideally archiving data in perpetuity. These may be requested during the peer review and production process or after publication if any issues arise.

EXTENDED DATA FIGURES

When re-submitting your manuscript, please ensure that any supplementary figures and tables that are crucial to the manuscript's conclusions are converted into Extended Data figures and tables to increase visibility of these data. Extended Data figures and tables are online-only (present in the online PDF and full-text HTML versions of the paper), peer-reviewed display items that provide essential background to the article but are not included in the main article due to space constraints. A maximum of ten Extended Data display items (figures and tables) is permitted.

Nature Plants strongly supports public availability of data and are therefore keen that the data used in your paper is placed in an appropriate public data repository. Alternatively, if this is not possible, you may present the data as Extended Data or Supplementary Information. If data can only be shared on request, please explain why in your Data Availability Statement, and also in the correspondence with your editor. Please note that for some data types, deposition in a public repository is mandatory.

Please use the link below to submit your revised manuscript and related files:

https://mts-nplants.nature.com/cgi-bin/main.plex?el=A7Ce6BaD1A6CfN7J1A9ftdBfbnlTppqvjsjOPqTRhMwZ

Note: This URL links to your confidential home page and associated information about manuscripts you may have submitted, or that you are reviewing for us. If you wish to forward this email to co-authors, please delete the link to your homepage.

We hope to receive your revised manuscript within three to four months, however, this is not a strict deadline. If you cannot send it within this time, please let us know. We will be happy to consider your revision so long as nothing similar has been accepted for publication at Nature Plants or published elsewhere.

We look forward to seeing the revised manuscript and thank you for the opportunity to review your work.

Sincerely,

Raphael Trösch
Editor
Nature Plants

Nature Plants is committed to improving transparency in authorship. As part of our efforts in this direction, we are now requesting that all authors identified as 'corresponding author' on published papers create and link their Open Researcher and Contributor Identifier (ORCID) with their account on the Manuscript Tracking System (MTS), prior to acceptance. This applies to primary research papers only. ORCID helps the scientific community achieve unambiguous attribution of all scholarly contributions. You can create and link your ORCID from the home page of the MTS by clicking on 'Modify my Springer Nature account'. For more information please visit please visit www.springernature.com/orcid.

Please do not hesitate to contact me if you have any questions or would like to discuss these revisions further.

****

Reviewers' Comments:

Reviewer #1 (Comments for the Author):

This manuscript presents a comprehensive follow-up study on the role of the Arabidopsis class VIII myosin ATM2 in linking actin cytoskeleton dynamics to plasma membrane nano-organisation. Building on the group's previous work (Fratini et al.; Kastner et al.), the authors now explore how ATM2 modulates phosphatidylinositol 4,5-bisphosphate nano-domain formation and, in turn, influences actin dynamics and ROP signalling at the pollen tube tip.
The study is well-structured, and the experimental approaches are generally sound and carefully executed. The manuscript is clearly written, the data are of high quality, and the authors avoid overinterpreting their results. The work convincingly demonstrates that ATM2 binds both actin and PtdIns(4,5)P_2, that this interaction is required for its plasma membrane localisation and actin-stabilising activity, and that ATM2 can enhance PtdIns(4,5)P_2 abundance and PIP5K2 catalytic activity, thereby contributing to a positive feedback loop promoting local actin stabilisation. Overall, this work provides valuable insights into the molecular mechanisms linking the actin cytoskeleton with membrane lipid organisation and reinforces the central role of PtdIns(4,5)P_2 nano-domains in polarised tip growth. However, while the study is solid and the data primarily support the conclusions, I have several points that should be addressed :

1. While the experiments are carefully performed and the conclusions well supported, the overall conceptual advance beyond the group's previous work (Fratini et al., Kastner et al.) is somewhat limited. Much of the study confirms earlier findings, with the main new elements being (i) the cellular characterisation of ATM2's effect on a PtdIns(4,5)P_2 biosensor and (ii) the interaction between ATM2 and RhoGDI2, an effector of the ROP pathway. These are significant additions but do not fully resolve the physiological significance of ATM2 function, particularly regarding the ROP signalling pathway. The study's impact would be significantly enhanced if the authors complement the transient overexpression experiments with genetic approaches. For example, CRISPR–Cas–generated knockout lines of ATM2 and/or RhoGDI2 would allow testing whether these proteins are indeed required for PtdIns(4,5)P_2 patterning, actin organisation, and ROP signalling in vivo.

Reply:
We thank the reviewer for this comment, and would respectfully point out that we consider the observed effect of ATM2 on actin dynamics to also be quite novel by itself. The notion that effects of ATM2 on actin dynamics require lipid binding and display an apparent specificity for PtdIns(4,5)P2, even though *in vitro* other lipids all bind with similar affinities, provides highly novel insights into how membrane lipids might interact with PM-associating proteins, here in the context of a highly relevant process, actin-PM-attachment.

Given the importance of the observations, we fully agree that a loss-of-function approach deepens our understanding. In the timeframe provided for the revision, it was not possible to obtain CRISPR knock outs as suggested. To address the issue, we have characterized pollen tube growth of an Arabidopsis loss-of-function mutant that is reduced in expression of both pollen-specific class VIII-myosins, ATM2 and VIII-B (Liu et al., Plant Commun 7, 101616, 2026, doi 10.1016/j.xplc.2025.101616). The mutant pollen tubes displayed interesting germination behavior and a growth defect that are consistent with destabilized actin, as is consistent with our ATM2 overexpression data (where actin was over-stabilized). The additional data (Extended Data Figure 4), provide complementary evidence supporting our story.

2. A significant concern is using GFP–mTalin as the primary reporter for actin dynamics. This probe is known to interfere with actin organisation and dynamics, as reported in Ketelaar et al. (Plant Physiol. 2004). The authors should confirm their main findings using alternative actin reporters that are less perturbative, such as Lifeact or an already described actin-binding domain (ABD). These tools would provide more confidence in the observed effects of ATM2 (and truncated versions) on actin dynamics.
Reply:

We thank the reviewer for this suggestion and agree that more than one actin marker provides a better view of potential marker effects on actin dynamics. (Please also see the reply to the equivalent editor's comment above.) We have repeated all actin dynamics measurement with the various truncations of ATM2 by using the less-perturbative marker, LifeAct, and we obtained similar effects on actin dynamics as when we had used the mTalin marker before: the expression of ATM2 stabilizes actin, and lipid binding is required for this effect. While the overall patterns were thus similar between both markers, with LifeAct mostly PM-proximal filaments at the cell periphery were stabilized, whereas the use of mTalin showed some stabilized actin also in the cell center. These differences are likely a consequence of mTalin being a more "perturbative" actin-binding marker, as indicated by the reviewers. We are now citing Ketelaar et al. (Plant Physiol. 136, 3990-3998,2004, doi

10.1104/pp.104.050799) for the effect. Overall, we find our data stronger, as the effects of ATM2 can now be reported based on two independent actin markers. The additional LifeAct data are included in the Extended Data Figure 3.

3. In an elegant set of experiments, the authors tested whether ATM2 binding to PtdIns(4,5)P_2 is consistent with the effects of PIP5K2 (Fratini et al., 2021) or PTase11 on ATM2 function in controlling actin dynamics (cf. Fig. 3). They further explored whether a ~3-fold difference in binding affinities to PtdIns(4,5)P_2 versus PtdIns4P could provide a molecular basis for the PtdIns(4,5)P_2-dependency of ATM2's regulatory role on actin. The data from both the in vivo observation and in vitro assays are consistent with a promoting effect of ATM2 on PtdIns(4,5)P_2 formation in the plasma membrane.
However, the authors did not investigate the contribution of PI4P, although tools such as SAC1 overexpression are available and could be readily employed. Such experiments would help determine whether the observed effects on the PtdIns(4,5)P_2 biosensor are specific or if other phosphoinositides are also involved. This is particularly relevant in light of previous findings showing that ROP signaling is also regulated by the distribution of phosphatidylserine (PS) at the plasma membrane (Platre et al., Science, 2019). Addressing lipid specificity experimentally — or at least discussing this limitation more thoroughly — would significantly strengthen the mechanistic conclusions of the manuscript.

Reply:
We appreciate this comment, and several additional experiments were performed to address the issue, including substantially expanded lipid binding analysis (new Figure 3). The additional MST-analyses show that *in vitro* the C-terminal lipid binding domain of ATM2 did not show a relevant preference for binding to any individual anionic lipid, and it could bind to PtdIns3P, PtdIns4P, PtdIns(3,5)P2, PtdIns(4,5)P2, PtdOH or PtdSer, all with similar affinities. By contrast, the non-anionic structural phospholipid, PtdCho, used as a control (and as a carrier lipid for the liposomes) was bound with substantially lower affinity. As the ATM2 lipid binding region evidently does not only bind PtdIns(4,5)P2 or PtdIns4P, it is difficult to assess whether its retained PM-association upon depleting PtdIns(4,5)P2 is really due to binding to PtdIns4P, or even to the presence of any individual lipid. In fact, of all lipids tested, ATM2 appears to have the weakest affinity for PtdIns4P (see new FIg. 3). We have nonetheless attempted the proposed experiments using the PM-targeted SAC1/SAC1dead modules (Simon et al., Nat Plants 2, 16089 (2026) doi 10.1038/nplants.2016.89) to reduce the PM levels of PtdIns4P, and in consequence also of PtdIns(4,5)P2, which is formed from PtdIns4P. Unfortunately, these experiments did not yield interpretable results, as the expression of SAC1 was not tolerated by the cells, no SAC1-expressing pollen tubes could be recovered, and the cells died. While this observation is not conclusive evidence, we take it as an additional indication for the importance of PtdIns4P/PtdIns(4,5)P2 for pollen tube growth. We regret that we were not able to provide data on the requested experiments.

4. The manuscript refers to overexpression of PTPase11 or SAC9 as "artificial" perturbations of PtdIns(4,5)P_2, while transient expression of PIP5K2 is presented as a means to assess physiological PtdIns(4,5)P_2 function. This distinction is unclear and inconsistent, as overexpression of PIP5K2 is a strong perturbation of PtdIns(4,5)P_2 homeostasis.

Reply:
We agree with the reviewer that any overproduction is artificial. What we intended to say is that SAC9 was used as a tool to manipulate and deplete PtdIns(4,5)P2 at the PM. The text and sub-header have been rephrased accordingly.

Reviewer #2 (Comments for the Author):

In this manuscript, the authors studied how the Arabidopsis class VIII-myosin ATM2 regulates the accumulation/repartition of PtdIns(4,5)P2 and actin dynamics in pollen tubes, which has an impact on the morphology of these later. The authors performed an interesting multidisciplinary approach combining pollen tube swelling assay, cell biology, biochemistry, protein-protein interaction tests, among others. They showed that the overexpression of ATM2 stabilized actin and induces tip swelling in pollen tube cells. ATM2-effects on actin and pollen tube swelling required PtdIns(4,5)P2, although in absence of PtdIns(4,5)P2, ATM2 is properly localized to the plasma membrane. ATM2 overexpression stimulated PtdIns(4,5)P2 abundance in the pollen tube as revealed using a PtdIns(4,5)P2-biosensor. Interestingly, ATM2 interacted with the PI4P 5-kinase, PIP5K2 and could stimulate its activity by an unknown mechanism. The author proposed that ATM2 locally promoted the accumulation of PtdIns(4,5)P2, which self-reinforces ATM2-recruitment and control actin-dynamics and pollen tube morphology.
The work presented in the manuscript is original and interesting and the manuscript is well written. Phenotypical analysis achieved through pollen tube swelling assay as well as biochemistry experiments are convincing. However, I have several caution concerning some cell biology approaches and their interpretation as detailed below. In addition, I believe that some protein-protein interactions need to be validated in plant cells and not only in a heterologous system, yeast. An important question that is note discussed is how the depletion of PtdIns(4,5)P2 interferes with ATM2 function, if it does not disturb its plasma membrane localization. I think that saying that ATM2 modulate membrane lipid nano-organization is not supported by experimental data. Indeed, as detailed below, although the authors show that ATM2 influence PtdIns(4,5)P2 repartition in pollen tubes, the nanoscale organization of the lipid is not affected. In summary, I think that the authors described an interesting process but that needs to be strengthened by some additional experiments and controls as well as by adding additional explanations, as proposed below.

Major comments

Line 29: The title "The class VIII myosin ATM2 stabilizes actin-plasma membrane contacts by locally promoting PtdIns(4,5)P2 nanodomain formation in tobacco pollen tubes" is not appropriated. Indeed, although the authors show that ATM2 overexpression expand the plasma membrane region where the fluorescent PtdIns(4,5)P2-biosensor is detected in pollen tube (Figure 5B), they also show that ATM2 does not modify the nanodomain density of the PtdIns(4,5)P2-biosensor. Therefore, the notion of nanodomain should be retired from the title. The same comment can be done in Figure 7, where the authors indicated in the scheme "locally increased PtdIns(4,5)P2 formation (nanodomain)". This work does not provide evidence that the local concentration of PtdIns(4,5)P2 in nanodomains increases in presence of ATM2.

Reply:

We agree with the reviewer that we are not providing data on the nature of individual PtdIns(4,5)P2 nanodomains. We have therefore changed the title and the instance in the model figure, as requested. At the same time, we have also performed additional analyses on the effects of ATM2 on the nanodomain-distribution of the fluorescent biosensor for PtdIns(4,5)P2, and we are now additionally reporting that the expanded polar PM region enriched in punctate PtdIns(4,5)P2 biosensor signals contains a significantly greater number of nanodomains decorated by the biosensor upon overexpression of ATM2 than was observed upon expression of mC alone, or of a lipid-binding deficient variant of ATM2.

Line 42, summary. The authors say: "ATM2 colocalized and interacted with the PI4P 5-kinase, PIP5K2…"
I did not see any co-localisation experiment between ATM2 and PIP5K2 in the manuscript. The co-localization experiment provided is between ATM2 and the sensor RedStar-PHPLC (Figure 5A).

Reply:
We agree with the reviewer that we had not shown colocalization and that our statement had been misleading. We have now performed additional experiments to address the spatial relations of coexpressed ATM2 and PIP5K2. Coexpression of PIP5K2 with full-length ATM2 might be lethal. Therefore, we analyzed the coexpression of PIP5K2 with the slightly shorter ATM2deltaNT variant, and we observed very similar PM patterns of ATM2deltaNT and PIP5K2 by spinning disc microscopy, presented in Figure 1 with the notice that the shorter ATM2 protein variant was used.

Figure 1A: Please specify which promoter was used to express EYFP-ATM2. If a constitutive promoter was used, why is pollen tube tip swelling not observed as in Figure 2B where ATM2 was over-expressed with pLat52 promoter? This needs some precisions.

Reply:
We apologize for the oversight. All experiments were performed using the pollen-specific Lat52-promoter, and this is now stated in the legends to all relevant figures. Please note that changes in cell morphologies will not be apparent immediately when transgene expression starts and fluorescence commences. For the analysis of subcellular distributions of fluorescence markers, we always aim to analyze cells that are not obviously altered in morphology. The pollen tube shown in Figure 2B is observed early, at only 4 h after transformation, and the cell is growing normally. By contrast, to analyze the effects on cell morphology, pollen tubes are analyzed at later stages of growth, and/or upon higher levels of transgene expression (as evident from higher fluorescence intensities). Please note that for the transient expression experiments the degree of expression can be modulated by using more or less plasmid DNA, even when using constructs with the same promoters. Experiments shown in Figure 2B have been performed with low concentrations of plasmid DNA. Information on the amounts of plasmid DNA used has been included in the methods section.

Figure 1B: PIP5K2-EYFP is supposed to be localized in the plasma membrane but, as especially observed in the detail view (white box), a PIP5K2-EYFP signal is also detected under the plasma membrane. The authors should comment this localization.

Reply:
We thank the reviewer for this insightful observation. Images in Figure 1B can not easily be interpreted with regard to the PM distribution of PIP5K2-EYFP, as the images do not represent individual confocal sections but are substacks over several confocal frames. The substacks were chosen to enable tracing of the three-dimensional network of filaments decorated by the coexpressed actin marker to the points where the filaments intersect with the PIP5K2-EYFP-signals. While, a crisp PM signal cannot be derived from such substacks, here we aimed to see whether the ends of filaments would converge onto sites occupied by PIP5K2-EYFP. This analysis is now included in Figure 1. While previous work has shown PIP5K2-EYFP at the PM (e.g., Fratini et al., Plant Cell 33, 642-670, 2021, doi 10.1093/plcell/koaa035), we have previously also observed that PIP5K2-EYFP can occupy an intermittant state immediately inside of the PM, likely associating with PM-proximal actin filaments (Fratini et al., Plant Cell 33, 642-670, 2021, doi 10.1093/plcell/koaa035). In line with this notion, an N-terminally truncated variant of PIP5K2 lacking the domain mediating PM association decorated PM-proximal filamentous structures (Extended Data Figure 1), and we conclude that the PIP5K2 protein has an intrinsic capacity to associate with the peripheral cytoskeleton. We have expanded our discussion of these observations in the revised text.

Figure 1B: Qualitative data are provided, but please provide also quantitative information by measuring the proportion of actin filaments associated with PIP5K2-EYFP domains.

Reply:
We appreciate this suggestion and have performed the requested quantitative analysis, as noted in our response to the previous comment. In brief, we analyzed the incidence of actin marker signals as a function of the distance from the PIP5K2-EYFP-signals. The analysis shows that the abundance of actin marker signals is increased in close proximity to PIP5K2-EYFP signals. The data are part of the revised Figure 1.

Figure 1C:
In this figure, I do not understand how the authors can state that when mC-ATM2 was co-expressed with mTalin-YFP marker for actin, they observed enhanced actin signals at the membrane-proximal ends of actin filaments intersecting with the plasma membrane. How was it quantified? Did they compare with the situation where mTalin-YFP was expressed alone, without mC-ATM2? To facilitate the interpretation of the data, please provide all the channels and not only the merge pictures.

Reply:
We appreciate this comment and have altered the figure to now represent the individual channels as well as the merged overlays. In the separate mTalin-YFP channel, an enhanced signal intensitiy at the intersection with the PM can now clearly seen. The intensitiy of the actin marker fluorescence was now quantified along the filaments and showed a marked increase when the filaments intersected with the mC-ATM2-decorated PM (also quantified). The pattern was observed upon expression of Arabidopsis ATM2 as well as for the tobacco homolog, NtMyo2-like. The data are shown as part of the revised Figure 2. While the data clearly show enhanced actin marker fluorescence at the PM, this pattern

can not easily be compared to a control expressing the actin marker with mC only, as with no ATM2 present, the filaments in the cell apex cannot be seen or imaged. Therefore, we regret that this requested control cannot be provided.

Figure 2B: The authors propose a locally increased mTalin-YFP signal at the plasma membrane in presence of ATM2. How do the authors reconciliate the homogenous distribution of ATM2 in the plasma membrane with a patterning of actin in hot spots in the plasma membrane?

Reply:
We thank the reviewer for this comment. We think that this apparent incongruence is a consequence of using different methods of imaging. When imaged by LSM, a homogonous distribution of ATM2 was observed. By contrast, when ATM2 was imaged by SD focused on the peripheral PM plane, we always observed a punctate or reticulate pattern of ATM2 at the PM. We have added better labels throughout our figures to indicate by what technique images were acquired (LSM or SD). The particular images in Figure 2B have all been recorded by LSM and do not show the ATM2 marker in domains. Despite using the LSM, we could consistently observe the increased actin marker fluorescence at the PM, possibly because it is very bright, and/or because there are fewer "active" actin attachment points than there are ATM2 signals at the PM. In any case, the appearance of the bright PM-proximal actin signals prompted us to further investigate the effects of ATM2 on actin dynamics, and further data, including the analyses of actin occupancy and spatial persistence according to kymographs, provide ample evidence supporting this notion. The data on actin dynamics have been additionally supported by the use of the alternative actin marker, LifeAct, and the data are presented as part of Figure 2 (mTalin) and Expanded Data Figure 4 (LifeAct).

Figure 3A: The fact that PtdIns(4,5)P2 depletion using PTase11 absolutely does not disturb ATM2 plasma membrane localization is intriguing, especially because the authors then show that ATM2 preferentially binds to PtdIns(4,5)P2 (Figure 4). In the discussion the authors propose that PtdIns4P could ensure the anchoring of ATM2 in the plasma membrane. I think it needs some precisions.

Reply:
We thank the reviewer for this comment and agree that the observation of retained PM-association of ATM2 upon depletion of PtdIns(4,5)P2 is a key observation of our study, in particular, as the effect of still-PM-associated ATM2 on actin dynamics was abolished when PtdIns(4,5)P2 was depleted. Retained PM association of ATM2 upon PtdIns(4,5)P2-depletion is consistent with the expanded lipid-binding data showing that ATM2 can bind various anionic lipids *in vitro*, with no preference for any particular lipid (Figure 3). Based on the expanded lipid-binding analyses, and as several of the bound lipids reside in the PM, it remains unclear which lipid is responsible for the retained PM-association of ATM2 upon PtdIns(4,5)P2-depletion. Please note that based on the newly acquired lipid-binding data we no longer consider the mild preference of the lipid-binding domain of ATM2 for PtdIns(4,5)P2 over PtdIns4P as relevant to explain the effect of ATM2 on actin. Our discussion has been adjusted to include this notion, and our model proposes that localized amplification of PtdIns(4,5)P2 production by activation of PIP5K2 might undelie the apparent PtdIns(4,5)P2-specific effect on actin dynamics. Where PtdIns(4,5)P2 is substantially overproduced, this lipid might be responsible for ATM2 recruitment to the PM and actin stabilization, even though ATM2 has no preference for binding PtdIns(4,5)P2 over other lipids. Please also note that the proposed (but failed) SAC1/SAC1dead experiment (aiming to deplete PtdIns4P from the PM) might not have helped interpreting the mode of retained PM association of ATM2, as other lipids also bind, such as PtdSer or PtdOH.

Figure 3: Please provide a control showing that PtdIns(4,5)P2 depletion indeed occurs in this system by expressing PTase11. It can be easily monitored using the PtdIns(4,5)P_2 biosensor, RedStar-PHPLC.

Reply:
We thank the reviewer for this suggestion and have performed the requested experiment. The expression of PTase11 significantly decreased the ratio of PM/cytosolic fluorescence of the RedStar-PLC-PH marker as well as the spatial extend of the subapical PM region that is occupied by the RedStar-PLC-PH marker. Both results are included as the new Extended Data Figure 5.

Figure 3A. Compared to figures 1 and 2, some images seem to be post-processed such as CFP-ATM2 (top and bottom) and mCherry (top). Please provide other images.

Reply:
We thank the reviewer for this observation and have re-evaluated our images. None of the images are post-processed. The graininess of the images (new Figure 4) is due to the use of low fluorescence intensities of the markers tested, so as to not perturb pollen tube growth. Please note that all of the constructs used (including those for the PTase11 or SAC9 shown here), obstruct pollen tube growth and cannot be expressed at high intensities, or there would be no growing pollen tubes to analyze. We consider this an intrinsic limitation of the experimental setup, and we regret that we cannot provide better images at this stage without compromising pollen tube growth and/or cell morphology.

Figure 3. While mC-Control is indicated in the graphs (C), the corresponding images are not indicated in (A), please provide them.

Reply:
We thank the reviewer for this suggestion and apologize for the omission. We have now included the respective images as well as additional analyses in the (new) Figure 4.

Figure 4A. Please provide SDS-PAGE + coomassie blue staining of the purified proteins: MBP-His6, ATM2CT-His6 and ATM2CTΔLB-His6.

Reply:
We thank the reviewer for this suggestion and have included the requested information in (new) Figure 3. Beyond the requested instances, we are now providing Coomassie-stained SDS-PAGE gels for all recombinant proteins used in any experiment, e.g., also in (new) Figure 6.

Figure 4. Even if I fully understand when authors say that "a drawback of the lipid overlay assay is that lipids are presented in very high abundance but not in a membraneous formulation, and it may be difficult to assess quantitative differences in lipid", I am a little bite surprise

that the two lipids displaying the highest affinity/signal for ATM2CT-His6 (PtdIns3P and PtdIns5P) (Figure 4A) were not analyzed by microscale thermophoresis. Therefore, I think it is a little bite overstated to say line 262 that "ATM2 displays a binding preference for PtdIns(4,5)P2 in vitro" since the only comparison is done with PtdIns4P. At least, please modify the sentence.

Reply:
We thank the reviewer for this comment and agree that this statement needed to be adjusted. The expanded lipid-binding studies (new Figure 3) clearly show that the C-terminal ATM2 lipid-binding region can bind to PtdIns-monophosphates, PtdIns-bisphosphates, PtdOH or PtdSer, with close-to-equal affinity *in vitro*. We have included these data, and in the revised text we have expanded our explanations that preferential lipid binding to PtdIns(4,5)P2 cannot be the reason for the apparent PtdIns(4,5)P2-specific effect of ATM2 on actin dynamics. Please note that PtdIns5P has not been analyzed, as this lipid is likely not at the PM and is currently not well understood.

Figure 5A. In this co-localization analysis, controls are missing. As a negative control, please provide data with a lipid sensor or a plasma membrane protein that does not colocalize with ATM2 in nanodomains. A positive control for colocalization in nanodomains, for instance by co-expressing EYFP-ATM2 and mCherry-ATM2, would be also important.

Reply:
We appreciate this comment and have performed the requested analyses. In brief, the positive control for colocalization (mC-ATM2 vs. EYFP-ATM2) resulted in close to ideal co-localization, and the respective Pearson's coefficient was higher that than observed for ATM2 vs. RedStar-PLC-PH. By contrast, a significantly lower Pearson's coefficient was observed when EYFP-ATM2 was compared to the diffuse PM marker, mC-LTI6b (Stellmach et al., Plants 11, 2620 (2022) doi 10.3390/PLANTS11192620/S1). The analyses shown in the new Figure 5 provide evidence that the partial colocalization of EYFP-ATM2 with the PtdIns(4,5)P2-biosensor is not coincidental.

In figure 5A, EYFP-ATM2 protein seems to organize, at least partially, in plasma membrane nanodomains, whereas in figure 1A, the same protein displays a homogenous distribution in the plasma membrane. Since in both analyses spinning disc microscopy was used and z-projections are presented, please comment this inconsistency.

Reply:
We appreciate the comment but are a bit confused, as for our eyes the images in Figure 1 A show a quite inhomogenous PM pattern for the EYFP-ATM2 marker. This inhomogenous pattern was always observed when fluorescent fusions of ATM2 were imaged by spinning disc microscopy. We are now providing an added detail view of the EYFP-ATM2deltaNT marker imaged by spinning disc microscopy in Figure 1 E to illustrate the PM pattern in relation to that of PIP5K2-mC.

Figure 5B. The nanodomain density of RedStar-PHPLC is presented but without the corresponding microscopy images. Please add images showing RedStar-PHPLC nanodomains without ATM2, with ATM2 and with ATM2deltaLB.

Reply:
We thank the reviewer for this comment and agree that these images should be shown. We have included the requested images as part of the revised new Figure 5.

Figure 5C: Are the authors sure that myosins XI are correctly produced in yeast since no interaction was detected in this test and no positive control of the interactions between a given myosin XI and another protein is provided?

Reply:
We thank the reviewer for this comment and agree that the negative outcome of the tests against class XI-myosins require a positive control. We apologize for the omission. To address this concern, we used our yeast-two-hybrid setup to test for the known interactions of the class XI-myosin, XI-2, against RabC2a and RabD1 (Hashimoto et al., JXB 59, 3523-3531, 2008; doi 10.1093/jxb/ern202). Both controls tested positive in our hands, confirming that myosin XI-2 was expressed. We did not perform further controls for all the other class XI-myosins. Beyond this requested experiment, we performed a positive control also for the test for interaction of ATM2 against PIP5K6, which had also tested negative and required a positive control. As a positive control, we used the known interaction of PIP5K6 with MPK6 (Hempel et al., Plant Cell 29, 3030-3050, 2017; doi 10.1105/tpc.17.00543), which also tested positive, confirming that PIP5K6 was expressed. The additional controls aid the interpretation of our data and have been included in the respective Figures 1 G and Extended Data Figure 1 A.

In figure 5, the interaction between ATM2 and PIP5K2 proteins was only investigated in a heterologous system, yeast. The same remark can be done concerning the interaction tests presented in Figure 6A. Focusing on ATM2/PIP5K2, NtRhoGDI2/PIP5K2 and NtRhoGDI2/ATM2 couples, the authors should provide evidences for protein-protein interactions in plant cells by the technic of their choice (co-IP, FRET…).

Reply:
The *in planta* interaction at the center of our study is that of ATM2 with PIP5K2, and we performed FRET/FLIM experiments to verify the interaction in vivo in pollen tube cells, as suggested. We have been able to record FRET/FLIM data for ATM2deltaNT vs. PIP5K2 and observed a significant reduction of the CFP-ATM2deltaNT donor fluorescence lifetime in the presence of PIP5K2-EYFP, but not for the EYFP control. The interaction data are presented in Figure 1 noting that the shorter ATM2 variant was used.

Figure 5D: It is difficult for the reader to easily interpret this panel. Please indicate in the legend what the black and white arrowheads correspond to. What does the intermediate band without any arrowhead labelling correspond to?

Reply:
We apologize if the labeling of our panel was not complete, and we have expanded the explanation of this non-standard experiment for the new Figure 6. The data presented in this panel are the results of *in vitro* lipid phosphorylation tests, where purified recombinant PIP5K2 protein is

tested for the formation of radiolabeled PtdIns(4,5)P2. Radiolabeled lipids extracted from the assays were separated by thin-layer-chromatography (TLC) and detected by phosphorimaging of the radiolabeled lipid products. We apologize for omitting the description of the arrowheads: The black arrowhead indicates the migration of the reaction product, PtdIns(4,5)P2, in the TLC and the white arrowhead points at the origin of migration, where the samples were applied to the TLC. Both labels are now included in the revised Figure 6, along with a third grey arrowhead indicating the migration of a non-specific lipid. PIP5K2 was recombinantly expressed in *E. coli*, and *E. coli*-extracts from cells not expressing PIP5K2 were used as a negative control. As the additional lipid band in question was also present in these negative controls, this band is not related to the activity of PIP5K2. The band might represent a derivative of ATP or another unidentified radiolabeled lipid formed in the *E. coli* extract. To aid the understanding of this complex experiment, we have now also provided Coomassie-stained SDS-PAGE gels of all proteins used in the analysis in the new Figure 6.

Figure 6B and C: The authors claim that the expression of NtRhoGDI2-EYFP influenced the plasma membrane distribution of mC-ATM2 along the pollen tube. However, I think that the microscopy images in 6B do not support that. Indeed, I cannot observe that mC-ATM2 distribution is more restricted to the tip when co-expressed with NtRhoGDI2-EYFP. What is seen is that mC-ATM2 signal is maybe more intense at the tip in presence of NtRhoGDI2-EYFP, but not more confined. In addition, according to the spatial distribution of mC-ATM2 signal in panel C, a peak of fluorescence should be observed at around 60 um from the tip in presence of unfused EYFP. This peak cannot be observed in the image provided in panel B. The authors need to clarify these two points.

Reply:
We appreciate this comment and share the reviewer's concern. We find that the GDI-related aspects of our study might not have been sufficiently explored and will require more work to become conclusive. As for our revision we are facing the task of providing a substantial volume of additional data and analyses while at the same time aiming to shorten the manuscript, we have chosen to remove the GDI-related aspects from the manuscript, alltogether. With the focus on the interplay of ATM2 and PIP5K2, GDI is now only mentioned briefly with regard to the positive yeast-two-hybrid analysis (Extended Data Fig. 1 B), which supports parts of our model (Fig. 7). We hope that the reviewer can follow our reasoning why we have not performed further experiments or made any more explanations on the GDI.

Concerning the data from Figure 6, it remains unclear how NtRhoGDI2-EYFP abolishes the tip swelling induced by ATM2. Please provide hypotheses.

Reply:
Our data on the interplay between ATM2 and PIP5K2 identifies ATM2 as a modulator of PtdIns(4,5)P2-formation, with effects on actin dynamics. The model proposed by Kost et al., Trends Cell Biol 18, 119-127, 2008, doi 10.1016/j.tcb.2008.01.003) and revisited by Ischebeck et al., Plant J 65, 453-468, 2011, doi 10.1111/j.1365-313X.2010.04435.x and Fratini et al., Plant Cell 33, 642-670, 2021, doi 10.1093/plcell/koaa035) proposes that PtdIns(4,5)P2 acts as a GDF, inhibiting GDI function, resulting in the release of ROPs for activation at the PM. The observation of GDI as a functional antagonist of ATM2 is fully consistent with this model. However, as we have chosen to remove the GDI-related aspects from the manuscript (see previous point), this discussion is no longer a focus of our manuscript.

In several sentences of the manuscript, the authors say that ATM2 stabilizes actin-dynamics. To my opinion it sounds weird because “dynamics” and “stabilization” are antinomic.

Reply:
We thank the reviewer for this comment and agree that the previous phrasing was ... clumsy. We have rephrased all instances in the text to read "reduces actin dynamics".

Minor comments

The title of Figure 1 is “The PtdIns(4,5)P2 biosensor RedStar-PHPLC and EYFP-ATM2 occupy overlapping subapical plasma membrane domains of tobacco pollen tubes, coinciding with actin-plasma membrane contacts”. I suggest to replace “domains” by “zones” to avoid confusion with nanodomains.

Reply:
We appreciate the comment and have changed the title of Figure 1 accordingly.

Figure 4 C and D. To facilitate the comparison between the lipid binding properties of sfGFP-ATM2CT and sfGFP-tag alone, it would be preferable to present all the data on the same graph.

Reply:
We thank the reviewer for this suggestion, but respectfully prefer to retain the figures as having separate panels. First, the additional lipid analyses result in figures with many curves already, where the binding of the individual lipids can be compared. We think it is not advised to have even more curves in the same panel. Moreover, from the technical point of view, lipid binding is most intuitively reported as "fraction bound", indicating what proportion of a fluorescence-labeled protein is binding to increasing concentrations of liposomes provided. However, the "fraction bound" ratio cannot be calculated if there is no apparent binding, as for the sfGFP-negative controls. Therefore, the MST data for these controls are given as the more technical "deltaFnorm"-value, which describes their behavior in the MST analysis, even without any binding. We hope that this explanation will be found convincing, as to why we retain the original setup of the figure.

I think it could be interesting to cite in the manuscript the work of Wang et al. 2024, PNAS, on myosin XI nanodomain organization.

Reply:
We thank the reviewer for this suggestion and have included mention of this interesting reference in our discussion.

Itemized response to reviews

All comments were retained, our replies are highlighted.

NPLANTS-250920873A

4th August 2026

Dear Dr. Heilmann,

Thank you for submitting your revised manuscript "The class VIII myosin ATM2 stabilizes actin-plasma membrane contacts in tobacco pollen tubes by locally promoting PtdIns(4,5)P2 formation" (NPLANTS-250920873A). It has now been seen by the original referees and their comments are below. The reviewers find that the paper has improved in revision, and therefore we'll be happy in principle to publish it in Nature Plants, pending minor revisions to satisfy the referees' final requests and to comply with our editorial and formatting guidelines.

We are now performing detailed checks on your paper and will send you a checklist detailing our editorial and formatting requirements in a couple of weeks. Please do not upload the final materials and make any revisions until you receive this additional information from us.

Thank you again for your interest in Nature Plants Please do not hesitate to contact me if you have any questions.

Sincerely,

Raphael Trösch
Editor
Nature Plants

Reviewer #1 (Comments for the Author):

The reviewer previously questioned the specificity of ATM2 binding to PtdIns(4,5)P_2. The authors' new data show that ATM2 binds anionic lipids non-specifically, remains associated with the plasma membrane after PtdIns(4,5)P_2 depletion, yet loses its actin regulatory function. The reviewer advises removing claims of PtdIns(4,5)P_2 specificity from the title, abstract, and figure legends. For example, the abstract sentence "ATM2 binds both actin and phosphatidylinositol 4,5-bisphosphate (PtdIns(4,5)P_2), a lipid required for ATM2-PM attachment" could be revised to: "ATM2 binds both actin and anionic lipids, including PtdIns(4,5)P_2, which is required for ATM2-PM attachment."

Reply
We agree with the reviewer and have removed unsubstantiated claims from the abstract, the figure legends and throughout the main text. Please note that the title did not contain an unsubstantiated claim and is, thus, retained.

The authors should consider adding a section on "limitations of the study" in the Discussion, as not all the requested experiments were done, mainly because of time and technical problems.

Reply
We thank the reviewer for this suggestion and have added a brief section on limitations of the study in the discussion, as requested.

I could not find information about the lines/constructs used in this study. A recap table in the Materials and Methods would be useful. For example, the constructs mTalin:YFP and YFP-mTalin (Supplemental Figure 3 legend and pannels) are mentioned, but there is no information about the differences between them.

Reply
We thank the reviewer for this suggestion, and we have included a table with the constructs in the Methods section, as suggested. The particular instance (mTalin:YFP vs. YFP-mTalin) represents a mislabel on our part, as there is only mTalin-YFP throughout. We apologize for the mistake, which has been corrected.

In general, I would prefer graphs to display individual data points rather than only mean values. It would also be more reader-friendly to consistently include n= (sample size) and N= (number of experiments) on all graphs. This information is sometimes in the legend but not always.

Reply
We thank the reviewer for this suggestion and have altered all data figures to now include the individual data points as well as the values for N and n.

Specific Comments
Line 942: Spelling mistake – "ceels" → "cells". Please indicate the number of replicates.

Reply
The spelling error was corrected. The number of replicates is now stated.

Figure 1F: Spelling mistake – "ceels" → "cells". Please indicate the number of replicates. The authors should justify the sample size (n=4 cells) and provide the raw data in the supplemental material, as the sample size is very small.

Reply
The spelling error was corrected. The number of replicates is now stated. The small sample size is a result from the low incidence of recovered cells that display coexpression of both markers. The raw data have been included in the supplemental material.

Figures 2A and 2D: Scale bars are missing.
Reply
The scale bars have been added. We aplogize for the omission.

Figure 2F: N=?

Reply
The N-value has been added

Figure 4: There is substantial variation in sample sizes for the mC-control (20–29 cells) vs. ATM2 (only 6 cells). Please explain this discrepancy and ensure it does not affect the statistical analysis. Given the small sample size (n=6), I encourage the authors to show the individual data points in the supplemental material.

Reply
The requested images have beend added.

Figure 4E: N=?
Figure 4F: n=? N=?
Figure 5: N=?

Reply
The requested values have beend added.

Figure labels: The authors refer to the RedStar-PH biosensor inconsistently as "red channel" or "mC". This is confusing; please clarify and standardize the labels throughout the manuscript.

Reply
We apoligize for the inconsistencies. We have now changed all legends to be consistent.

Figure 6B: "detail" is unclear. Please add a scale bar and clarify the panel.

Reply
We have added the missing scale bar and description of the detail view in the figure legend.

Figure 6F: Please show the full blot in the supplemental material.

Reply
The proteins were from multiple separate protein expressions and purifications and are shown as Coomassie-stained SDS-PAGE gels. We have added all full SDS-PAGE gels to the supplemental materials as requested.

Figure 6C: N=?
Figure 6E: n=? N=?
Supplemental Figure 1D: n=? N=?
Supplemental Figure 2B: N=?

Reply
The requested values have been added.

Supplemental Figure 4B: spelling mistake "Data are from three independen experiments"

Reply
The typo was corrected.

Supplemental Figure 5B: N=?

Reply
The missing value was added.

Supplemental Figure 5: Only 5 pollen tubes were analyzed for Ptase11-YFP. Given the small sample size, please display the images. N=? n=?

Reply
The images were included and the missing values were added.

Supplemental Figure 6A: Only 6 cells were analyzed for CFP-ATM2. Please display the images, as the sample size is very small.

Reply

The images were added as requested. We also added the respective individual kymographs.

Model: Since the paper no longer focuses on RhoGDI, should the authors retain it in the scheme?

Reply
We have removed the RhoGDI from the model, which simplifies the model and might be more intuitive.

Reviewer #2 (Comments for the Author):

In this revised manuscript, the authors performed numerous additional experiments and carefully answered to all my comments. To my opinion, this work is very interesting and is now complete. Below, I have just few comments on the additional experiments that were provided in this revision. Although additional experiments are not required, I suggest the authors to rearrange some figures, measure PCC values for the colocalization test in Figure 1E, and justify with more details in the manuscript the use of ATM2 variant lacking part of its N-terminus.

Reply
We have added a section on the use of the truncated ATM2 variant in the section "limitations of the study" that was requested by reviewer 1. We hope this will sufficiently address this concern.

Line 42, summary. The authors say: “ATM2 colocalized and interacted with the PI4P 5-kinase, PIP5K2…”
I did not see any co-localisation experiment between ATM2 and PIP5K2 in the manuscript. The co-localization experiment provided is between ATM2 and the sensor RedStar-PHPLC (Figure 5A).

Reply:
We agree with the reviewer that we had not shown colocalization and that our statement had been misleading. We have now performed additional experiments to address the spatial relations of coexpressed ATM2 and PIP5K2. Coexpression of PIP5K2 with full-length ATM2 might be lethal. Therefore, we analyzed the coexpression of PIP5K2 with the slightly shorter ATM2deltaNT variant, and we observed very similar PM patterns of ATM2deltaNT and PIP5K2 by spinning disc microscopy, presented in Figure 1 with the notice that the shorter ATM2 protein variant was used.

Reviewer 2. In this co-localization analysis (Figure 1E), I understand the possible limit of working with the full length ATM2 protein but are the authors sure that ATM2 and ATM2deltaNT display the same subcellular localization? I read in their response that this truncated ATM2 protein was used in previous studies, but I think that this choice needs to be at least briefly explained in the manuscript.

Reply
As we have not been able to perform the colocalization experiment using full-length ATM2, this issue is hard to address. In the end, possible differences between full-length ATM2 protein and the shorter protein variant (and thus a possible function of the short N-terminal part present in full-length) remain currently unclear. We have added a section on the use of the truncated ATM2 variant in the section "limitations of the study" that was requested by reviewer 1. We hope this will sufficiently address this concern.

In Figure 1E, it seems that ATM2 and PIP5K2 are co-localized but I suggest to the authors to quantify this colocalization to strengthen the conclusion, for instance by measuring the Pearson’s correlation coefficient. A negative control for colocalization, for instance ATM2 with an unrelated protein, would have been beneficial, but it is probably not obligatory given the rather particular localization of the

two proteins of interest.

Reply
We have added the Pearson's coefficients, as requested.

Figure 1C:
In this figure, I do not understand how the authors can state that when mC-ATM2 was co-expressed with mTalin-YFP marker for actin, they observed enhanced actin signals at the membrane-proximal ends of actin filaments intersecting with the plasma membrane. How was it quantified? Did they compare with the situation where mTalin-YFP was expressed alone, without mC-ATM2? To facilitate the interpretation of the data, please provide all the channels and not only the merge pictures.

Reply:
We appreciate this comment and have altered the figure to now represent the individual channels as well as the merged overlays. In the separate mTalin-YFP channel, an enhanced signal intensitiy at the intersection with the PM can now clearly seen. The intensitiy of the actin marker fluorescence was now quantified along the filaments and showed a marked increase when the filaments intersected with the mC-ATM2-decorated PM (also quantified). The pattern was observed upon expression of Arabidopsis ATM2 as well as for the tobacco homolog, NtMyo2-like. The data are shown as part of the revised Figure 2. While the data clearly show enhanced actin marker fluorescence at the PM, this pattern can not easily be compared to a control expressing the actin marker with mC only, as with no ATM2 present, the filaments in the cell apex cannot be seen or imaged. Therefore, we regret that this requested control cannot be provided.

Reviewer 2. Without presenting a control where mTalin-YFP was expressed alone, without mC-ATM2, I believe it is difficult to conclude on a putative effect of ATM2 solely using panel A (Figure 2). However, the authors provide in Figure 2 panel D pictures where mTalin-YFP was co-expressed with mC, which is to my opinion the required control for panel A, even if quantification is not possible for this control. Therefore, I suggest to present this control earlier in the manuscript or at least to cite panel D when interpreting panel A.

Reply
We thank the reviewer for this suggestion and agree that the actin distribution pattern in control cells should be mentioned. The reference to the YFP-mTalin/mC control (panel (D)) has been added to the text, as suggested.

In figure 5A, EYFP-ATM2 protein seems to organize, at least partially, in plasma membrane nanodomains, whereas in figure 1A, the same protein displays a homogenous distribution in the plasma membrane. Since in both analyses spinning disc microscopy was used and z-projections are presented, please comment this inconsistency.

Reply:
We appreciate the comment but are a bit confused, as for our eyes the images in Figure 1 A show a quite inhomogenous PM pattern for the EYFP-ATM2 marker. This inhomogenous pattern was always observed when fluorescent fusions of ATM2 were imaged by spinning disc microscopy. We are now providing an added detail view of the EYFP-ATM2deltaNT marker imaged by spinning disc microscopy in Figure 1 E to illustrate the PM pattern in relation to that of PIP5K2-mC.

Reviewer 2. I agree that in Figure 1B (new figure), the localization of EYFP-ATM2 is inhomogenous, as stated by the authors, however it is difficult to see nanoclusters to my opinion, contrary for instance to what is observed with RedStar-PHPLC that clearly arranges in nanodomains. That is why I thought that the picture with EYFP-ATM2 was probably not representative. In the new figure 1E, EYFP-ATM2ΔNT is also not homogeneously distributed, but again, I think it is not localized in clear

nanodomains. I just wanted to make the authors aware of these aspects.

Reply
We thank the reviewer for this comment. To address this issue, we have changed the phrasing to now refer to an "inhomogenous distribution". We hope this addresses this concern.

In figure 5, the interaction between ATM2 and PIP5K2 proteins was only investigated in a heterologous system, yeast. The same remark can be done concerning the interaction tests presented in Figure 6A. Focusing on ATM2/PIP5K2, NtRhoGDI2/PIP5K2 and NtRhoGDI2/ATM2 couples, the authors should provide evidences for protein-protein interactions in plant cells by the technic of their choice (co-IP, FRET…).

Reply:
The in planta interaction at the center of our study is that of ATM2 with PIP5K2, and we performed FRET/FLIM experiments to verify the interaction in vivo in pollen tube cells, as suggested. We have been able to record FRET/FLIM data for ATM2deltaNT vs. PIP5K2 and observed a significant reduction of the PIP5K2-EYFP donor fluorescence lifetime in the presence of mC-ATM2deltaNT, but not for an EYFP control. The interaction data are presented in Figure 1 noting that the shorter ATM2 variant was used.

Reviewer 2. Figure 1F. Thank you for performing this very important experiment. I suggest to the authors to indicate in their graph the fluorescence lifetime of the donor alone (-ATM2ΔNT), which is usually presented in FRET-FLIM analyses. It will allow to compare the fluorescence lifetime in presence of EYFP and PIP5K2-EYFP.

Reply
We apologize for the mistake and that our labeling was not correct. We thank the reviewer for this suggestion and are now presenting the FRET/FLIM data as the lifetime of donor alone vs. the lifetime of the donor in the presence of the acceptor.

I do not understand what is the meaning/interest of the values Mean τ (int.) and Mean τ (amp.). For Mean τ (amp.), there is no statistical difference between the negative control EYFP and PIP5K2-EYFP.

Reply
We agree with the reviewer that the main information is the change in donor fluorescence lifetime. To address this comment, we have removed the redundant/unnecessary information on Mean τ (int.) and Mean τ (amp.), which just represent additional read-outs of the FRET/FLIM analysis.

The results appear conclusive, showing statistical differences between the negative control and the interaction test (CFP-ATM2ΔNT+ PIP5K2-EYFP), however, I would still like to point out that the observed decrease in the tau value for CFP-ATM2ΔNT+ PIP5K2-EYFP compared to the negative control is slight.

Reply
We agree with the reviewer and have added the "slight" in the text.

I would suggest to the authors to add a panel showing the FRET-FLIM acquisition with the fluorescence lifetime indicated by a pseudo color code. Indeed, this information is interesting for the readers since one of the advantages of FRET-FLIM is to “visualize” interaction at the subcellular level. This panel could replace the table with fluorescence lifetime values that is, to my opinion, not very informative compared to the graph that is presented in Figure 1F.

Reply

We agree with the reviewer and have aded a false-color image indicating the spatial distribution of the FRET/FLIM signals. We also agree that the table with the numerical values is redundant with the graph in panel (F) and the table was removed as suggested.

INTERNAL USE ONLY AIP modules required: ["Life Science"]

This email has been sent through the Springer Nature Tracking System NY-610A-NPG&MTS

Confidentiality Statement:

This e-mail is confidential and subject to copyright. Any unauthorised use or disclosure of its contents is prohibited. If you have received this email in error please notify our Manuscript Tracking System Helpdesk team at https://digitalsubmissions.freshdesk.com/support/tickets/new .
Details of the confidentiality and pre-publicity policy may be found here http://www.nature.com/authors/policies/confidentiality.html
Privacy Policy | Update Profile
