# Supplementary material for: The class VIII myosin ATM2 stabilizes actin–plasma membrane contacts in tobacco pollen tubes by locally promoting PtdIns(4,5)P2 formation
Source: Nat Plants. 2026 Oct 16;12(10):2089–107. doi: 10.1038/s41477-026-02427-0 (PMC13680279; doi:10.1038/s41477-026-02427-0)
Supplement: Supplementary file 4 [file 41477_2026_2427_MOESM4_ESM.pdf]

left, related to Fig. 3 b

MBP-His_6 elution fraction used

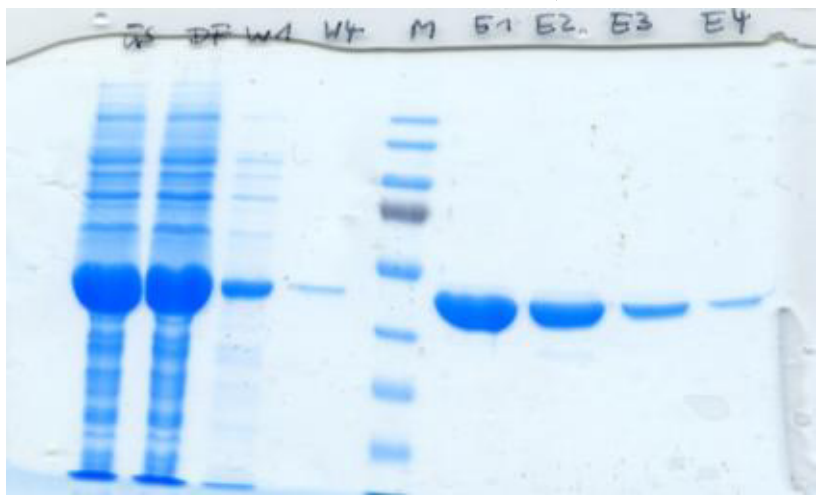

42 kDa

$ATM2_{CT\Delta LB}$-His_6 elution fraction used

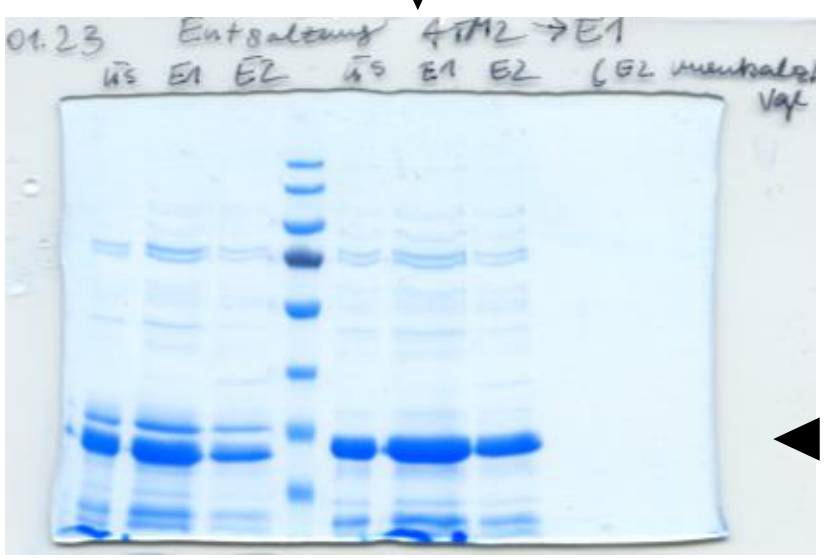

28 kDa

$ATM2_{CT}$-His_6 elution fraction used

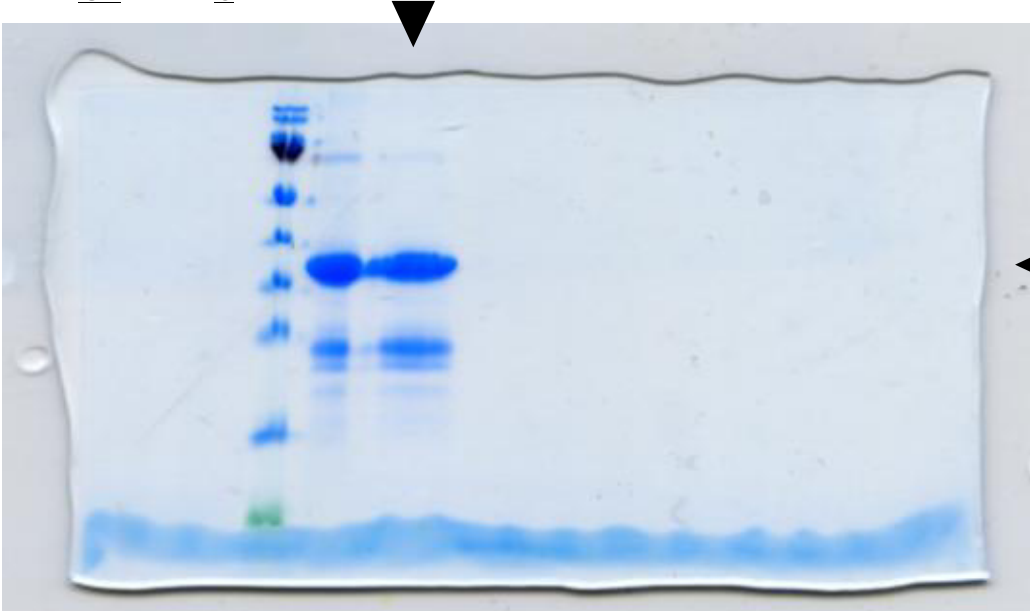

37 kDa

right, related to Fig. 6 f

MBP-PIP5K2 elution fraction used

M

129 kDa

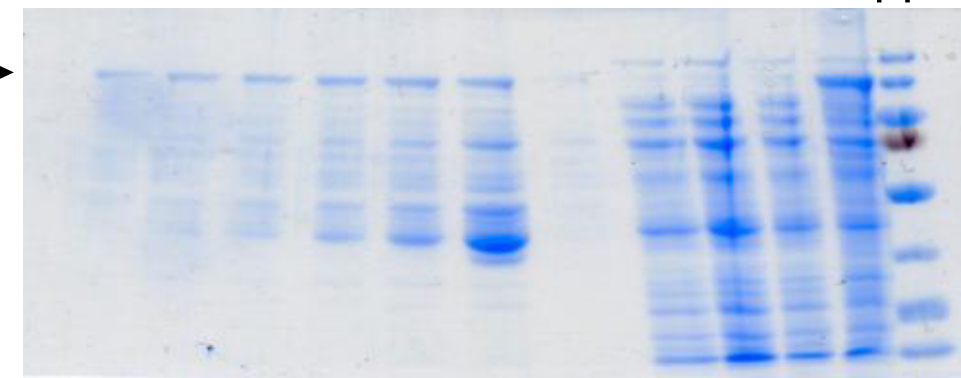

His_6-$ATM2_{CT}$ elution fraction used

M

37 kDa

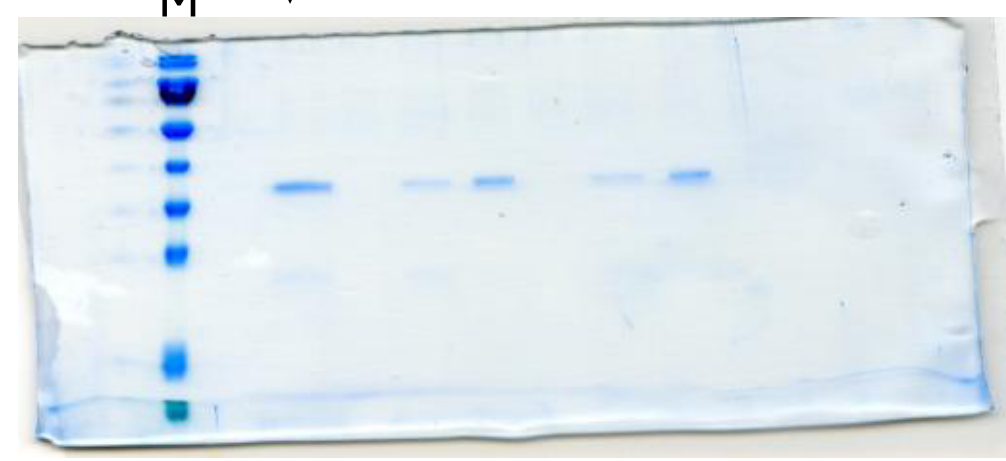

His_6-VIII-B_{CT} elution fraction used

M

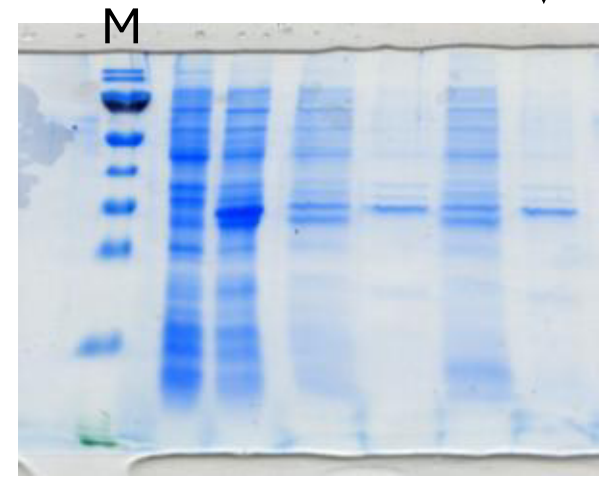

37 kDa

GST-ROP2 elution fraction used

M

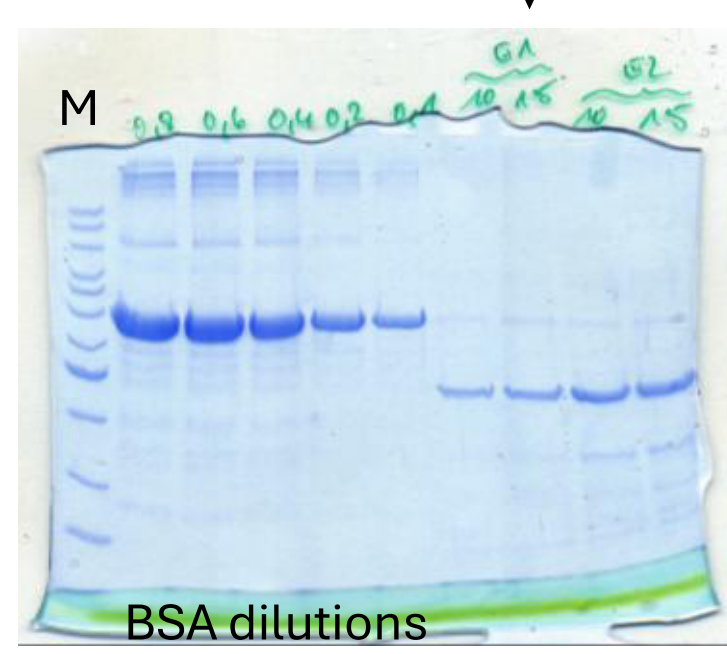

49 kDa

GST-$ROP2_{G15V}$ elution fraction used

M

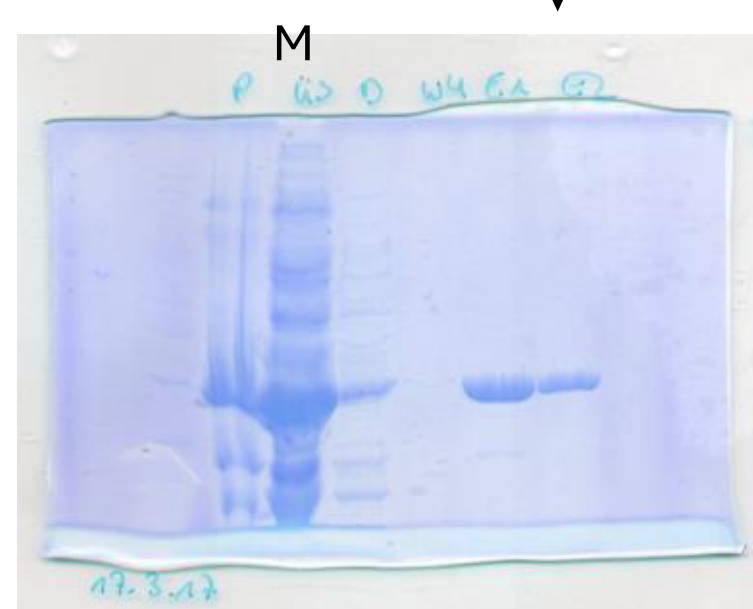

49 kDa

GST-$ROP2_{T20N}$ elution fraction used

M

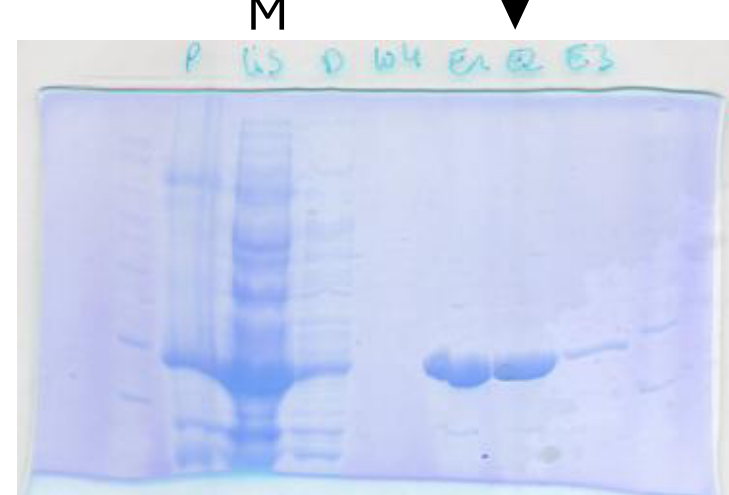

49 kDa
